# Disinformation elicits learning biases

**DOI:** 10.7554/eLife.106073

**Published:** 2026-06-26

**Authors:** Juan Vidal-Perez, Raymond J Dolan, Rani Moran

**Affiliations:** 1 Max Planck Centre for Computational Psychiatry and Ageing, University College London London United Kingdom; 2 Wellcome Centre for Human Neuroimaging, University College London London United Kingdom; 3 Department of Psychology, School of Biological and Behavioural Sciences, Queen Mary University of London London United Kingdom; University of Toronto Canada; Brown University United States

**Keywords:** reinforcement learning, biases, disinformation, misinformation, Bayesian, behavioural modelling, Human

## Abstract

In open societies, disinformation is often considered a threat to the very fabric of democracy. However, we know little about how disinformation exerts its impact, especially its influence on individual learning processes. Guided by the notion that disinformation exerts its pernicious effects by capitalizing on learning biases, we ask which aspects of learning from potential disinformation align with ideal ‘Bayesian’ principles, and which exhibit biases deviating from these standards. To this end, we harnessed a reinforcement learning framework, offering computationally tractable models capable of estimating latent aspects of a learning process as well as identifying biases in learning. In two experiments, participants completed a two-armed bandit task, where they repeatedly chose between two lotteries and received outcome-feedback from sources of varying credibility, who occasionally disseminated disinformation by lying about true choice outcome (e.g., reporting non-reward when a reward was truly earned or vice versa). Computational modelling indicated that learning increased in tandem with source credibility, consistent with ideal-Bayesian principles. However, we also observed striking biases reflecting divergence from idealized Bayesian learning patterns. Notably, in one experiment individuals learned from sources that should have been ignored, as these were known to be fully unreliable. Additionally, the presence of disinformation elicited exaggerated learning from trustworthy information (akin to jumping to conclusions) and exacerbated a normalized measure of ‘positivity bias’ whereby individuals self-servingly boost their learning from positive, relative to negative, choice feedback. Thus, in the face of disinformation we identify specific cognitive mechanisms underlying learning biases, with potential implications for societal strategies aimed at mitigating its harmful impacts.

## Introduction

Disinformation is a pervasive and pernicious feature of the modern world (World Economic Forum, 2024). It is linked to negative social impacts that include public-health risks (Carrieri et al., 2019; Rocha et al., 2023; Vox, 2017), political radicalization (Horta Ribeiro et al., 2017; Piazza, 2022), violence (Piazza, 2022; Roy et al., 2023; BBC News, 2016), and adherence to conspiracy theories (BBC News, 2016; Enders et al., 2023). Consequently, there is a growing interest in comprehending how false information propagates across social networks (Guess et al., 2019; Del Vicario et al., 2016; Shao et al., 2018), including an interest in designing strategies to curb its impact (Sharevski et al., 2022; Walter and Murphy, 2018; Globig et al., 2023; Roozenbeek and van der Linden, 2019) albeit with limited success to date (O’Mahony et al., 2023). However, there is also a considerable knowledge lacuna regarding how individuals learn and update their beliefs when exposed to potential disinformation. Addressing this gap is crucial, as it has been suggested that disinformation propagates by exploiting cognitive biases (Vosoughi et al., 2018; Modgil et al., 2021; Menczer and Ciampaglia, 2018; Pennycook et al., 2020; Swire et al., 2017). Thus, uncovering whether and how potential disinformation elicits distinct learning *biases* has the potential to better enable targeted interventions aimed at countering its harmful effects.

We start with an assessment of a prediction that individuals should modulate their learning as a function of the credibility of an information source, and learn more from credible, truthful, information sources. This prediction is based on Bayesian principles of learning and on previous findings showing that individuals flexibly and adaptively adjust their learning rates in response to key statistical features of the environment. For example, learning is more rapid when observation-uncertainty (‘noise’) decreases and in volatile, changing, compared to stable environments, particularly following detection of change points that render pre-change knowledge obsolete (Behrens et al., 2007; Nassar et al., 2010; Diederen and Schultz, 2015). Moreover, human choice is strongly influenced by social information of high (as opposed to low) credibility, such as majority opinions, more confident judgements (Campbell-Meiklejohn et al., 2017) and large group consensus (De Martino et al., 2017). Additionally, people are disposed to follow trustworthy advisors (Toelch et al., 2014), including those who have recommended optimal actions in the past (Biele et al., 2009; Vélez and Gweon, 2019).

We hypothesized that in a disinformation context individuals would show significant deviations from idealized Bayesian learning, reflecting a diversity of biases. First, filtering non-credible information is likely to be cognitively demanding (Jiwa et al., 2023), and this predicts such information would impact belief updating, even if individuals are aware it is untrustworthy. An additional consideration is that humans tend to learn more from positive self-confirming information (Sharot, 2011; Sharot and Garrett, 2016; Sharot et al., 2011), which presents one in a positive light. We conjectured, influenced by ideas from motivated cognition (Hughes and Zaki, 2015), that low-credibility information provides a pathway for amplification of such a bias, as uncertainty regarding information-veracity might dispose individuals to self-servingly interpret positive information as true and explain-away negative information as false. A final additional consideration is the question of how exposure to potential disinformation impacts on learning from trusted sources. One possibility is that disinformation serves as a background context against which credible information would appear more salient. Alternatively, it might lead individuals to strategically reduce their overall learning in disinformation-rich environments, resulting in diminished learning from credible sources.

To address these questions, we adopt a novel approach within the disinformation literature by exploiting a reinforcement learning (RL) experimental framework (Sutton and Barto, 1998). While RL has guided disinformation research in recent years (Schulz et al., 2023; Aston, 2022; Aymanns et al., 2022; Lindström et al., 2021; Vidal-Perez et al., 2025a), our approach is novel in using one of its most popular tasks: the ‘bandit task’. This has the advantage that it provides computationally tractable models that enable estimation of latent aspects of learning processes, such as belief updating. Moreover, our approach also enables an examination of the dynamics of belief updates over short timescales reflecting real-life engagements with disinformation, such as deciding whether to share a post on social media. Moreover, bandit tasks in RL have proven success in characterizing key decision-making biases (e.g., positivity bias; Palminteri et al., 2017; Lefebvre et al., 2017; Palminteri and Lebreton, 2022), albeit in scenarios where learners receive accurate information. Finally, a previous literature has suggested a role for reinforcement in the dissemination of disinformation, where individuals may receive positive reinforcement (likes, shares) for spreading sensationalized or misleading information on social media platforms, inadvertently reinforcing such behaviours and contributing to a disinformation proliferation (Globig et al., 2023; Lindström et al., 2021; Brady et al., 2021).

We developed a novel ‘disinformation’ version of the classical two-armed bandit task to test the effects of potential disinformation on learning. In the *traditional* two-armed bandit task (Sutton and Barto, 1998; Palminteri et al., 2017; Wilson and Collins, 2019), participants choose repeatedly between two unfamiliar bandits (i.e., slot machines) that provided rewards with different probabilities, to learn which bandit is more rewarding. Critically, in our *disinformation*-variant, true choice outcomes (reward or non-reward) were *latent*, i.e., unobservable. Instead, participants were informed about choice outcomes by computer-programmed ‘feedback agents’, who were disposed to occasionally disseminate disinformation by lying (reporting a reward when the true outcome was non-reward or vice versa). As these feedback agents varied in truthfulness, this allowed us to test the effects of source credibility on learning. We show across two studies that the extent of belief updates increases as a function of source credibility. However, there were striking deviations from ideal-Bayesian learning, where we identify several sources of bias related to processing potential disinformation. In one experiment, individuals learned from non-credible information that should in principle be ignored. Additionally, in both experiments, participants exhibited increased learning from trustworthy information when it was preceded by non-credible information and an amplified normalized positivity bias for non-credible sources, where individuals preferentially learn from positive compAared to negative feedback (relative to the overall extent of learning).

## Results

### Disinformation two-armed bandit task

We conducted a discovery (*n* = 104) and main study (*n* = 204). In both studies the learning tasks had the same basic structure but with a few subtle differences between them (see Appendix 1). To anticipate, the results of both studies support mostly similar conclusions, and in the results section, we focus on the main study, with the final results section detailing similarities and differences in findings across the two studies.

In the main study, participants (*n* = 204) completed the *disinformation* two-armed bandit task. In the *traditional* two-armed bandit task (Sutton and Barto, 1998; Palminteri et al., 2017; Wilson and Collins, 2019), participants choose between two slot machines (i.e., bandits) differing in their reward probability. Participants are not instructed about bandit reward probabilities but instead they are provided with veridical choice feedback (e.g., reward or non-reward), allowing participants to learn which bandit is more rewarding. By contrast, in our disinformation version *true* choice outcomes were latent (i.e., unobserved) and participants were informed about these outcomes via three computerized feedback agents, who had privileged access to the true outcomes.

**Figure 1.**
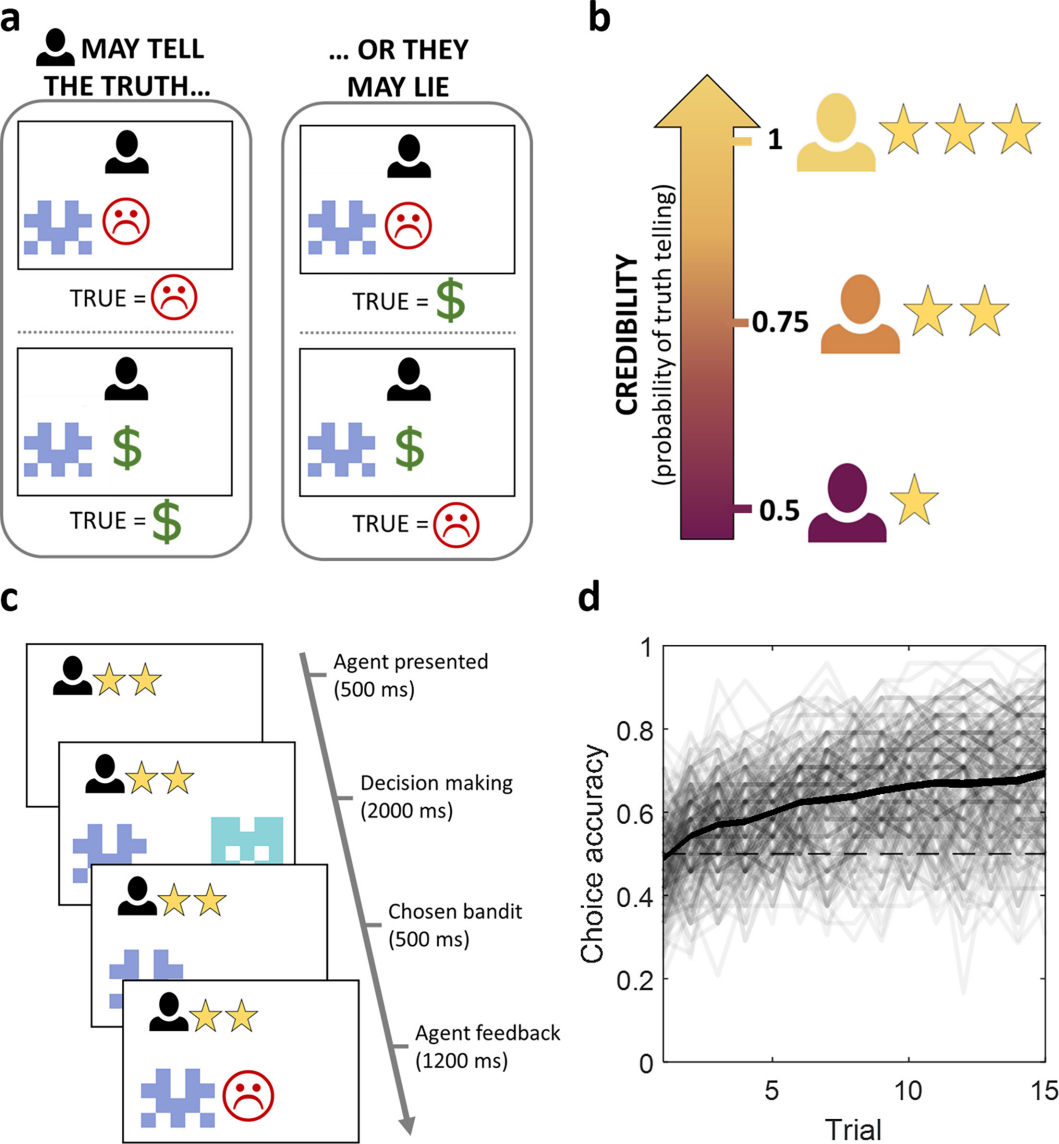
Task design and performance. (**a**) Illustration of agent feedback. Each selected bandit generated a *true* outcome, either a reward or a non-reward. Participants *did not* see this true outcome but instead were informed about it via a computerized feedback agent (reward: dollar sign; non-reward: sad emoji). Agents told the truth on most trials (left panel). However, on a random minority of trials they lied, reporting a reward when the true outcome was a non-reward or vice versa (right panel). (**b**) Participants received feedback from three distinct feedback agents of variable credibility (i.e., truth-telling probability). Credibility was represented using a star-based system: a 3-star agent always reported the truth (and never lied), a 2-star agent reported the truth on 75% of trials (lying on the remaining 25%), and a 1-star agent reported the truth half of the time (lying on the other half). Participants were explicitly instructed and quizzed about the credibility of each agent prior to the task. (**c**) Trial structure: On each trial participants were first presented with the feedback agent for that trial (here, the 2-star agent) and next offered a choice between a pair of bandits (represented by identicons) (for 2 s). Next, choice feedback was provided by the agent. (**d**) Learning curves. Average choice accuracy as a function of trial number (within a bandit pair). Thin lines: individual participants; thick line: group mean with thickness representing the group standard error of the mean for each trial.

Before commencing the task, participants were instructed that feedback agents could disseminate disinformation, meaning that they were disposed to lie on a random minority of trials, reporting a reward when the true outcome was a non-reward, or vice versa (Figure 1a). Participants were explicitly instructed about the credibility of each agent (i.e., based on the proportion of truth-telling trials), indicated by a ‘star system’: the 3-star agent was always truthful, the 2-star agents told the truth on 75% of the trials while the 1-star agent did so on 50% of the trials (Figure 1b). Note that while the 1-star agent’s feedback was statistically equivalent to random feedback, participants were not explicitly instructed about this equivalence. Each experimental block encompassed 3 bandit pairs, each presented over 15 trials in a randomly interleaved manner. The agent on each trial was random subject to the constraint that each agent provided feedback for five trials for each bandit pair. Thus, in every trial, participants were presented with one of the bandit pairs and the feedback agent associated with that trial. Upon selecting a bandit, they then received feedback from the agent (Figure 1c). Importantly, at the end of the experiment participants received a performance-based bonus based on *true* bandit outcomes, which could differ from agent provided feedback. Within each bandit pair one bandit provided a (true) reward on 75% of the trials and the other on 25% of trials. Choice accuracy, i.e., the probability of selecting the more rewarding bandit (within each pair), was significantly above chance (mean accuracy = 0.62, *t*(203) = 19.94, p < 0.001) and improved as a function of increasing experience with each bandit pair (average overall improvement over 15 trials = 0.22, *t*(203) = 19.95, p < 0.001) (Figure 1d).

### Credible feedback promotes greater learning

A hallmark of RL value learning is that participants are more likely to repeat a choice following positive compared to negative reward feedback (henceforth, ‘feedback effect on choice-repetition’). We tested a hypothesis, based on Bayesian reasoning, that this tendency would increase as a function of agent-credibility (Fig. 3a). Thus, in a binomial mixed-effects model we regressed choice-repetition (i.e., whether participants repeated their choice from the most recent trial featuring the same bandit pair; 0: switch; 1: repeat) on feedback valence (negative or positive) and agent-credibility (1-, 2-, or 3-star), where these are taken from the last trial featuring the same bandit pair (Methods for model specification). Feedback valence exerted a positive effect on choice-repetition (*b* = 0.72, *F*(1,2436) = 1369.6, p < 0.001) and interacted with agent-credibility (*F*(2,2436) = 307.11, p < 0.001), with a feedback effect being greater for more credible agents (3-star vs. 2-star: *b* = 0.91, *F*(1,2436) = 351.17; 3-star vs. 1-star: *b* = 1.15, *t*(2436) = 24.02; and 2-star vs. 1-star: *b* = 0.24, *t*(2436) = 5.34, all p’s < 0.001). Additionally, we found a positive effect of feedback for the 3-star agent (*b* = 1.41, *F*(1,2436) = 1470.2, p < 0.001), and a smaller effect of feedback for the 2-star agent (*b* = 0.49, *F*(1,2436) = 230.0, p < 0.001). These results support our hypothesis that learning increases as a function of information credibility (note that the feedback effect for the 1-star agent is examined below; see ‘Non-credible feedback elicits learning’).

**Figure 2.**
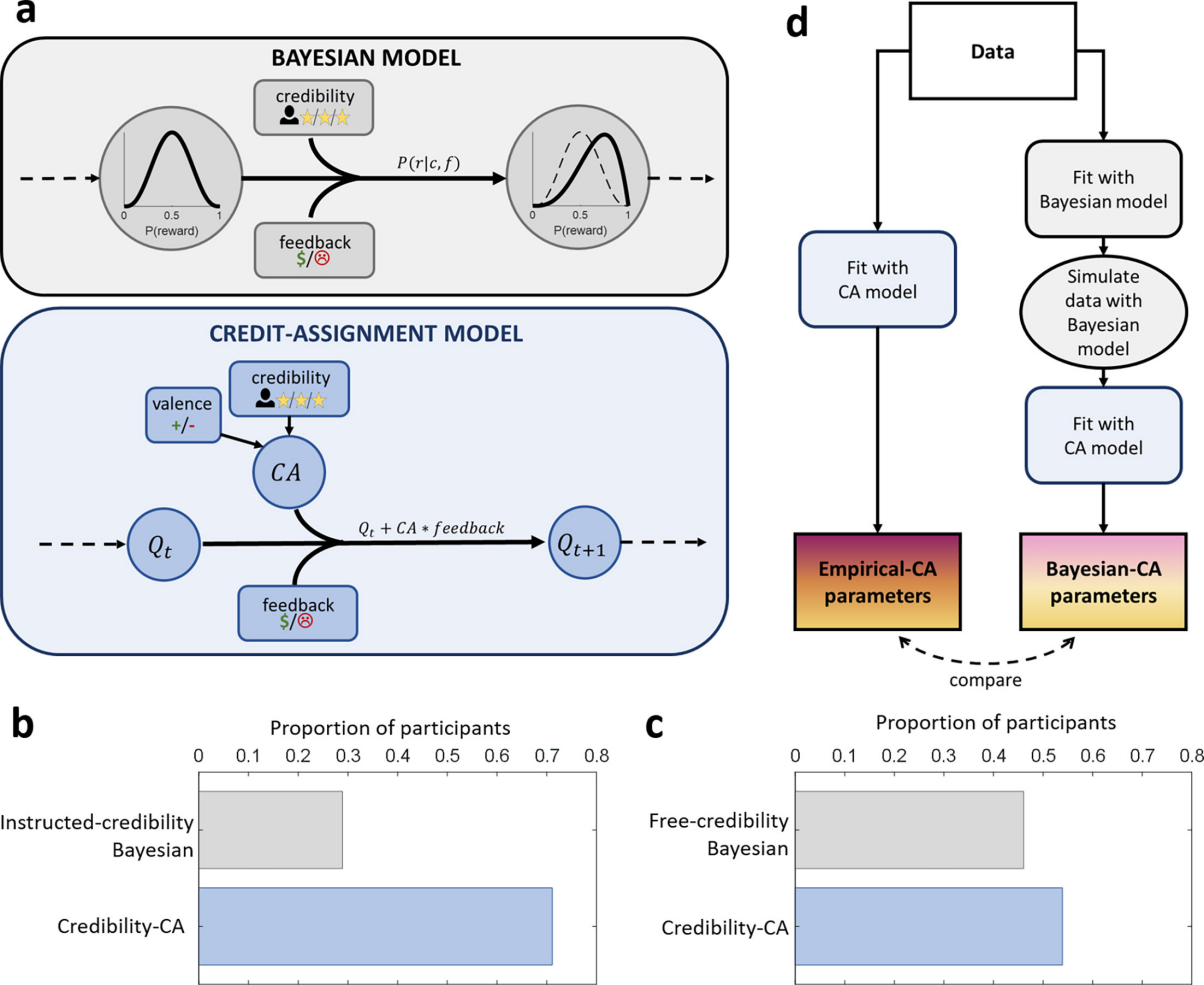
Computational models and cross-fitting method. (**a**) Summary of the two model families. In our Bayesian models (top panel), the observer maintains a belief distribution over the probability a bandit is *truly* rewarding (denoted *r*). On each trial, this distribution is updated for the selected bandit according to Bayes‘ rule, based on the valence (i.e., rewarding/non-rewarding; denoted *f*) and credibility of the trial’s reward feedback (denoted *c*). In credit assignment models (bottom panel), the observer maintains a subjective point value (denoted *Q*) reflecting a choice propensity for each bandit. On each trial the propensity of the chosen bandit is updated based on a free CA parameter, quantifying the extent of value increase/decrease following positive/negative feedback. CA parameters can be modulated by the valence and credibility of feedback. (**b, c**) Model selection between the credibility-CA model (without perseveration) and the two variants of Bayesian models. Most participants were best fitted by a credibility-CA model, compared to the instructed-credibility Bayesian model (**b**) or free-credibility Bayesian (**c**) models. (**d**) Cross-fitting method: Firstly, we fit a Bayesian model to empirical data, to estimate its (ML) parameters. This yields the Bayesian learning token that comes closest to accounting for a participant’s choices. Secondly, we simulate synthetic data based on the Bayesian model, using its ML parameters to obtain instances of how a Bayesian learner would behave in our task. Thirdly, we fit these synthetic data with a CA model, thus estimating ‘Bayesian CA parameters’, i.e., CA parameters capturing the performance of a Bayesian model. Finally, we fit the CA model directly to empirical data to obtain ‘empirical CA parameters’. A comparison of Bayesian and empirical CA parameters allows us to identify which aspects of behaviour are consistent with our Bayesian models, as well as characterize biases in behaviour that deviate from our Bayesian learning models.

**Figure 3.**
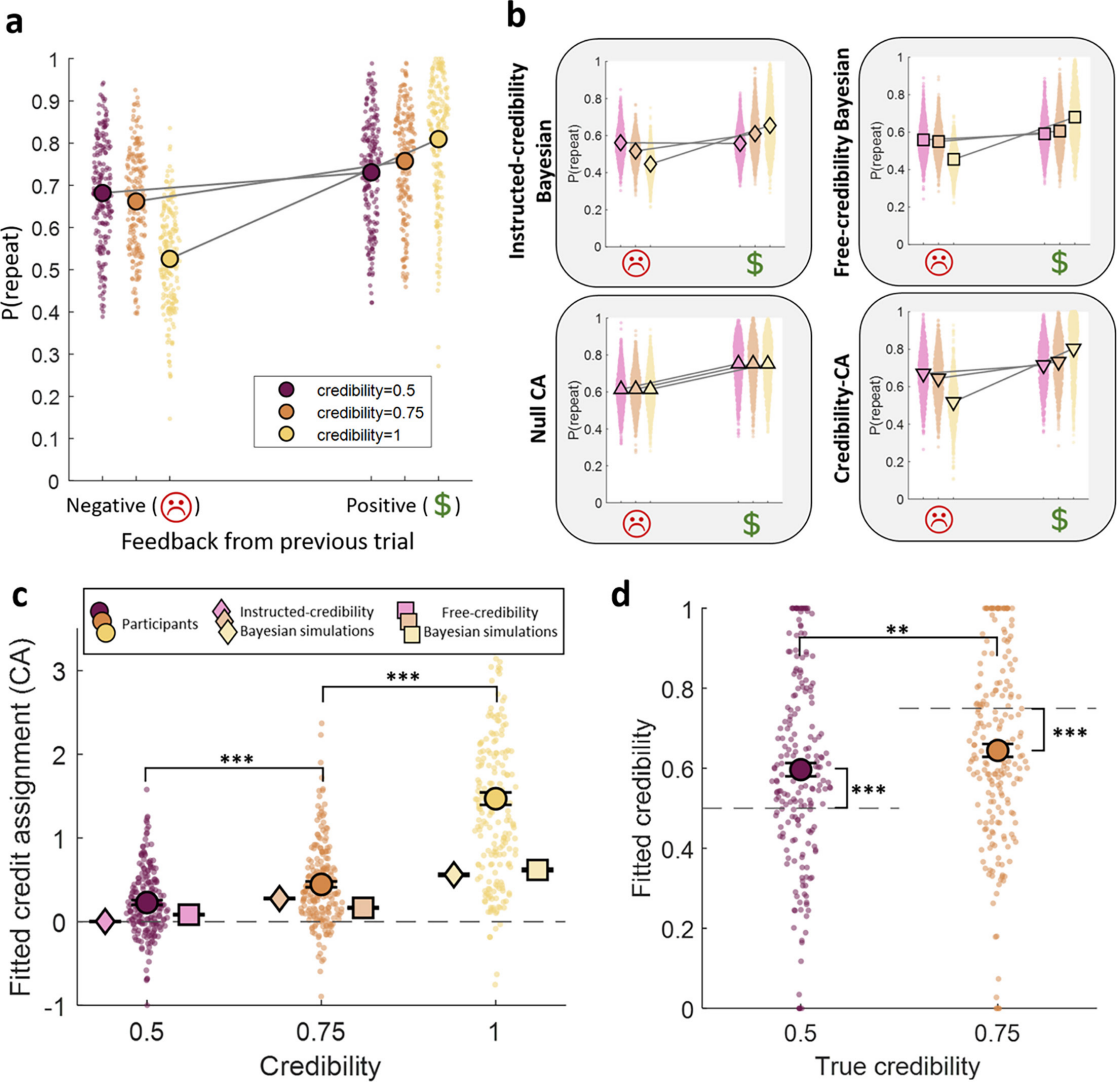
Learning adaptations to credibility. (**a**) Probability of repeating a choice as a function of feedback valence and agent-credibility on the previous trial for the same bandit pair. The effect of feedback valence on repetition increases as the feedback credibility increases, indicating that more credible feedback has a greater effect on behaviour. (**b**) Similar analysis as in panel a, but for synthetic data obtained by simulating the main models. Simulations were computed using the ML parameters of participants for each model. The null model (**bottom left**) attributes a single CA to all credibility levels, hence feedback exerts a constant effect on repetition (independently of its credibility). The credibility-CA model (**bottom-right**) allowed credit assignment to change as a function of source credibility, predicting varying effects of feedback with different credibility levels. The instructed-credibility Bayesian model (**top left**) updated beliefs based on the true credibility of the feedback, and therefore predicted an increase effect of feedback on repetition as credibility increased. Finally, the free-credibility Bayesian model (**top right**) allowed for a possibility that participants use distorted credibilities for 1-star and 2-star agents when following a Bayesian strategy, also predicting an increase in the effect of feedback as credibility increased. (**c**) ML credit assignment parameters for the credibility-CA model. Participants show a CA increase as a function of agent-credibility, as predicted by Bayesian-CA parameters for both the instructed-credibility and free-credibility Bayesian models. Moreover, participants showed a positive CA for the 1-star agent (which essentially provides random feedback), which is only predicted by cross-fitting parameters for the free-credibility Bayesian model. (**d**) ML credibility parameters for a free-credibility Bayesian model attributing credibility 1 to the 3-star agent but estimating credibility for the two lying agents as free parameters. Small dots represent results for individual participants/simulations, big circles represent the group mean (**a, b, d**) or median (**c**) of participants’ behaviour. Results of the synthetic model simulations are represented by diamonds (instructed-credibility Bayesian model), squares (free-credibility Bayesian model), upward-pointing triangles (null-CA model) and downward-pointing triangles (credibility-CA model). Error bars show the standard error of the mean. *p < 0.05, **p < 0.01, ***p < 0.001.

To confirm that increased learning based on information credibility is expected under an assumption that subjects adhere to Bayesian reasoning, we formulated two Bayesian models whereby the latent value of each bandit is represented as a distribution over the probability that a bandit is truly rewarding (Figure 2a, top panel; Appendix 2—figure 1c for an illustration of the model; for full model descriptions, see Methods). In the *instructed-credibility Bayesian* model, belief updates are based on the *instructed*-credibility of feedback sources. This model is based on an idealized assumptions that during the feedback stage of each trial, the value of the chosen bandit is updated (based on feedback valence and credibility) according to Bayes' rule reflecting perfect adherence to the instructed task structure (i.e., how true outcomes and feedback are generated). In contrast, a *free-credibility Bayesian* model allows for the possibility that Bayes-rule updates during feedback are based on ‘distorted probabilities’ (Reyna and Brainerd, 2023), attributing *non-instructed* degrees of credibility to sources of false information (despite our explicit instructions on the credibility of different agents). In this variant, we fixed the credibility of the 3-star agent to 1 and estimated the credibility of 2 and 1-star agents as free parameters (which were highly recoverable; see Methods and Appendix 4—figure 9). Both models additionally assumed uninformative, uniform priors over reward probabilities of novel bandits and that learning is non-forgetful. Simulations based on both Bayesian models (see Methods) predicted increased learning as a function of feedback credibility (Figure 3b; top panels; Appendix 3—tables 2 and 3 for statistical analysis).

Next, we formulated a family of *non-Bayesian* computational RL models. Importantly, these models can flexibly express non-Bayesian learning patterns and, as we show in following sections, can serve to identify learning biases deviating from an idealized Bayesian strategy. Here, an assumption is that during feedback, the choice propensity for the chosen bandit (which here is represented by a point estimate, ‘*Q* value’, rather than a distribution) either increases or decreases (for positive or negative feedback, respectively) according to a magnitude quantified by the free ‘credit assignment (CA)’ model parameters (Moran et al., 2021):

$$\displaystyle Q\left (chosen\right)\leftarrow \left (1\mathrm{-}f_{Q}\right)\mathrm{*}Q\left (chosen\right)\mathrm{+}CA\left (agent,valence\right)\mathrm{*}F$$

where *F* is the feedback received from the agents (coded as 1 for reward feedback and –1 for non-reward feedback), while *f_Q_* (∈[0,1]) is the free parameter representing the forgetting rate of the *Q*-value (Figure 2a, bottom panel; Appendix 2—figure 1b; see ‘Methods: RL models’). The probability to choose a bandit (say A over B) in this family of models is a logistic function of the contrast choice propensities between these two bandits. One interpretation of this model is as a logistic regression, where the CA parameters take the role of regression coefficients corresponding to the change in log odds of repeating the just-taken action in future trials based on the feedback (+/−CA for positive or negative feedback, respectively; the model also includes gradual perseveration which allows for constant log-odd changes that are not affected by choice feedback). The forgetting rate captures the extent to which the effect of each trial on future choices diminishes with time. The *Q*-values are thus exponentially decaying sums of logistic choice propensities based on the types of feedback a bandit received.

Within this model-family, different model variants varied as to how task variables influenced CA parameters with the ‘null’ model attributing the same CA to all feedback agents (regardless of their credibility, i.e., a single free CA parameter), whereas the ‘credibility-CA’ model availed of three separate CA parameters, one for each feedback agent, thereby allowing us to test how learning was modulated by feedback credibility. Using a bootstrap generalized-likelihood ratio test for model-comparison (Methods) we rejected the null model (group level: p < 0.001), in favour of the credibility-CA model. Furthermore, model simulations based on participants best-fitting parameters (Methods) falsified the null model as it failed to predict credibility-modulated learning, showing instead, equal learning from all feedback sources (Figure 3b; bottom-left panel). In contrast, the credibility-CA model successfully predicted increased learning as a function of credibility (Figure 3b, bottom-right panel) (see Appendix 3—tables 4 and 5).

After confirming CA parameters are highly recoverable (see Methods and Appendix 4—figure 10), we examined how the maximum likelihood (ML) CA parameters from the credibility-CA model differed as a function of feedback credibility (Figure 3c; see Appendix 4—Section 3. 1 Main study for detailed ML parameter results). Using a mixed-effects model (Methods), we regressed the CA parameters on their associated agents, finding that CA differed across the agents (*F*(2,609) = 212.65, p < 0.001), increasing as a function of agent-credibility (3-star vs. 2-star: *b* = 1.02, *F*(1,609) = 253.73; 3-star vs. 1-star: *b* = 1.24, *t*(609) = 19.31; and 2-star vs. 1-star: *b* = 0.22, *t*(609) = 3.38, all p’s < 0.001).

### Substantial deviations from our Bayesian learning models

We next implemented a model comparison between each of our Bayesian models and the credibility-CA model, using a parametric bootstrap cross-fitting method (PBCM; Methods). We found that the credibility-CA model provided a superior fit for 71% of participants (sign test; p < 0.001) when compared to the instructed-credibility Bayesian model, Figure 2b; and for 53.9% (p = 0.29) when compared to the free-credibility Bayesian model, Figure 2c. We considered using AIC and BIC, which apply ‘off-the shelf’ penalties for model complexity. However, these methods do not adapt to features like finite sample size (relying instead on asymptotic assumption) or temporal dependence (as is common in RL experiments). In contrast, the PBCM replaces these fixed penalties with empirical, data-driven criteria for model selection. Indeed, model-recovery simulations confirmed that whereas AIC and BIC were heavily biased in favour of the Bayesian models, the bootstrap method provided excellent model-recovery (see Appendix 4—figure 12).

To further characterize deviations between behaviour and our Bayesian learning models, we used a ‘cross-fitting’ method. Treating CA parameters as data features of interest (i.e., feedback dependent changes in choice propensity), our goal was to examine if and how empirical features differ from features extracted from simulations of our Bayesian learning models. Towards that goal, we simulated synthetic data based on *Bayesian* agents (using participants’ best fitting parameters), but fitted these data using the CA models, obtaining what we term ‘Bayesian-CA parameters’ (Figure 2d; Methods). A comparison of these Bayesian-CA parameters, with empirical-CA parameters obtained by fitting CA models to empirical data, allowed us to uncover patterns consistent with, or deviating from, ideal-Bayesian value-based inference. Under the logistic-regression interpretation of the CA-model family the cross-fitting method comprises a comparison between empirical regression coefficients (i.e., empirical CA parameters) and regression coefficients based on simulations of Bayesian models (Bayesian-CA parameters). Using this approach, we found that both the instructed-credibility and free-credibility Bayesian models predicted increased Bayesian-CA parameters as a function of agent-credibility (Figure 3c; Appendix 3—table 7 and 8). However, an in-depth comparison between Bayesian and empirical CA parameters revealed discrepancies from ideal-Bayesian learning, which we describe in the following sections.

### Non-credible feedback elicits learning

While our task instructions framed the 1-star agent as highly deceptive, lying 50% of the time, its feedback is statistically equivalent to entirely non-informative i.e., *random* feedback. Thus, participants should ignore and filter-out such feedback from their belief updates. Indeed, for the 1-star agent, simulations based on the instructed-credibility Bayesian model provided no evidence for either a positive effect of feedback on choice-repetition (mixed-effects model described above; *b* = −0.01, *t*(2436) = −0.41, p = 0.68; Figure 3b, top-left) or a positive Bayesian-CA (b = −0.01, *t*(609) = −0.31, p = 0.76; Figure 3c). However, contrary to this, we hypothesized that participants would struggle to entirely disregard non-credible feedback. Indeed, we found a positive effect of feedback on choice-repetition for the 1-star agent (mixed-effects model, delta(*M*) = 0.049, *b* = 0.25, *t*(2436) = 8.05, p < 0.001), indicating participants are more likely to repeat a bandit selection after receiving positive feedback from this agent (Figure 3a). Similarly, the CA parameter for the 1-star agent in the credibility-CA model was positive (*b* = 0.23, *t*(609) = 4.54, p < 0.001) (Figure 3c). The upshot of this empirical finding is that participants updated their beliefs based on random feedback (see Appendix 2—figure 3 for analysis showing that this resulted in decreased accuracy rates).

A potential explanation for this finding is that participants *do* rely on a Bayesian strategy but ‘distort probabilities’, attributing non-instructed degrees of credibility to lying sources (despite our explicit instructions on the credibility of different agents). Consistent with this, the ML-estimated credibility of the 1-star agent (Figure 3d) was significantly greater than 0.5 (Wilcoxon signed-rank test, median = 0.08, *z* = 5.50, p < 0.001), allowing the free-credibility Bayesian model to predict a positive feedback effect on choice-repetition (mixed-effects model: *b* = 0.12, *t*(2436) = 9.48, p < 0.001; Figure 3b, top-right) and a positive Bayesian-CA (*b* = 0.08, *t*(609) = 3.32, p < 0.001; Figure 3c) for the 1-star agent. In our Discussion we elaborate on why it might be difficult to filter out this feedback even if one can explicitly infer its randomness.

### Increased learning from fully credible feedback when it follows non-informative feedback

**Figure 4.**
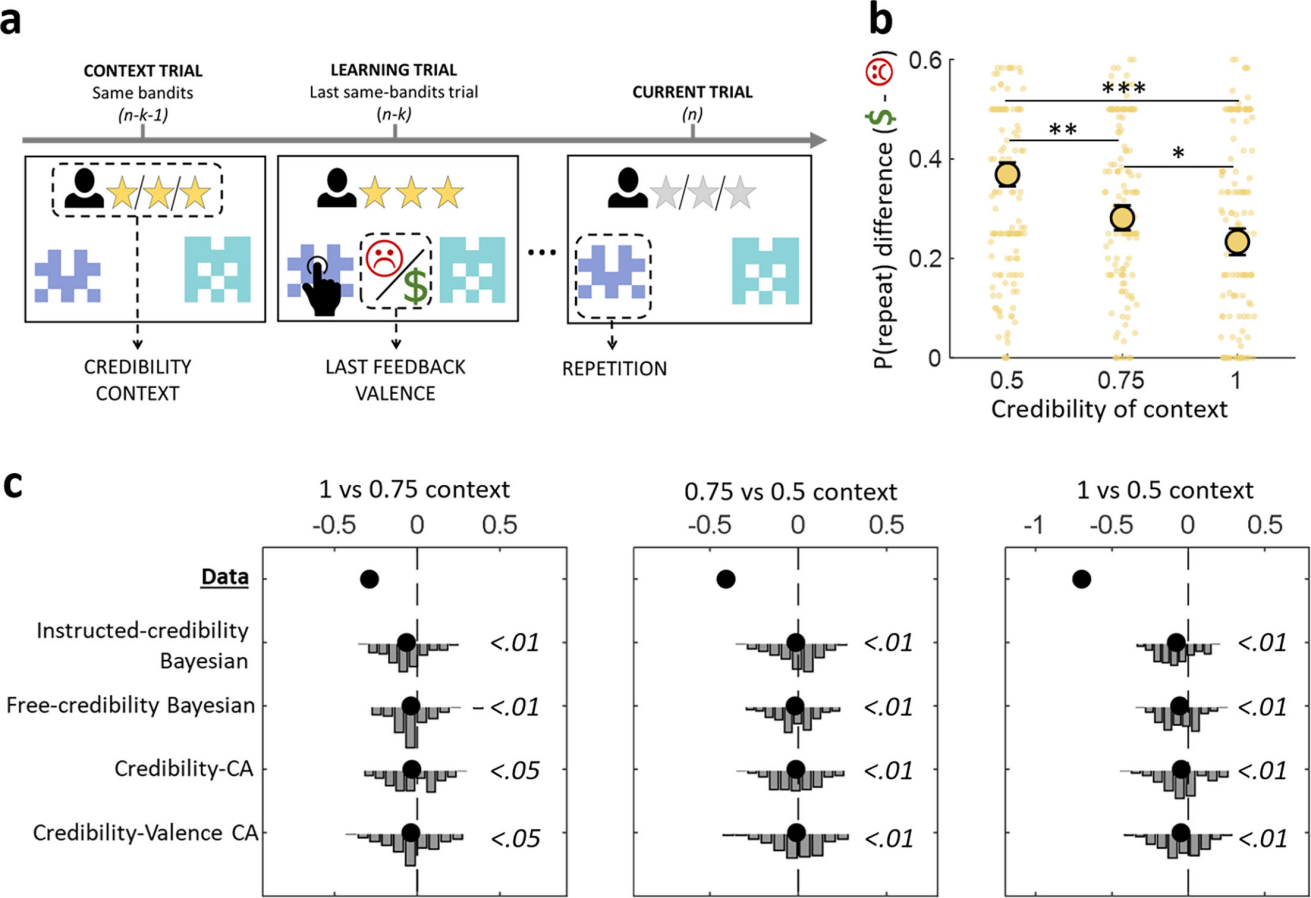
Contextual effects and learning. (**a**) Trials contributing to the analysis of effects of credibility-context on learning from the fully credible agent. We included only ‘current trials (n)’ for which: (1) the last trial (trial n-k) offering the same bandit pair (i.e., the learning trial) was associated with the 3-star agent, and (2) the immediately preceding context trial (*n − k −* 1) featured the same bandit pair. We examined how choice-repetition (from *n − k* to *n*) was modulated by feedback valence on the learning trial, and by the feedback agent on the context trial. Note the greyed-out star-rating on the current trial indicates the identity of the current agent and was not included in the analysis. (**b**) Difference in probability of repeating a choice after receiving positive vs. negative feedback (i.e., feedback effect) from the 3-star agent, as a function of the credibility context. The 3-star agent feedback-effect is greater when preceded by a lower-credibility context, compared to a higher credibility context. Big circles represent the group mean, and error bars show the standard error of the mean. *p < 0.05, **p < 0.01. (**c**) We ran the same mixed-effects model (regressing choice-repetition of learning-trial feedback valence and on contextual credibility) on simulated data (see Methods: Model-agnostic analysis of contextual credibility effects on choice-repetition). The panels show contrasts in feedback effect (from the 3-star agent in the learning trial) on choice-repetition between contextual credibility agent pairs. None of our models predicted the contrast effects observed in participants. Histograms represent the distribution of regression coefficients based on 101 group-level synthetic datasets, simulated based on each model. The label right to each histograms represents the proportion of simulated datasets that predict an equal or stronger effect than the one observed in participants.

A comparison of empirical and Bayesian credit assignment parameters revealed a further deviation from ideal-Bayesian learning: participants showed an exaggerated credit assignment for the 3-star agent compared with Bayesian models [Wilcoxon signed-rank test, instructed-credibility Bayesian model (median difference = 0.74, *z* = 11.14); free-credibility Bayesian model (median difference = 0.62, *z* = 10.71), all p’s < 0.001] (Figure 3a). One explanation for enhanced learning for the 3-star agents is a contrast effect, whereby credible information looms larger against a backdrop of non-credible information. To test this hypothesis, we examined whether the impact of feedback from the 3-star agent is modulated by the credibility of the agent in the trial immediately preceding it. More specifically, we reasoned that the impact of a 3-star agent would be amplified by a ‘low-credibility context’ (i.e., when it is preceded by a low-credibility trial). In a binomial mixed-effects model, we regressed choice-repetition on feedback valence from the last trial featuring the same bandit pair (i.e., the learning trial) and the feedback agent on the trial immediately preceding that last trial (i.e., the contextual credibility; see Methods for model specification). This analysis included only learning trials featuring the 3-star agent, and context trials featuring the same bandit pair as the learning trial (Figure 4a). We found that feedback valence interacted with contextual credibility (*F*(2,2086) = 11.47, p < 0.001) such that the feedback effect (from the 3-star agent) decreased as a function of the preceding context-credibility (3-star context vs. 2-star context: *b* = –0.29, *F*(1,2086) = 4.06, p = 0.044; 2-star context vs. 1-star context: *b* = −0.41, t(2086) = −2.94, p = 0.003; and 3-star context vs. 1-star context: *b* = −0.69, *t*(2086) = −4.74, p < 0.001) (Figure 4b). This contrast effect was not predicted by simulations of our main models of interest (Figure 4c). No effect was found when focussing on contextual trials featuring a bandit pair different than the one in the learning trial (see Appendix 5). Thus, these results support an interpretation that credible feedback exerts a greater impact on participants’ learning when it follows non-credible feedback in the same learning context.

### Positivity bias in learning and credibility

Previous research has shown that RL is characterized by a positivity bias, wherein subjects systematically learn more from positive than from negative feedback (Palminteri et al., 2017; Palminteri and Lebreton, 2022). One account is that this bias might result from motivated cognition influences on learning, whereby participants favour positive feedback that reflects well on their choices. We conjectured that feedback of ambiguous veracity (i.e., from the 1-star and 2-star agents) would promote this bias by allowing participants to explain-away negative feedback as a case of an agent-lying, while choosing to believe positive feedback. Following previous research, we quantified positivity bias in 2 ways: (1) as the *absolute* difference between credit assignment based on positive or negative feedback, and (2) as the same difference but *relative* to the overall extent of learning. We note that the second, relative, definition, is more akin to ‘percentage change’ measurements providing a control for the overall lower levels of credit assignment for less credible agents. To investigate this bias across different levels of feedback credibility we formulated a more detailed variant of the CA model. To quantify the extent of a chosen-bandit’s value increase or decrease—following positive or negative feedback, respectively—the ‘credibility-valence-CA’ variant included separate CA parameters for positive (CA^+^) and negative (CA^−^) feedback for each feedback agent. In effect, this model variant enabled us to test whether different levels of feedback credibility elicited a positivity bias (i.e., CA^+^ > CA^−^). Using a bootstrap generalized-likelihood ratio test for model comparison (Methods), we rejected, in favour of the valence-credibility-CA model, the null-CA model, the credibility-CA model and a ‘constant feedback-valence bias’ CA model, which attributed a common valence bias (CA^+^ − CA^−^) to all agents (all group level: all p’s < 0.001). This test supported our choice of flexible CA parametrization as a factorial function of agent and feedback valence.

**Figure 5.**
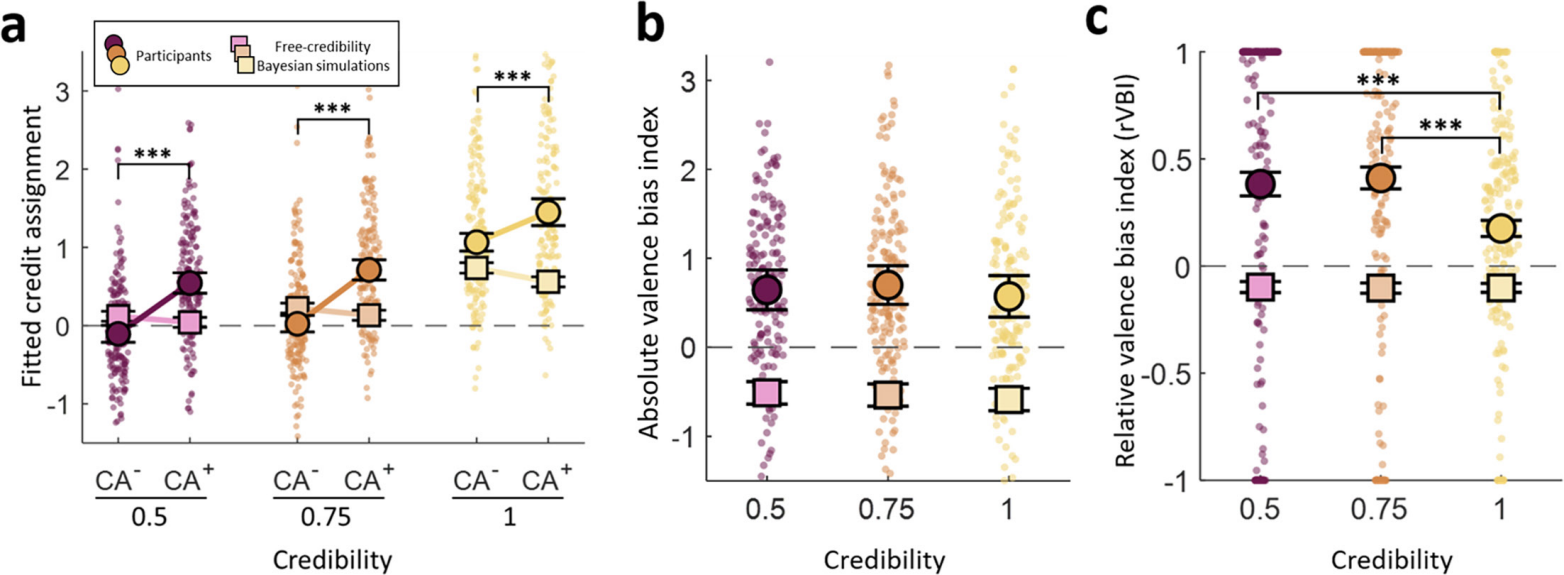
Positivity bias as a function of agent-credibility. (**a**) ML parameters from the credibility-valence-CA model. CA^+^ and CA^−^ are free parameters representing credit assignments for positive and negative feedback, respectively (for each credibility level). Our data revealed a positivity bias (CA^+^ > CA^−^) for all credibility levels. (**b**) Absolute valence bias index (defined as CA^+^ − CA^−^) based on the ML parameters from the credibility-valence CA model. Positive values indicate a positivity bias, while negative values represent a negativity bias. (**c**) Relative valence bias index (defined as (CA^+^ − CA^−^)/(|CA^+^| + |CA^−^|)) based on the ML parameters from the credibility-valence CA model. Positive values indicate a positivity bias, while negative values represent a negativity bias. Small dots represent fitted parameters for individual participants and big circles represent the group median (**a, b**) or mean (**c**) (both of participants’ behaviour), while squares are the median or mean of the fitted parameters of the free-credibility Bayesian model simulations. Error bars show the standard error of the mean. ***p < 0.001 for ML fits of participants behaviour.

After confirming the parameters of this model were highly recoverable (see Methods and Appendix 4—figure 11), we used a mixed-effects model to regress the ML parameters (Figure 5a; see Appendix 4—Section 3. 1 for detailed ML parameter results) on their associated agent-credibility and valence (see Methods). This revealed participants attributed a greater CA to positive feedback than to negative feedback (*b* = 0.64, *F*(1,1218) = 37.39, p < 0.001). Strikingly, for lying agents, participants selectively assigned credit based on positive feedback (1-star: *b* = 0.61, *F*(1,1218) = 22.81, p < 0.001; 2-star: *b* = 0.85, *F*(1,1218) = 43.5, p < 0.001), with no evidence for significant credit assignment based on negative feedback (1-star: *b* = −0.03, *F*(1,1218) = 0.07, p = 0.79; 2-star: *b* = 0.14, *F*(1,1218) = 1.28, p = 0.25). Only for the 3-star agent, credit assignment was positive for both positive (*b* = 1.83, *F*(1,1218) = 203.1, p < 0.001) and negative (*b* = 1.25, *F*(1,1218) = 95.7, p = <0.001) feedback. We found no significant interaction effect between feedback valence and credibility on CA (*F*(2,1218) = 0.12, p = 0.88; Figure 5a, b). Thus, there was no evidence for our hypothesis when positivity bias was measured in absolute terms.

However, we found evidence for agent-based modulation of positivity bias when this bias was measured in relative terms. Here we calculated, for each participant and agent, a relative Valence Bias Index (rVBI) as the difference between the Credit Assignment for positive feedback (CA^+^) and negative feedback (CA^−^), relative to the overall magnitude of CA (i.e., |CA^+^| + |CA^−^|) (Figure 5c). Using a mixed-effects model, we regressed rVBIs on their associated credibility (see Methods), revealing a relative positivity bias for all credibility levels [overall rVBI (*b* = 0.32, *F*(1,609) = 68.16), 50% credibility (*b* = 0.39, *t*(609) = 8.00), 75% credibility (*b* = 0.41, *F*(1,609) = 73.48) and 100% credibility (*b* = 0.17, *F*(1,609) = 12.62), all p’s<0.001]. Critically, the rVBI varied depending on the credibility of feedback (*F*(2,609) = 14.83, p < 0.001), such that the rVBI for the 3-star agent was lower than that for both the 1-star (*b* = −0.22, *t*(609) = −4.41, p < 0.001) and 2-star agent (*b* = −0.24, *F*(1,609) = 24.74, p < 0.001). Feedback with 50% and 75% credibility yielded similar rVBI values (*b* = 0.028, *t*(609) = 0.56, p = 0.57). Finally, a positivity bias could not stem from a Bayesian strategy as both Bayesian models predicted a negativity bias (Figure 5b, c; Appendix 2—figure 4; Appendix 3—table 10 and 11; Appendix 4—table 1-4). Taken together, this provides equivocal support for our initial hypothesis, depending on the measurement scale used to assess the effect (absolute or relative).

Previous research has suggested that positivity bias may spuriously arise from pure choice-perseveration (i.e., a tendency to repeat previous choices regardless of outcome) (Sugawara and Katahira, 2021; Palminteri, 2023). While our models included a perseveration component, we acknowledge this control is not perfect (Vidal-Perez et al., 2025b). Therefore, in additional control analyses, we generated (using ex post simulations based on best fitting parameters) synthetic datasets using models including choice-perseveration, but devoid of feedback-valence bias, and fitted these with our credibility-valence model (see Appendix 5—Section 1 Contrast effects for contexts featuring a 651 different bandit). These analyses confirmed that a pure perseveration account can masquerade as an apparent positivity bias, and even predict the qualitative pattern of results related to credibility (i.e., a higher relative positivity bias for low-credibility feedback). Critically, however, this account consistently predicted a reduced magnitude of credibility effect on relative positivity bias as compared to the one we observed in participants, suggesting at least some of the relative amplification of positivity bias goes above and beyond contributions from perseveration.

### True feedback elicits greater learning

**Figure 6.**
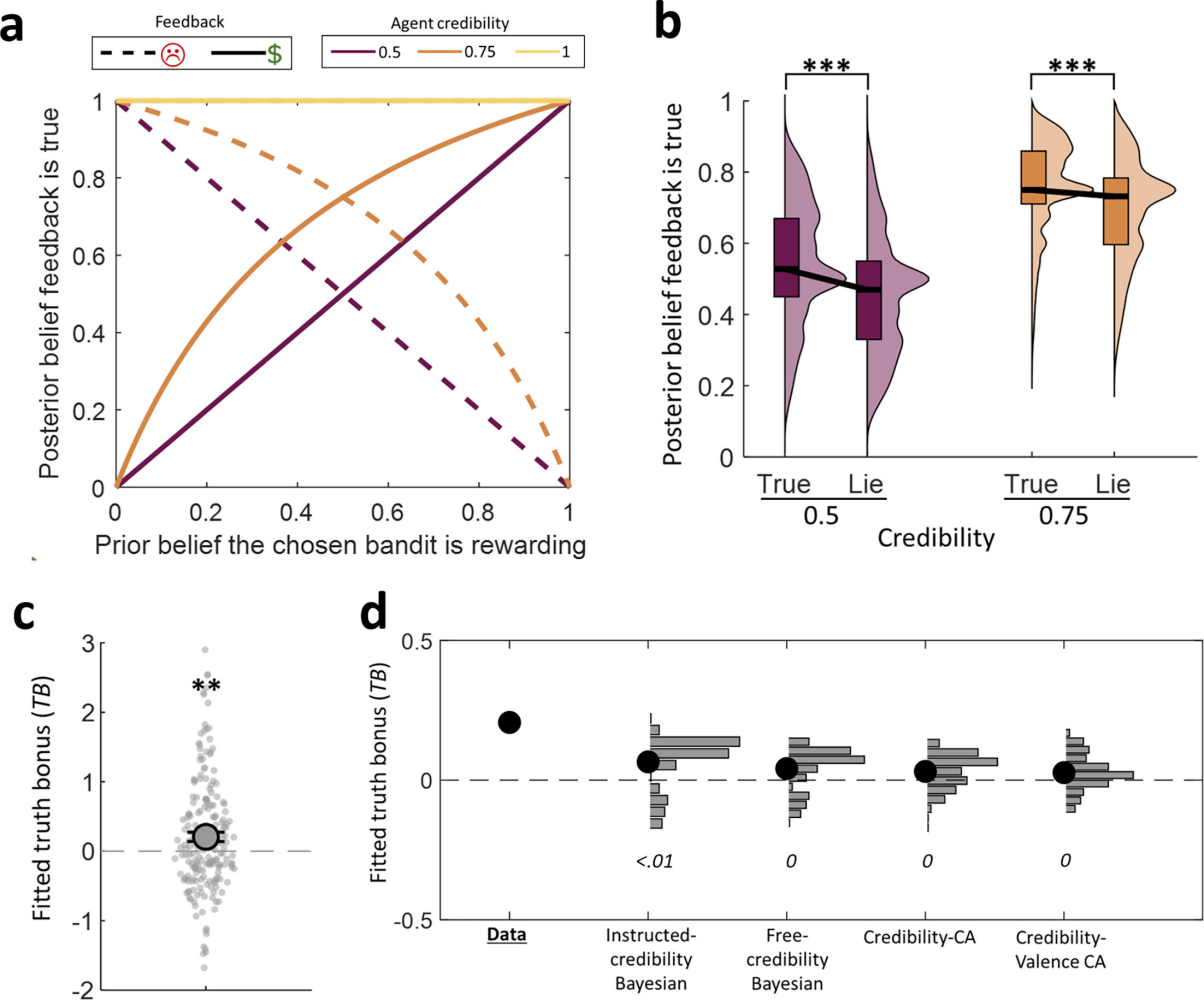
Credit assignment is enhanced for feedback that is more likely to be true. (**a**) The posterior belief that the received feedback is truthful (*y*-axis) is plotted against the prior belief (held before receiving feedback) that the chosen bandit would be rewarding (*x*-axis). The plot illustrates how this posterior belief is influenced by the valence of the feedback (reward indicated by solid lines, no reward by dashed lines) and the credibility of the feedback agent (represented by different colours). (**b**) Distribution of posterior belief probability that feedback is true, calculated separately for each agent (1-star or 2-star) and objective feedback truthfulness (true or lie). These probabilities were computed based on trial sequences and feedback participants experienced, indicating belief probabilities that feedback is true are higher in truth compared to lie trials. For illustration, plotted distributions pool trials across participants. The black line within each box represents the median, upper and lower bounds represent the third and first quartiles, respectively. The width of each half-violin plot corresponds to the density of each posterior belief value among all trials for a given condition. (**c**) Maximum likelihood (ML) estimate of the ‘truth-bonus’ parameter derived from the ‘Truth-CA‘ model. The significantly positive truth bonus indicates that participants increased credit assignment as a function of the likelihood this feedback was true (after controlling for the credibility of this feedback). Each small dot represents the fitted truth-bonus parameter for an individual participant, the large circle indicates the group mean, and the error bars represent the standard error of the mean. (**d**) Distribution of truth-bonus parameters predicted by synthetic simulations of our alternative computational models. For each alternative model, we generated 101 synthetic group-level datasets based on the maximum likelihood parameters fitted to the participants' actual behaviour. Each of these datasets was then independently fitted with the ‘Truth-CA’ model. Each histogram represents the distribution of the mean truth bonus across the 101 simulated group-level datasets for a specific alternative model. Notably, the truth bonus observed in our participants was significantly higher than the truth bonus predicted by any of these alternative models (proportion of datasets predicting a higher truth bonus: instructed-credibility Bayesian <0.01, free-credibility Bayesian = 0, credibility-CA = 0, credibility-valence CA = 0). **p < 0.01.

Our findings are consistent with participant modulation of the extent of credit assignment based *solely* on cued task variables, such as feedback credibility and valence. However, we also considered another possibility: that participants might infer, on a *trial-by-trial* basis, whether the feedback they received was true or false and adjust their credit assignment based on this inference. For example, for a given feedback agent, participants might boost the credit assigned to a chosen bandit as a function of the degree to which they believe feedback was true. Notably, Bayesian inference can support a trial-level calculation of a posterior probability that feedback is true based on its credibility, valence and a prior belief (based on experiences in previous trials) regarding the probability that the chosen bandit is truly rewarding (Figure 6a). The beliefs can partly discriminate between truthful and false feedback. These beliefs can partially discriminate between truthful and false feedback. As proof of this, we calculated a Bayesian posterior feedback-truthfulness belief for each participant and trial featuring the 1- or 2-star agents, (Methods; Recall for the 3-star agent, feedback is always true). On testing whether these posterior-truthfulness beliefs vary as a function of objective feedback truthfulness (true vs. lie), we found beliefs are stronger for truthful trials than for untruthful trials for both agents (1-star agent: mean difference = 0.10, *t*(203) = 39.47, p < 0.001; 2-star agent: mean difference = 0.08, *t*(203) = 34.43, p < 0.001) (Figure 6b and Appendix 2—figure 5a). Note that this calculation was feasible because, as experimenters, we had privileged access to the objective truth of the choice feedback as, when designing the experimental sessions, we generated latent true choice outcomes which could be compared to agent-reported feedback.

To formally address whether feedback truthfulness modulates credit assignment, we fitted a new variant of the CA model (the ‘Truth-CA’ model) to the data. This variant works as our credibility-CA model, but incorporated a truth-bonus parameter (*TB*) which increases the degree of credit assignment for feedback as a function of the *experimenter-determined* likelihood the feedback is true (which is read from the curves in Figure 6a when *x* is taken to be the true probability the bandit is rewarding). Specifically, after receiving feedback, the *Q*-value of the chosen option is updated according to the following rule:

$$\displaystyle Q\leftarrow \left (1\mathrm{-}f_{Q}\right)\mathrm{*}Q\mathrm{+}\left [CA\left (agent\right)\mathrm{+}TB\mathrm{*}\left (P\left (truth\right)\mathrm{-}0.5\right)\right ]\mathrm{*}F$$

where $TB$ is the free parameter representing the truth bonus, and $P\left (truth\right)$ is the probability the received feedback being true (from the experimenter’s perspective). We acknowledge that this model falls short of providing a mechanistically plausible description of the credit assignment process, because, participants have no access to the experimenter’s truthfulness likelihoods (as the true bandit reward probabilities are unknown to them). Nonetheless, we use this ‘oracle model’ as a measurement tool to glean rough estimates for the extent to which credit assignment is boosted as a function of its truthfulness likelihood.

Fitting this Truth-CA model to participants' behaviour revealed a significant positive truth-bonus (mean = 0.21, *t*(203) = 3.12, p = 0.002), suggesting that participants indeed assign greater weight to feedback that is likely to be true (Figure 6c; see Appendix 4— Section 3.1 for detailed ML parameter results). Notably, simulations using our other models (Methods) consistently predicted smaller truth biases (compared to the empirical bias) (Figure 6d). Moreover, truth bias was still detected even in a more flexible model that allowed for both a positivity bias and truth-bias (see Appendix 6—Section 3 Truth inference is still detected when controlling for valence bias). The upshot is that participants are biased to assign higher credit based on feedback that is more likely to be true in a manner that is inconsistent with our Bayesian models and above and beyond the previously identified positivity biases.

### Discovery study

The discovery study (*n* = 104) used a disinformation task structurally similar to that used in our main study, but with three notable differences: (1) it included 4 feedback agents, with credibilities of 50%, 70%, 85%, and 100%, represented by 1, 2, 3, and 4 stars, respectively; (2) each experimental block consisted of a single bandit pair, presented over 16 trials (with 4 trials for each feedback agent); and (3) in certain blocks, unbeknownst to participants, the two bandits within a pair were equally rewarding (see Appendix 1−Methods). Overall, this study’s results supported similar conclusions as our main study (see Appendix 1−Results) with a few differences. We found convergent support for increased learning from more credible sources (Appendix 1—Credible feedback promotes greater learning), superior fit for the CA model over Bayesian models (Appendix 1—Substantial deviations from Bayesian Learning) and increased learning from feedback inferred to be true (Appendix 1—True feedback elicits greater learning). Additionally, we found an inflation of positivity bias for low-credibility both when measured relative to the overall level of credit assignment (as in our main study), or in absolute terms (unlike in our main study) (Appendix 1—figure 3; Appendix 1—Individuals show a positivity bias in learning, particularly for sources of limited credibility). Moreover, choice-perseveration could not predict an amplification of positivity bias for low-credibility sources (see Appendix 5—Section 2). However, we found no evidence for learning based on 50%-credibility feedback when examining either the feedback effect on choice-repetition or CA in the credibility-CA model (Appendix 1—The effect of non-credible feedback and learning).

## Discussion

Accurate information enables individuals to adapt effectively to their environment (Wilson et al., 2014; Niv, 2009). Indeed, it has been suggested that the importance and utility of information elevate its status to that of a secondary reinforcer, imbuing it with intrinsic value beyond its immediate usefulness (Bennett et al., 2016; Bromberg-Martin and Monosov, 2020). However, a significant societal challenge arises from the fact that, as social animals, much information we receive is mediated by others, entailing it can be inaccurate, biased or purposefully misleading. Here, using a novel variant of the two-armed bandit task, we asked how we update our beliefs in the presence of potential disinformation, wherein *true choice* outcomes are latent and feedback is provided by potentially disinformative agents.

We acknowledge that several factors may limit the external validity of our task, including the fact that participants were explicitly instructed about the credibility of information sources. In contrast, in many real-life scenarios, individuals need to learn the credibility of information sources based on their own experience of the world or may even have false beliefs regarding the source credibility of agents. Moreover, in our task, the experimenter fully controlled the credibility of the information source in every trial, whereas in many real-life situations people can exercise a degree of control over the credibility of information they receive. For example, search engines allow an exercise of choice regarding the credibility of sources. Finally, in our task, feedback agents served as rudimentary representations of social agents, who lied randomly and arbitrarily, in a motivation-free manner. Conversely, in real life, others may strategically attempt to mislead us, and we can exploit knowledge of their motivation to lie, such as when we assume that a used car seller is more likely to portray a clapped-out car as excellent, rather than state the unfiltered truth. Nevertheless, our results attest to the utility of our task in identifying biased aspects of learning in the face of disinformation, even in a simplified scenario.

Consistent with Bayesian-learning principles, we show that individuals increased their learning as a function of feedback credibility. This aligns with previous studies demonstrating an impressive human ability to flexibly increase learning rates when environmental changes render prior knowledge obsolete (Behrens et al., 2007; Glaze et al., 2015; Glaze et al., 2018), and when there is reduced inherent uncertainty, such as ‘observation noise’ (Behrens et al., 2007; Glaze et al., 2015; Glaze et al., 2018; Behrens et al., 2008). However, as hypothesized, when facing potential disinformation, we also find that individuals exhibit several important biases i.e., deviations from strictly idealized Bayesian strategies. Future studies should explore if and under what assumptions, about the task’s generative structure and/or learner’s priors and objectives, more complex Bayesian models (e.g., active inference; Friston et al., 2017) might account for our empirical findings. In our main study, we show that participants revised their beliefs based on entirely non-credible feedback, whereas an ideal-Bayesian strategy dictates such feedback should be ignored. This finding resonates with the ‘continued-influence effect’ whereby misleading information continues to influence an individual’s beliefs even after it has been retracted (Johnson and Seifert, 1994; Walter and Tukachinsky, 2020). One possible explanation is that some participants failed to infer that feedback from the 1-star agent was statistically void of information content, essentially random (e.g., the group-level credibility of this agent was estimated by our free-credibility Bayesian model as higher than 50%). Participants were instructed that this feedback would be ‘a lie’ 50% of the time but were not explicitly told that this meant it was random and should therefore be disregarded. Notably, however, there was no corresponding evidence random feedback affected behaviour in our discovery study. It is possible that an individual’s ability to filter out random information might have been limited due to a high cognitive load induced by our main study task, which required participants to track the values of three bandit pairs and juggle between three interleaved feedback agents (whereas in our discovery study each experimental block featured a single bandit pair). Future studies should explore more systematically how the ability to filter random feedback depends on cognitive load (Collins and Frank, 2012).

Previous RL studies report greater credit assignment based on positive compared to negative feedback, albeit only in the context of veridical feedback (Lefebvre et al., 2017; Palminteri and Lebreton, 2022; Chambon et al., 2020). Here, we investigated whether a positivity bias is amplified for information of low credibility, but our findings are equivocal and vary as a function of scaling (absolute or relative) and study. We observe selective absolute amplification of a positivity bias for information of low and intermediate credibility in the discovery study alone. In contrast, we find a relative (to the overall extent of CA) amplification of confirmation bias in both studies. Importantly, the magnitude of these amplification effects cannot be reproduced in ex post simulations of a model incorporating simple choice perseveration without an explicit positivity bias, suggesting that at least part of the amplification reflects a genuine increase in positivity bias.

Of note, previous literature has interpreted enhanced learning for positive outcomes in RL as indicative of a confirmation bias (Palminteri et al., 2017; Palminteri and Lebreton, 2022). For example, positive feedback may confirm, to a greater extent than negative feedback one’s choice as superior (e.g., ‘I chose the better of the two options’). Leveraging the framework of motivated cognition (Hughes and Zaki, 2015), we posited that feedback of uncertain veracity (e.g., low credibility) amplifies this bias by incentivising individuals to self-servingly accept positive feedback as true (because it confers positive, desirable outcomes), and explain-away undesirable, choice-disconfirming, negative feedback as false. This could imply an amplified confirmation bias on social media, where content from sources of uncertain credibility, such as unknown or unverified users, is more easily interpreted in a self-serving manner, disproportionately reinforcing existing beliefs (Westerwick et al., 2020). In turn, this could contribute to an exacerbation of the negative social outcomes previously linked to confirmation bias such as polarization (Gallo, 2020; Lefebvre et al., 2024), the formation of ‘echo chambers’ (Modgil et al., 2021), and the persistence of misbelief regarding contemporary issues of importance such as vaccination (Meppelink et al., 2019; Malthouse, 2023) and climate change (Huang and Wang, 2024; Sunstein et al., 2017; Zhou and Shen, 2022; Hart and Nisbet, 2012). We note however, that further studies are required to determine whether positivity bias in our task is indeed a form of confirmation bias. Future studies could also benefit from using designs that are better suited for dissociating learning asymmetries from gradual perseveration (Vidal-Perez et al., 2025b).

A striking finding in our study was that for a fully credible feedback agent, credit assignment was exaggerated (i.e., higher than predicted by our Bayesian models). Furthermore, the effect of fully credible feedback on choice was further boosted when it was preceded by a low-credibility context related to current learning. We interpret this in terms of a ‘contrast effect’, whereby veridical information looms larger against a backdrop of disinformation (Pennycook et al., 2020). One upshot is that exaggerated learning might entail a risk of jumping to premature conclusions based on limited credible evidence (e.g., a strong conclusion that a vaccine produces significant side-effect risks based on weak credible information, following non-credible information about the same vaccine). An intriguing possibility, that could be tested in future studies, is that participants strategically amplify the extent of learning from credible feedback to dilute the impact of learning from non-credible feedback. For example, a person scrolling through a social media feed, encountering copious amounts of disinformation, might amplify the weight they assign to credible feedback in order to dilute effects of ‘fake news’. Ironically, these results also suggest that public campaigns might be more effective when embedding their messages in low-credibility contexts, which may boost their impact.

Our findings show that individuals increase their credit assignment for feedback in proportion to the perceived probability that the feedback is true, even after controlling for source credibility and feedback valence. Strikingly, this learning bias was not predicted by any of our Bayesian or credit assignment (CA) models. Notably, our evidence for this bias is based on a ‘oracle model’ that incorporates the probability of feedback truthfulness from the experimenter’s perspective, rather than the participant’s. This raises an important open question: how do individuals form beliefs about feedback truthfulness, and how do these beliefs influence credit assignment? Future research should address this by eliciting trial-by-trial beliefs about feedback truthfulness. Doing so would also allow for testing the intriguing possibility that an exaggerated positivity bias for non-credible sources reflects, to some extent, a truth-based discounting of negative feedback—i.e., participants may judge such feedback as less likely to be true. However, it is important to note that the positivity bias observed for fully credible sources (here and in other literature) cannot be attributed to a truth bias—unless participants were, against instructions, distrustful of that source.

An important question arises as to the psychological locus of the biases we uncovered. Because we were interested in how individuals process disinformation—deliberately false or misleading information intended to deceive or manipulate—we framed the feedback agents in our study as deceptive, who would occasionally ‘lie’ about the true choice outcome. However, statistically (though not necessarily psychologically), these agents are equivalent to agents who mix truth-telling with random ‘guessing’ or ‘noise’ where inaccuracies may arise from factors such as occasionally lacking access to true outcomes, simple laziness, or mistakes, rather than an intent to deceive. This raises the question of whether the biases we observed are driven by the perception of potential disinformation as deceitful per se or simply as deviating from the truth. Future studies could address this question by directly comparing learning from statistically equivalent sources framed as either lying or noisy. Unlike previous studies wherein participants had to infer source credibility from experience (Vélez and Gweon, 2019; Schulz et al., 2023; Diaconescu et al., 2014), we took an explicit-instruction approach, allowing us to precisely assess source credibility impact on learning, without confounding it with errors in learning about the sources themselves. More broadly, our work connects with prior research on observational learning, which examined how individuals learn from the actions or advice of social partners (Diaconescu et al., 2014; Zhang and Gläscher, 2020; Burke et al., 2010; Chelarescu, 2021). This body of work has demonstrated that individuals integrate learning from their private experiences with learning based on others’ actions or advice—whether by inferring the value others attribute to different options or by mimicking their behaviour (Behrens et al., 2008; Charpentier et al., 2020). However, our task differs significantly from traditional observational learning. Firstly, our feedback agents interpret outcomes rather than demonstrating or recommending actions (Vélez and Gweon, 2019; Schulz et al., 2023; Diaconescu et al., 2014). Secondly, participants in our study lack private experiences unmediated by feedback sources. Finally, unlike most observational learning paradigms, we systematically address scenarios with deliberately misleading social partners. Future studies could bridge this by incorporating deceptive social partners into observational learning, offering a chance to develop unified models of how individuals integrate social information when credibility is paramount for decision-making.

We conclude by noting previous research has often attributed the negative impacts of disinformation, such as polarization and the formation of echo chambers, to intricate processes facilitated by external or self-selection of information (Garrett, 2009; Ross Arguedas et al., 2022; Cardenal et al., 2019). These processes include algorithms tailoring information to align with users’ attitudes (Brady et al., 2023) or individuals consciously opting to engage with like-minded peers (Bakshy et al., 2015). However, our study reveals a more profound effect of disinformation, namely that even in minimal conditions, when low-credibility information is explicitly identified, disinformation significantly impacts individuals' beliefs and decision-making processes. This occurs even when the decision at hand entails minimal emotional engagement or pertinence to deep, identity-related issues. A critical next step is to deepen our understanding of these biases, particularly within complex social environments, not least to enable the development of effective prospective interventions capable of mitigating the potentially pernicious impacts of disinformation.

## Materials and methods

### Participants

We recruited 246 participants (mean age 39.33 ± 12.65, 112 female) from the Prolific participant pool (https://www.prolific.co) who went on to perform the task on the Gorilla platform (Anwyl-Irvine et al., 2020). All participants were fluent English speakers with normal or corrected-to-normal vision and a Prolific approval rate of 95% or higher. UCL Research Ethics Committee approved the study (Project ID 6649/004), and all participants provided prior informed consent.

### Experimental protocol

#### Traditional two-armed bandit task

At the beginning of the experiment participants completed a traditional version of the two-armed bandit task. Participants performed 45 trials, each featuring one of three randomly interleaved bandit pairs (such that each pair was presented on 15 trials). On each trial, participants chose between the bandit pair, with each bandit being represented by a distinct identicon. Once a bandit was selected it generated a true outcome (converted to bonus monetary compensation) corresponding to either a reward or nothing. Within each bandit pair, one bandit provided rewards on 75% of trials (with 25% providing no-reward), while the other bandit rewarded on 25% of the trials (75% non-reward trials). Participants were uninformed about the reward probabilities of each bandit and had to learn these based on experience.

At onset of each trial, the two bandits were presented, one on each side of the screen, and participants were asked to indicate their choice within 3 s by pressing the left/right arrow keys. If the 3 s elapsed with no choice, participants were shown a ‘too slow’ message and proceeded to the next trial. Following choice, the unselected bandit disappeared, and the participants were presented with the outcome of the selected bandit for 1200ms, followed by a 250-ms ISI before the start of the next trial. Rewards were represented by a green dollar symbol and non-rewards by a red sad face (both in the centre of the screen). At the end of the task, participants were informed about the number of rewards they had earned.

#### Disinformation task

This involved a modified, disinformation version of the same two-armed bandit task. Participants performed 8 blocks, each consisting of 45 trials. Each block followed the structure of the traditional two-armed bandit task, but with a critical difference: true choice outcomes were withheld from participants and instead they received reward feedback from a feedback agent. Participants were instructed prior to the task that feedback agents mostly provide accurate feedback (i.e., the true outcome) but could lie on a random minority of trials by reporting a reward in case of a true non-reward, or vice versa. The task featured three feedback agents varying in their credibility (i.e., probability of truth-telling), as indicated by a ‘star-rating’ system, about which participants were instructed prior to the task. The 3-star agent always told the truth, whereas the other two agents were partially credible, reporting the truth on 75% (2-star) or 50% (1-star) of the trials. Feedback agents were randomly interleaved across trials subject to the constraint that each agent appeared on five trials for each bandit pair.

At the onset of each trial, participants were presented with the feedback agent for the trial (screen centre) and with the two bandits, one on each side of the screen. Participants made a 2-s time limited choice by pressing the left/right arrow keys. Following choice, the unselected bandit disappeared, and were then presented with the agent feedback for 1200 ms (represented by either a rewarding green dollar sign or a non-rewarding red sad face in the centre of the screen). All stimuli then disappeared for 250 ms to be followed by the start of the next trial. At the end of each block, participants were informed about the number of true rewards they had earned. They then received a 30-s break before the next block started with new three bandit pairs.

#### General protocol

At the beginning of the experiment, participants were presented with instructions for the traditional two-armed bandit task. The instructions were interleaved with four multiple-choice questions. When participants answered a question incorrectly, they could re-read the instructions and re-attempt. If participants answered a question incorrectly twice, they were compensated for the time but could not continue to the next stage. Upon completing the instructions participants proceeded to the traditional two-armed bandit task.

After the two-armed bandit task, participants were presented with instructions regarding the disinformation task. Again, these were interleaved with six questions wherein participants had two attempts to answer each question correctly. If they answered a question incorrectly twice, they were rejected and received partial participatory compensation. Participants then proceeded to the disinformation task. After completing the disinformation task, participants completed three psychiatric questionnaires (presented in random order): (1) the Obsessional Compulsive Inventory—Revised (OCI-R) (Foa et al., 2002), assessing symptoms of obsessive–compulsive disorder (OCD); (2) the Revised Green et al. Paranoid Thoughts Scale (R-GPTS) (Freeman et al., 2021), measuring paranoid ideations; and (3) the DOG scale, evaluating dogmatism (Altemeyer, 2002).

The participants took on average 43 min to complete the experiment. They received a fixed compensation of 5.48 GBP and variable compensation between 0 and 2 GBP based on their performance on the disinformation task.

#### Attention checks

The two tasks included randomly interleaved catch trials wherein participants were cued to press a given key within a 3-s limit. None of the participants failed more than one of these attention checks.

### Data analysis

#### Exclusion criteria

Participants were excluded if they: (1) either repeated or alternated key presses in more than 70% of the trials, and/or (2) their reaction time was lower than 150 ms in more than 5% of the trials. Based on these criteria, 42 participants were excluded while 204 participants were kept for the analyses.

#### Accuracy

Accuracy rates were calculated as the probability of choosing within a given pair the bandit with a higher reward probability. For Figure 1d, we calculated for each participant and for each trial (within a bandit pair) averaged accuracy across all bandit pairs. We then averaged accuracy at the trial level across participants. Overall improvement for each participant was calculated as the average accuracy difference between the last and first trials for each of the bandit pairs.

### Computational models

#### RL models

We formulated a family of RL models to account for participant choices. In these models, a tendency to choose each bandit is captured by a *Q*-value. After reward-feedback the *Q*-value of the chosen bandit was updated conditional on the agent and on whether the feedback was positive or negative according to the following rule:

(1) $$\displaystyle Q\left (chosen\right)\leftarrow \left (1{-}f_{Q}\right)\mathrm{*}Q\left (chosen\right){+}CA\left (agent,valence\right){*}F$$

where *CA* is a free credit assignment parameter representing the magnitude of the value increase/decrease following feedback receipt *F* from the agents (coded as 1 for reward feedback and –1 for non-reward feedback), while *f_Q_* (∈[0,1]) is the free parameter representing the forgetting rate of the *Q*-value. Additionally, the value of each of the other bandits (i.e., the unchosen bandit in the presented pair and all the bandits from the other not-shown pairs) was forgotten as per the following:

(2) $$\displaystyle Q\left (non-chosen\right)\leftarrow \left (1-f_{Q}\right)\mathrm{*}Q\left (non{-}chosen\right)$$

Alternative model variants differed based on whether the CA parameter(s) were influenced by agents and/or feedback valence (see Table 1 below), allowing us to test how these variables impacted learning.

**Table 1.** Summary of free parameters for each of the CA models.

Model	Free CA parameter
Null	*CA*
Credibility-CA	$CA_{0.5},CA_{0.75},CA_{1}$
Credibility-valence-CA	$CA_{0.5}\mathrm{+},CA_{0.75}\mathrm{+},CA_{1}\mathrm{+}$
	$CA_{0.5}\mathrm{-},CA_{0.75}\mathrm{-},CA_{1}\mathrm{-}$
Constant feedback-valence bias CA	*VB*
	$CA_{0.5}\mathrm{-},CA_{0.75}\mathrm{-},CA_{1}\mathrm{-}$
Truth-CA	*TB*
	$CA_{0.5},CA_{0.75},CA_{1}$

1. The ‘Null’ model included a unique CA parameter conveying an assumption that feedback is modulated by neither agent-credibility nor feedback valence.
2. The ‘credibility-CA’ models included a dedicated CA parameter for each agent allowing for the possibility learning was selectively modulated by agent-credibility (but not by feedback valence).
3. The ‘credibility-valence-CA’ model included distinct CA parameters for rewarding (CA^+^) and non-rewarding feedback (CA^−^) for each agent, allowing CA to be influenced by both feedback valence and credibility.
4. The ‘constant feedback-valence bias’ CA model included separate CA^−^ parameters for each agent, but a single valence bias parameter (VB) common to all agents, such that the CA^+^ parameter for each agent corresponded to the sum of its CA^−^ parameter and the common VB parameter.

Additionally, we formulated a ‘Truth-CA’ model, which worked as our credibility-CA model, but incorporated a free truth-bonus parameter (*TB*). This parameter modulates the extent of credit assignment for each agent based on the posterior probability of feedback being true (given the credibility of the feedback agent, and the true reward probability of the chosen bandit). The chosen bandit was updated as follows:

(3) $$\displaystyle Q\leftarrow \left (1-f_{Q}\right)*Q\mathrm{+}\left [CA\left (agent\right)+TB*\left (Prob\left (truth\right){-}0.5\right)\right ]{*}F$$

where Prob(truth) is the posterior probability of the feedback being true in the current trial (for exact calculation of Prob(truth) see ‘Methods: Bayesian estimation of posterior belief that feedback is true’).

All models also included gradual perseveration for each bandit. In each trial the perseveration values (Pers) were updated according to

(4) $$\displaystyle Pers\left (chosen\right)\leftarrow \left (1{-}f_{P}\right){*}Pers\left (chosen\right){+}PERS$$

where PERS is a free parameter representing the Pers-value change for the chosen bandit, and *f_P_* (∈[0,1]) is the free parameter denoting the forgetting rate applied to the Pers value. Additionally, the Pers-values of all the non-chosen bandits (i.e., again, the unchosen bandit of the current pair, and all the bandits from the not-shown pairs) were forgotten as follows:

(5) $$\displaystyle Pers\left (non{-}chosen\right)\leftarrow \left (1{-}f_{P}\right){*}Pers\left (non{-}chosen\right)$$

We modelled choices using a *softmax* decision rule, representing the probability of the participant to choose a given bandit over the alternative:

(6) $$\displaystyle Prob\left (bandit\right)=\frac{1}{1\mathrm{+}e^{\left [Q\left (other\, bandit\right)-Q\left (bandit\right)\right ]+\left [Pers\left (other\, bandit\right)-Pers\left (bandit\right)\right ]}}.$$

#### Bayesian models

We also formulated a Bayesian model corresponding to an ideal belief updating strategy. In this model, beliefs about each bandit were represented by a density distribution over the probability that a bandit provides a true reward *g*(*p*)*,* where *p* is the probability of a true reward (see full derivation in Appendix 7) During learning, following reward-feedback, the distribution *for* the chosen bandit was updated based on the agent’s feedback (*F*) and its associated credibility (*C*):

(7) $$\displaystyle g\left (p\right)\leftarrow g\left (p\right)*\left [C*p+\left (1-C\right)*\left (1-p\right)\right ]\,if\,F=1$$

(8) $$\displaystyle g\left (p\right)\leftarrow g\left (p\right)*\left [\left (1-C\right)\,*p+C*\left (1-p\right)\right ]if\, F=-1$$

(9) $$\displaystyle g\left (p\right)\leftarrow \frac{g\left (p\right)}{\int _{0}^{1}g\left (p\right)\, dp}$$

At the beginning of each block priors for each bandit were initialized to uniform distributions (*g*(*p*) = *U*[0,1]). In the *instructed-credibility Bayesian model*, we fixed the credibilities to their true values (i.e., 0.5, 0.75, and 1).

We also formulated a *free-credibility Bayesian model*, where we only fixed the 3-star agent-credibility to 1 but estimated the credibility of the two lying agents as free parameters. This model allowed the possibility that participants use distorted instructed-credibilities when following a Bayesian strategy.

For both versions, we modelled choice using a SoftMax function with a free inverse temperature parameter ($\beta $):

(10) $$\displaystyle Prob\left (bandit\right)=\frac{1}{1{+}e^{\beta *\left [Q\left (other\, bandit\right)-Q\left (bandit\right)\right ]}}$$

where here *Q*(*bandit*) is the expected probability, the bandit provides a true reward.

Additionally, we formulated extended Bayesian models to account for choice-perseveration (see Appendix 6—Section 1). These models operate as our instructed- and free-credibility Bayesian models, but also incorporate a perseveration value, updated in each trial as in our CA models (Equations 4; 5). For these extended models, we modelled choices using the following *softmax* decision rule:

(11) $$\displaystyle Prob\left (bandit\right){=}\frac{1}{1{+}e^{\beta \mathrm{*}\left [Q\left (other\, bandit\right){-}Q\left (bandit\right)\right ]{+}\left [Pers\left (other\, bandit\right){-}Pers\left (bandit\right)\right ]}}$$

### Parameter optimization, model selection, and synthetic model simulations

For each participant, we estimated the free parameter values that maximized the summed log-likelihood of the observed choices across all games. Trials where participants showed a response time below 150 ms were excluded from the log-likelihood calculations. To minimize the chances of finding local minima, we ran the fitting procedure 10 times for each participant, using random initializations for the parameters (CA ~ *U*[–10,10], PERS ~ *U*[–5,5], *f*_*Q*_ ~ [0,1], *f*_*P*_ ~ [0,1], TB ~ [–10,10], *β* ~ [0,30], *C* ~ *U*[0,1]).

We performed model comparison between Bayesian and CA models using the PBCM (Wagenmakers et al., 2004; Moran et al., 2019). In brief, this method relies on generating, for each participant, synthetic datasets (we used 201) based on maximal likelihood parameters and each model variant (i.e., the Bayesian model and the CA model), and fitting each dataset with the two models. We then calculated the log likelihood difference between the two fits for each dataset, obtaining two log-likelihood difference distributions, one for each generative model. We determined a loglikelihood difference threshold that leads to best model classification (i.e., maximizing the proportion of true positives and true negatives). Finally, we fit the empirical data from each participant with the two model variants, calculating an empirical loglikelihood difference. A comparison of this empirical likelihood difference to the classification threshold determines which model provides a better fit for a participant’s data (see Appendix 2—figure 2 for more information). We used this procedure to compare our Bayesian models (instructed-credibility and free-credibility Bayesian) with a simplified version of the credibility-CA model that did not include perseveration (PERS, fP = 0).

We also performed model comparisons for nested CA models using generalized-likelihood ratio tests where the null distribution for rejecting a nested model (in favour of a nesting model) was based on a bootstrapping method (BGLRT) (Moran et al., 2021; Moran and Goshen-Gottstein, 2015).

To assess the mechanistic predictions of each model, we generated synthetic simulations based on the ML parameters of participants. Unless stated otherwise, we generated 5 simulations for each participant (1020 total simulations) with a new sequence of trials generated as in the actual data. We analysed these data in the same way as we analysed empirical data, after pooling together the 5 simulated datasets per participant.

### Parameter recovery

For each model of interest, we generated 201 synthetic simulations based on parameters sampled from uniform distributions (CA ~ *U*[–10,10], PERS ~ *U*[–5,5], *f*_*Q*_ ~ *U*[0,1], *f*_*P*_ ~ *U*[0,1], *β* ~ *U*[0,30], *C* ~U[0,1]). We fitted each simulated dataset with its generative model and calculated the Spearman’s correlation between the generative and fitted parameters.

### Mixed-effects models

#### Model-agnostic analysis of agent-credibility effects on choice-repetition

We used a mixed-effects binomial regression model to assess whether, and how, value-learning was modulated by agent-credibility, with participants serving as random effects. The regressed variable *REPEAT* indicated whether the current trial repeated the choice from the previous trial featuring the same bandit pair (repeated choice = 1, non-repeated choice = 0) and was regressed on the following regressors: *FEEDBACK* coded whether feedback received in the previous trial with the same bandit pair was positive or negative (coded as 0.5 and–0.5, respectively), *BETTER* coded whether the bandit chosen in that previous trial was the better—mostly rewarding—or the worse—mostly unrewarding—bandit within the pair, coded as 0.5 and –0.5, respectively, AGENT_2-star_ indicated whether feedback received in the previous trial (featuring the same bandit pair) came from the 2-star agent (previous feedback from 2-star agent = 1, otherwise = 0) and AGENT_3-star_ indicated whether the feedback in the previous trial came from the 3-star agent. The model in Wilkinson’s notation was:

(12) $$\displaystyle REPEAT{\sim }FEEDBACK*BETTER*\left (AGENT_{2-star}{+}AGENT_{3-star}\right){+}\left (1|participant\right)$$

In Figure 2a, b, we plot the choice-repeat probability based on feedback-valence and agent-credibility from the preceding trial with the same bandit pair. We independently calculated the repeat probability for the better (mostly rewarding) and worse (mostly non-rewarding) bandits and averaged across them. This calculation was done at the participants level, and finally averaged across participants.

#### Model-agnostic analysis of contextual credibility effects on choice-repetition

We used a different mixed-effects binomial regression model to test whether value learning from the 3-star agent was modulated by contextual credibility. We focused this analysis on instances where the previous trial with the same bandit pair featured the 3-star agent. We regressed the variable *REPEAT*, which indicated whether the current trial repeated the choice from the previous trial featuring the same bandit pair (repeated choice = 1, non-repeated choice = 0). We included the following regressors: *FEEDBACK* coding the valence of feedback in the previous trial with the same bandit pair (positive = 0.5, negative = −0.5), CONTEXT_2-star_ indicating whether the trial immediately preceding the previous trial with the same bandit pair (context trial) featured the 2-star agent (feedback from 2-star agent = 1, otherwise = 0), and CONTEXT_3-star_ indicating whether the trial immediately preceding the previous trial with the same bandit pair featured the 3-star agent. We also included a regressor (*BETTER*) coding whether the bandit chosen in the learning trial was the better—mostly rewarding—or the worse—mostly unrewarding—bandit within the pair. We included in this analysis only current trials where the context trial featured the same bandit pair. The model in Wilkinson’s notation was:

(13) $$\displaystyle REPEAT{\sim }FEEDBACK{*}\left (CONTEXT_{2-star}{+}CONTEXT_{3-star}\right){+}BETTER{+}\left (1|participant\right)$$

In Figure 4c, we independently calculate the repeat probability difference for the better (mostly rewarding) and worse (mostly non-rewarding) bandits and averaged across them. This calculation was done at the participants level, and finally averaged across participants.

#### Effects of agent-credibility on CA parameters from credibility-CA model

We used a mixed-effects linear regression model to assess whether, and how, credit assignment was modulated by feedback agent, with participants serving as random effects (data from Figure 2c). We regressed the maximal likelihood CA parameters from the credibility-CA model. The regressors AGENT_2-star_ and AGENT_3-star_ indicated, respectively, whether the CA parameter was attributed to the 2-star or the 3-star agent. The model’s Wilkinson’s notation was:

(14) $$\displaystyle CA{\sim }AGENT_{2-star}{+}AGENT_{3-star}+\left (1{|}participant\right)$$

#### Effects of agent-credibility and feedback valence on CA parameters from credibility-valence-CA model

We used a second mixed-effects linear regression model to test for a valence bias in learning, and how such bias was modulated by feedback credibility, with participants serving again as random effects (data from Figure 3a). The maximal likelihood CA parameters from the credibility-valence-CA model served as the regressed variable, which was regressed on: AGENT_2-star_ and AGENT_3-star_ (defined in the same way as the previous model), and *VALENCE* coding whether the CA parameter was attributed to positive (coded as 0.5) or negative (coded as –0.5) feedback. The Wilkinson’s notation of the model was:

(15) $$\displaystyle CA{\sim }VALENCE{*}\left (AGENT_{2-star}+AGENT_{3-star}\right)+\left (1|participant\right)$$

We used a separate mixed-effects linear regression model to test how relative valence bias was modulated by feedback credibility. We first computed the relative valence bias index (rVBI) for each credibility level, and we then regressed these values on AGENT_2-star_ and AGENT_3-star_ (defined in the same way as the previous models).

(16) $$\displaystyle rVBI=\frac{CA^{+}-CA^{-}}{|CA^{+}|{+}|CA^{-}|}$$

$$\displaystyle rVBI{\sim }AGENT_{2-star}{+}AGENT_{3-star}{+}\left (1|participant\right)$$

### Bayesian estimation of posterior belief that feedback is true

We calculated the Bayesian posterior conditional probability of feedback truthfulness (Figure 4a, b) follows. First, we calculated the probability of each true outcome, *r* (0: non-reward; 1: reward) conditional on the feedback, *f* (0: non-reward, 1: reward), the credibility of the agent reporting the feedback (*C*) and the history of experiences from past trials (*H*):

(17) $$\displaystyle \begin{array}{ll}Prob\left (r|f,\,C,\,H\right)\propto Prob\left (f|r,C,H\right){*}Prob\left (r|C,H\right){=}Prob\left (f|r,C\right){*}p\left (r|H\right){=}\\\left [C{*}1_{f{=}r}\mathrm{+}\left (1{-}C\right){*}1_{f\neq r}\right ]{*}\left [\bar{p}*1_{r=1}{+}\left (1-\bar{p}\right){*}1_{r{=}0}\right ]\end{array}$$

where proportionality omits terms independent of *r*, $\bar{p}{=}\int _{0}^{1}pg\left (p{|}H\right)dp$ is the expected probability of the chosen bandit being rewarding (conditional on past-trial history), and $g\left (p\mathrm{|}H\right)$ is the density over the probability (the chosen bandit) being rewarded (conditional on the history of previous trials).

Next, we normalized the two terms (for *r* = 0,1) to sum to 1 (to correct for the proportionality Walter and Murphy, 2018). Finally, the posterior belief in truthfulness was taken as *Prob*(*r = f* | *f*,*C*,*H*).

In Figure 4b, we calculated for each participant the mean posterior belief of truthfulness separately for trials where each agent told the truth or lied, and we compared these mean beliefs between the two kinds of trials using paired *t*-tests (one test per agent).

## Figures and Tables

**Appendix 1—figure 1.**
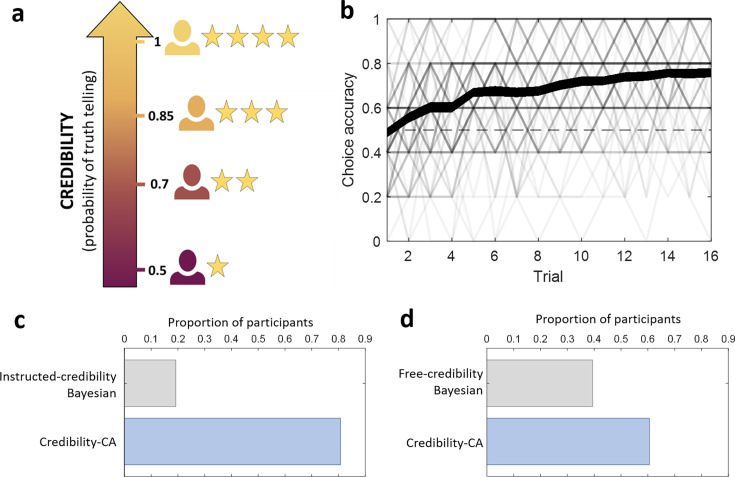
Task design, performance, and model selection in discovery study. (**a**) During the task participants received feedback from four feedback agents, varying in their credibility (i.e., truth-telling probability). The credibility of the agents was represented using a star-based system: the 4-star agent always reported the truth (and never lied), whereas the 3-star agent reported the truth on 85% of the trials (lying on the remaining 15%), the 2-star agent reported the truth on 70% of the trials (lying on the remaining 30%), and the 1-star agent reported the truth half of the time (lying on the other half). Participants were explicitly instructed and quizzed about the credibility of each agent prior to the task. (**b**) Learning curve. Average choice accuracy as a function of trial number (within a bandit pair). Thin lines: individual participants; thick line: group mean with thickness representing the group standard error of the mean for each trial. (**c, d**) Model selection between the credibility-CA model and the two variants of Bayesian models. Most participants were best fitted by the credibility-CA model, compared to the instructed-credibility Bayesian (**c**) or free-credibility Bayesian (**d**) models.

**Appendix 1—figure 2.**
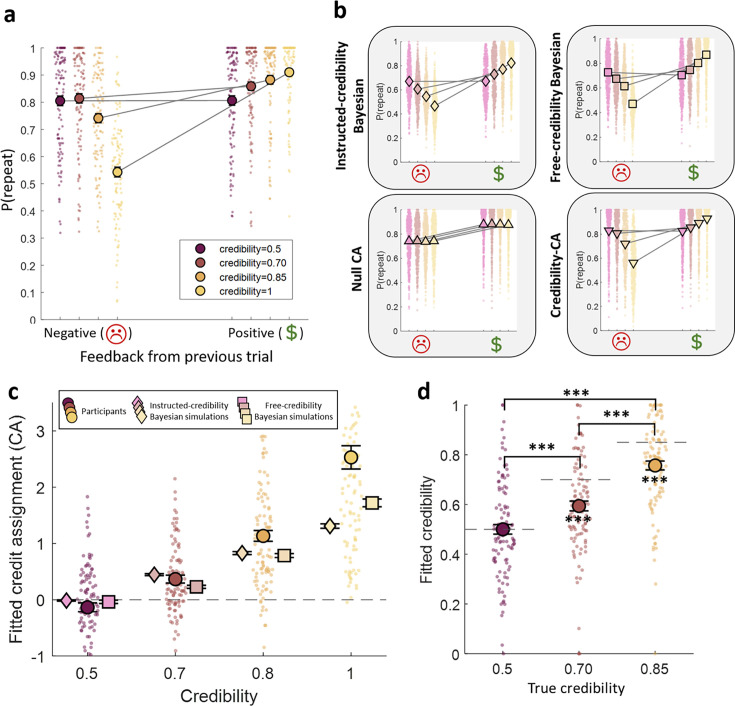
Learning adaptations to credibility in discovery study. (**a**) Probability of repeating a choice as a function of feedback-valence and agent-credibility on the previous trial with the same bandit pair. As in the main study, the effect of feedback-valence on repetition increases as feedback credibility increases, indicating that more credible feedback has a greater effect on behaviour. (**b**) Same analysis as in panel a, but for synthetic data obtained by simulating the main models. Simulations were computed using the ML parameters of participants for each model. (**c**) ML credit assignment parameters for the credibility-CA model. Consistent with the main study, participants show a CA increase as a function of agent-credibility, as predicted by cross-fitting parameters from instructed-credibility Bayesian and free-credibility Bayesian model simulations. However, we find no evidence for a positive CA for the 1-star agent. (**d**) ML credibility parameters for a free-credibility Bayesian model attributing credibility 1 to the 4-star agent but estimating credibility for the three lying agents as free parameters. Small dots represent results for individual participants/simulations, big circles represent the group mean (**a, b, d**) or median (**c**) of participants’ behaviour. Results of the synthetic model simulations are represented by diamonds (instructed-credibility Bayesian model), squares (free-credibility Bayesian model), upward-pointing triangles (null-CA model) and downward-pointing triangles (credibility-CA model). Error bars show the standard error of the mean. *p < 0.05, **p < 0.01, ***p < 0.001.

**Appendix 1—figure 3.**
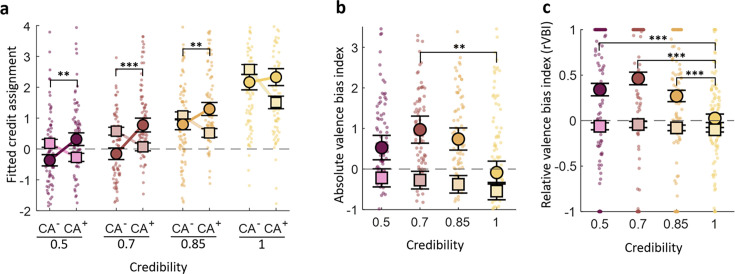
Positivity bias as a function of agent-credibility in the discovery study. (**a**) Maximum likelihood parameters from the credibility-valence-CA model. CA^+^ and CA^−^ are free parameters representing credit assignments for positive and negative feedback, respectively (for each credibility level). The data revealed a positivity bias (CA^+^ > CA^−^) for feedback of low-credibility, but not for fully credible feedback. (**b**) Absolute valence bias index (defined as CA^+^ − CA^−^) based on the ML parameters from the credibility-valence CA model. Positive values indicate a positivity bias, while negative values represent a negativity bias. (**c**) Relative valence bias index (rVBI, defined as (CA^+^ − CA^−^)/(|CA^+^| + |CA^−^|)) based on the ML parameters from the credibility-valence CA model. Positive values indicate a positivity bias, while negative values represent a negativity bias. Small dots represent fitted parameters for individual participants and big circles represent the group median (**a, b**) or mean (**c**) (both of participants’ behaviour), while squares are the median or mean of the fitted parameters of the free-credibility Bayesian model simulations. Error bars show the standard error of the mean. **p < 0.01, ***p < 0.001 for ML fits of participants' behaviour.

**Appendix 1—figure 4.**
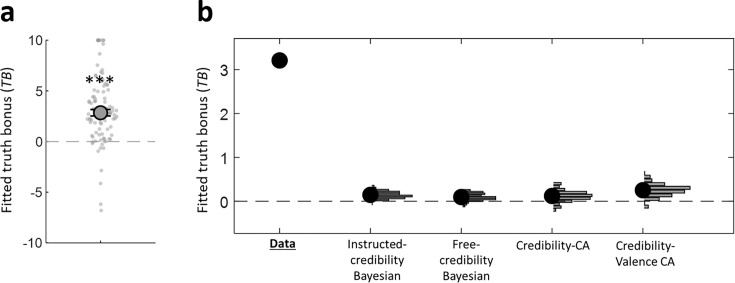
Credit assignment is enhanced for feedback inferred to be true in discovery study. (**a**) Maximum likelihood (ML) estimate of the "truth-bonus" parameter derived from the ‘Truth-CA’ model. The significantly positive truth bonus indicates that participants increased the degree to which they updated their value estimates (credit assignment) when they inferred a higher probability that the feedback they received was true. Each small dot represents the fitted truth-bonus parameter for an individual participant, the large circle indicates the group mean, and the error bars represent the standard error of the mean. (**b**) Distribution of truth-bonus parameters predicted by synthetic simulations of our alternative computational models. For each alternative model, we generated 101 group-level synthetic datasets based on the maximum likelihood parameters fitted to the participants' actual behaviour. Each of these synthetic datasets was then independently fitted with the ‘Truth-CA’ model. Each histogram represents the distribution of the mean truth bonus across the 101 simulated datasets for a specific alternative model. Notably, the truth bonus observed in our participants was significantly higher than the truth bonus predicted by any of these alternative models (proportion of datasets predicting a higher truth bonus = 0 for all models). ***p < 0.001

**Appendix 2—figure 1.**
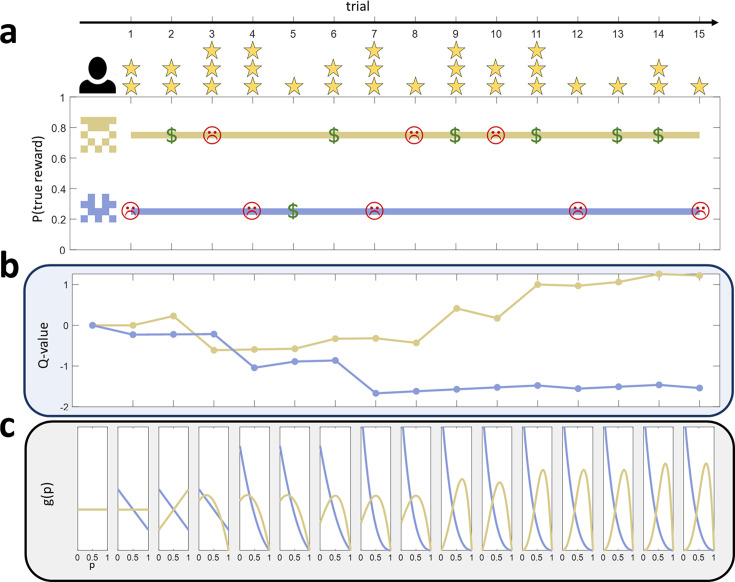
Illustration of computational models over 15 trials with an example bandit pair. (**a**) Example block with a bandit pair. The top bandit provided true rewards 75% of the time, while the bottom bandit did so 25% of the time. The agent providing feedback on each trial is represented above the plot, while the feedback is depicted in the horizontal line of the bandit selected for that trial. Dollar signs represent positive feedback, while sad emojis represent negative feedback. (**b**) Values of the two bandits computed based on credibility-CA model. The values are represented as point estimates (i.e., *Q*-values). On each trial, the *Q*-value of the selected bandit is updated based on the feedback valence and credibility. Values correspond to ends of trials following credit assignment. (**c**) Posterior beliefs about the true reward probabilities of the bandits, computed using the instructed-credibility Bayesian model. The *x*-axis in each subplot represents the probability of a true reward (p), while the *y*-axis represents the density distribution of such true reward probability (*g*(*p*)). On each trial, the density distribution *g*(*p*) of the selected bandit is updated based on the feedback valence and credibility. Both (**b**) and (**c**) were generated with the mean ML parameters from participants fitted with the CA-credibility and instructed-credibility Bayesian models, respectively.

**Appendix 2—figure 2.**
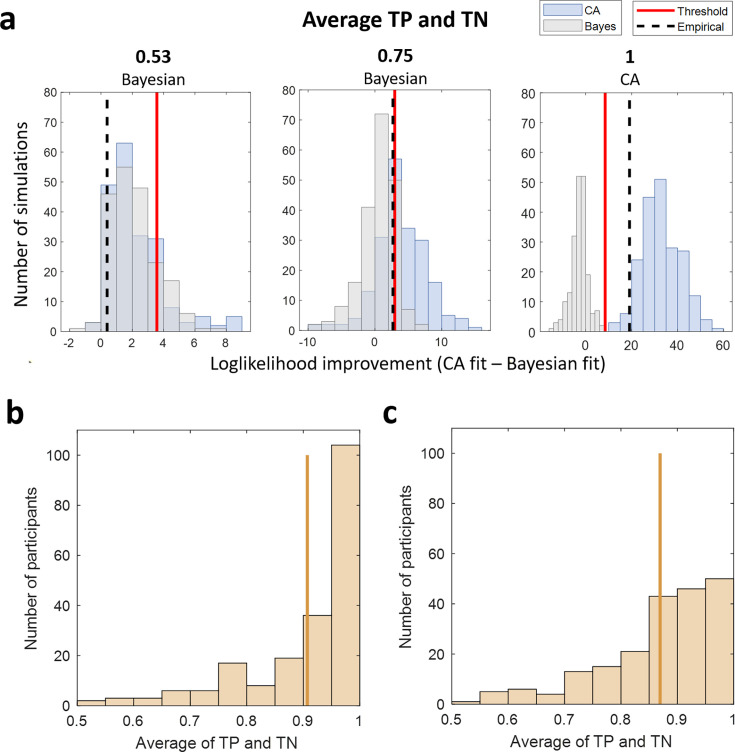
Model comparison between Bayesian models and credibility-CA model for main study. (**a**) Histograms of log-likelihood improvements for three example participants. For each participant, we generated 201 simulations based on their ML parameters for each model variant (i.e., the Bayesian model and the CA model). We fitted each dataset with the two models and calculated the log-likelihood difference between the two fits for each dataset (CA fit – Bayesian fit), resulting in two log-likelihood difference distributions: one for the dataset based on Bayesian simulations (grey) and another for the dataset based on CA simulations (blue). A greater value along the *x*-axis indicates that a dataset was better fitted with the CA model compared to the Bayesian model. We determined a log-likelihood difference threshold that leads to the best model classification (i.e., maximizing the average of true positives and true negatives), represented by a red line in the plots. Finally, we fitted the empirical data of each participant with the two model variants, calculating an empirical log-likelihood difference, represented as a black dashed line in the plots. The three example plots correspond to participants with different model classification accuracy (i.e., proportion of true positives and true negatives) and different model classifications, both stated above each subplot. (**b**) Distribution of model classification accuracy for the model comparison between the instructed-credibility Bayesian and credibility-CA models. A greater average of TP and TN represents a better discrimination between the models. (**c**) Distribution of model classification accuracy for the model comparison between the free-credibility Bayesian and credibility-CA models. Vertical orange lines represent the mean classification accuracy for each model comparison.

**Appendix 2—figure 3.**
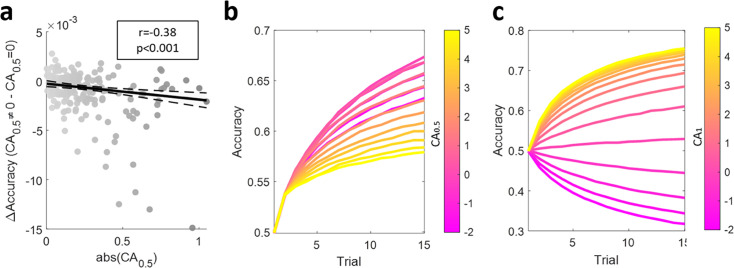
Effects of CA for the 1-star and 3-star agents on accuracy. (**a**) Scatter plot between the absolute CA parameter for the 1-star agent in the main task (*x*-axes) and the predicted drop in accuracy due to CA based on random feedback (*y*-axes). For each participant, we generated 5000 synthetic simulations based on their ML parameters from the credibility-CA model, and another 5000 simulations using the same ML parameters but ablating CA for the 1-star agent by fixing it to 0. The difference in mean accuracy between the two datasets represents the estimated drop in accuracy for each participant due to learning from random feedback. The negative Pearson’s correlation illustrates that accuracy drop increases as a function of (absolute) credit assignment based on random feedback. Circles represent the datapoints of individual participants, lines represent the prediction from linear regression on the data with shaded areas representing the 99% confidence interval. (**b**) Mean learning curves for different 1-star CA parameter values. We generated synthetic simulations based on the credibility-CA model ML parameters from participants but fixing the 1-star CA to different values between –2 and 5. We generated 100 synthetic simulations per participant for each 1-star CA value and averaged across participants. As (absolute) CA attributed to random feedback diverges from 0 (with all other parameters held invariant), accuracy decreases. (**c**) For comparison with b we show the mean learning curves for different 3-star CA values, calculated in the same way but fixing the 3-star CA to different values. Here, accuracy increases with the CA attributed to fully credible.

**Appendix 2—figure 4.**
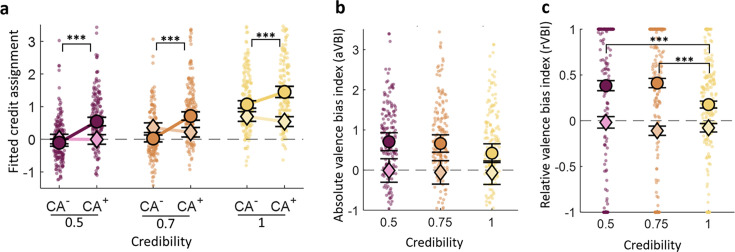
Positivity bias as a function of agent-credibility compared with instructed-credibility Bayesian-CA parameters in the main study. (**a**) ML parameters from the credibility-valence-CA model. CA^+^ and CA^−^ are free parameters representing credit assignments for positive and negative feedback, respectively (for each credibility level). Empirical-CA parameters revealed a positivity bias (CA^+^ > CA^−^) for all credibility levels, while instructed-credibility Bayesian-CA parameters revealed a negativity bias. (**b**) Absolute valence bias index (defined as CA^+^ − CA^−^) based on the ML parameters from the credibility-valence CA model. Positive values indicate a positivity bias, while negative values represent a negativity bias. (**c**) Relative valence bias index (rVBI, defined as (CA^+^ − CA^−^)/(|CA^+^|+|CA^−^|)) based on the ML parameters from the credibility-valence CA model. Positive values indicate a positivity bias, while negative values represent a negativity bias. Small dots represent fitted parameters for individual participants and big circles represent the group median (**a, b**) or mean (**c**) (both of participants’ behaviour), while diamonds are the median or mean of the fitted parameters of the instructed-credibility Bayesian model simulations. Error bars show the standard error of the mean. ***p < 0.001 for ML fits of participants behaviour.

**Appendix 2—figure 5.**
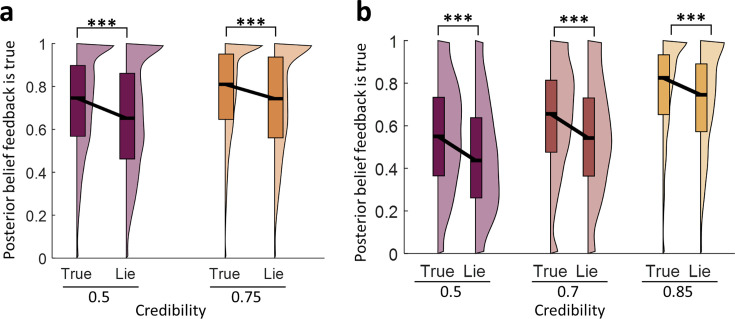
Posterior-truthfulness belief as a function of objective feedback truthfulness based on distorted credibilities from free-credibility Bayesian model fits. (**a**) Main study distributions of posterior truthfulness belief probability that feedback is true calculated separately for each agent (1-star or 2-star) and objective feedback-truthfulness (true or lie). These probabilities are based on the ML credibilities from the free-credibility Bayesian model fits for each participant. The probabilities are computed based on trial-sequences and feedback participants experienced, revealing that belief probabilities that feedback is true are higher in truth compared to lie trials, even if participants attribute distorted feedback-credibilities. For illustration, plotted distributions pool trials across participants. The black line within each box represents the median, upper and lower bounds represent the third and first quartiles, respectively. The width of each half-violin plot corresponds to the frequency of each posterior belief value among all trials for a given condition. (**b**) Same as in a, but for discovery study.

**Appendix 4—figure 1.**
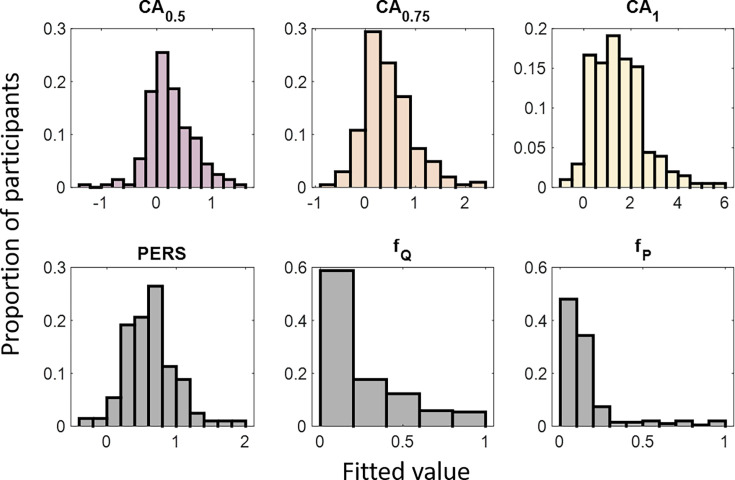
Distribution of ML parameters from participants fitted with the credibility-CA model.

**Appendix 4—figure 2.**
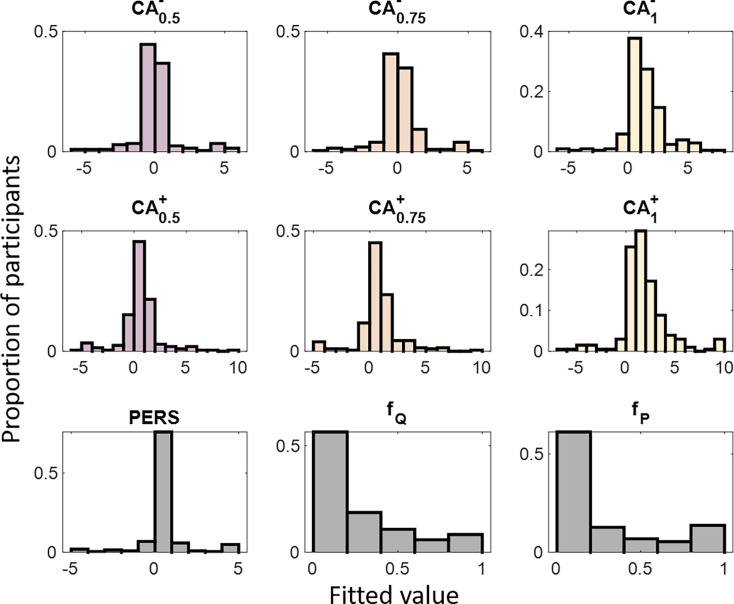
Distribution of ML parameters from participants fitted with the credibility-valence CA model.

**Appendix 4—figure 3.**
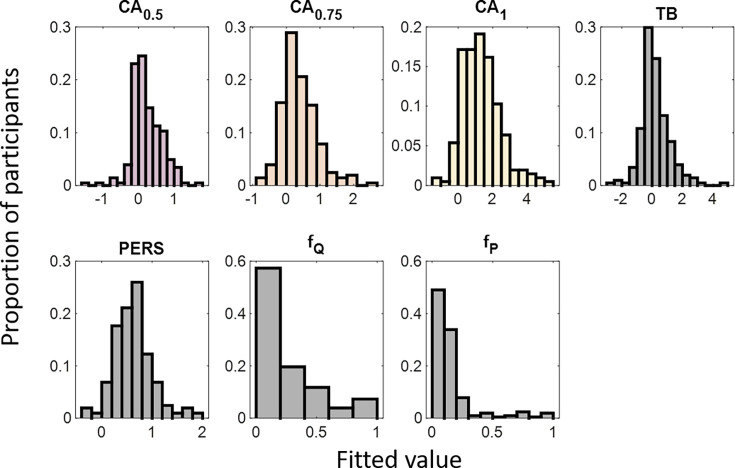
Distribution ML parameters from participants fitted with the Truth CA model.

**Appendix 4—figure 4.**
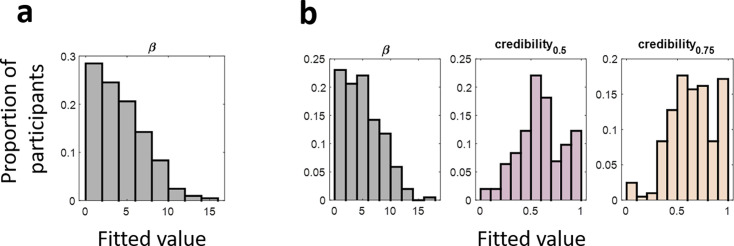
Distribution ML parameters from participants fitted with (**a**) the Instructed-credibility Bayesian model and (**b**) the free-credibility Bayesian model.

**Appendix 4—figure 5.**
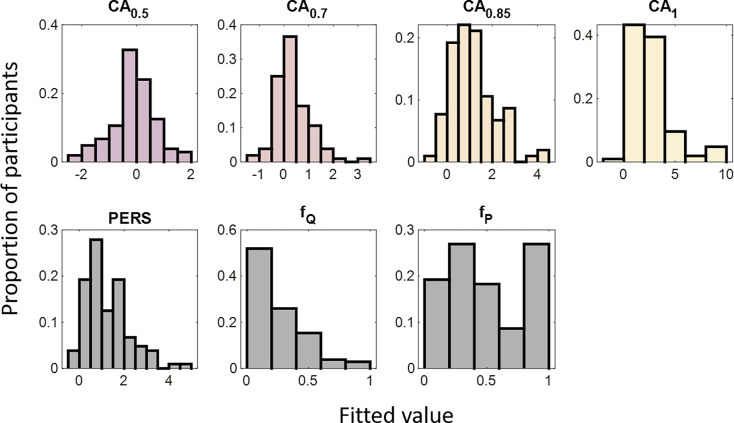
Distribution of ML parameters from participants fitted with the credibility-CA model.

**Appendix 4—figure 6.**
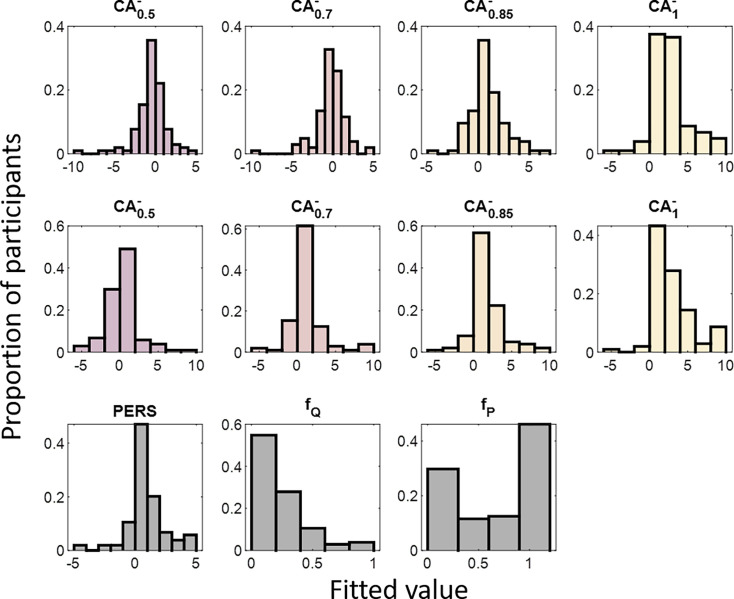
Distribution of ML parameters from participants fitted with the credibility-valence CA model.

**Appendix 4—figure 7.**
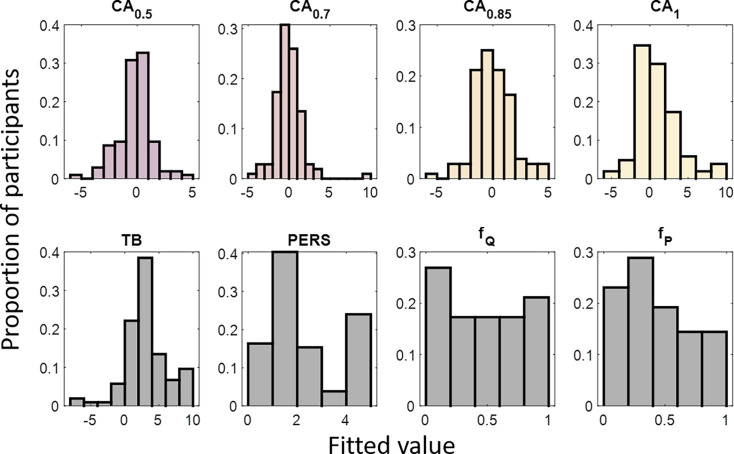
Distribution ML parameters from participants fitted with the Truth CA model.

**Appendix 4—figure 8.**
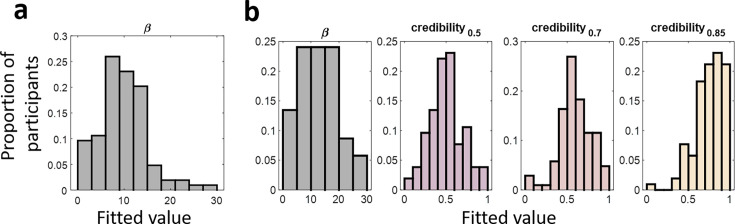
Distribution ML parameters from participants fitted with (**a**) the Instructed-credibility Bayesian model and (**b**) the free-credibility Bayesian model.

**Appendix 4—figure 9.**
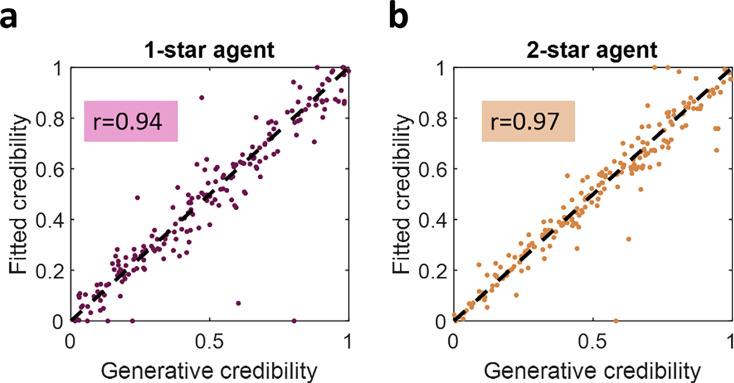
Parameter recovery for parameters of interest from free-credibility Bayesian model. (**a**) Recovery for the credibility parameter of the 1-star agent. (**b**) Recovery for the credibility parameter of the 2-star agent. Recoverability is represented by scatter plots between the generative credibility parameters used to create the synthetic datasets (*x*-axis) and the corresponding credibility parameters fitted from those datasets (*y*-axis). Circles represent the datapoints of individual simulations, the denoted metric ‘*r*’ corresponds to the Spearman correlation between the generative and fitted parameters.

**Appendix 4—figure 10.**
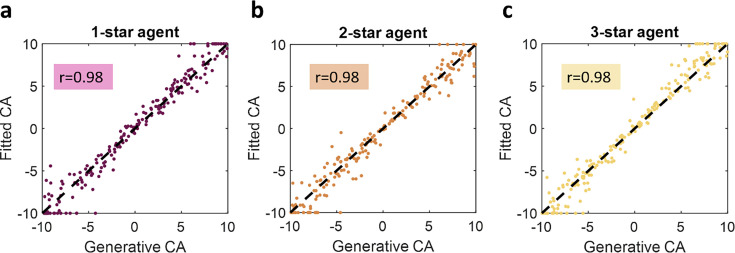
Parameter recovery for parameters of interest from credibility-CA model. Recovery for the CA parameter of the 1-star agent (**a**), 2-star agent (**b**), and 3-star agent (**c**). Recoverability is represented by scatter plots between the generative CA parameters used to create the synthetic datasets (*x*-axis) and the corresponding CA parameters fitted from those datasets (*y*-axis). Circles represent the datapoints of individual simulations, the denoted metric ‘*r*’ corresponds to the Spearman correlation between the generative and fitted parameters.

**Appendix 4—figure 11.**
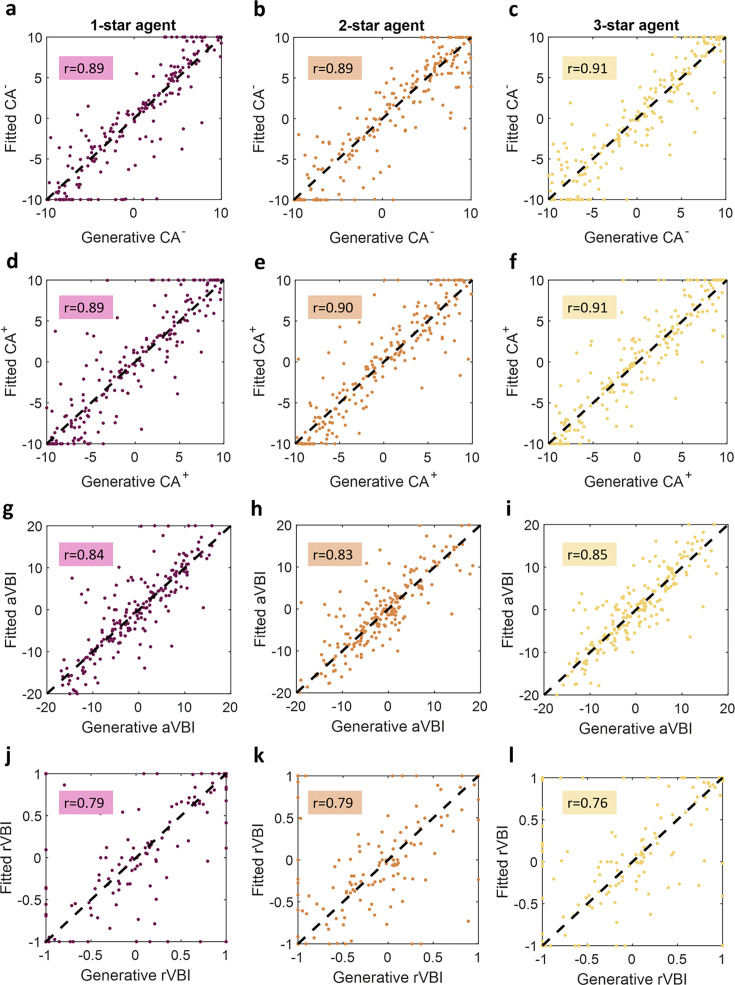
Parameter recovery for parameters and metrics of interest from credibility-valence-CA model. Recovery for the CA^−^ and CA^+^ parameters (**top two rows**) and their associated absolute valence bias (aVBI) and relative valence bias (rVBI) (**bottom two rows**) for the 1-star agent (**a, d, g, j**), 2-star agent (**b, e, h, k**), and 3-star agent (**c, f, i, l**). Recoverability is represented by scatter plots between the generative parameters/metrics used to create the synthetic datasets (*x*-axis) and the corresponding parameters/metrics fitted from those datasets (*y*-axis). Circles represent the datapoints of individual simulations, the denoted metric ‘*r*’ corresponds to the Spearman correlation between the generative and fitted parameters.

**Appendix 4—figure 12.**
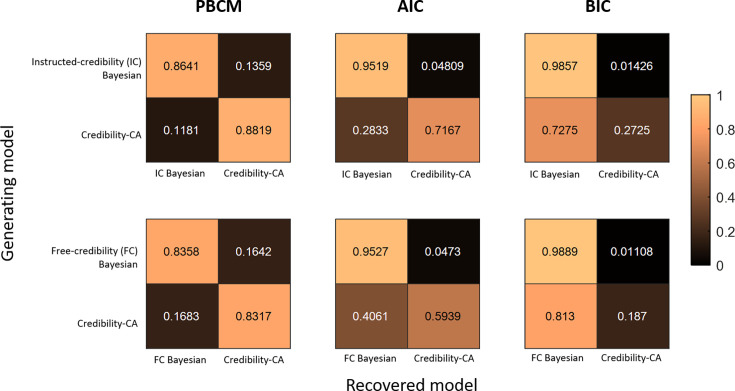
Model recovery assessment using parametric bootstrap cross-fitting method (PBCM), AIC, and BIC. We ran a model recovery analysis to assess how well different model selection methods identify the generative models. We report confusion matrices for comparisons between each Bayesian model variant (instructed-credibility: top; free-credibility: bottom matrices) and the credibility-CA model (without perseveration) for 3 model-comparison methods (PBCM-left; AIC-middle, BIC-right matrices). Each matrix cell displays the percentage of simulated datasets generated by the ‘row model’ where the ‘column model’ provided a better fit (e.g., the top right cell describes the proportion of datasets generated by a Bayesian variant, which were better fit by the credibility-CA model). These proportions were calculated according to the following method (applied separately for each compared model-pair). For each model under comparison (Bayesian or credibility-CA), we created 100 synthetic datasets per participant using their empirical maximum likelihood (ML) parameter estimates. Each of these synthetic datasets was then fitted with both models. Next, we calculated, for each participant (and each model-comparison method), individual-level 2 × 2 confusion matrices displaying the proportion of that individual’s ‘row model’ generated datasets (out of 100) that was best fit by each of the two models. Finally, the matrices shown in the figure represent the across-participant average of these individual-level confusion matrices. The results clearly demonstrate that the PBCM provides an unbiased recovery of both the Bayesian and the credibility-CA models, while achieving the highest rate of correct classifications (the average of main diagonal matrix cells). In contrast, both the AIC and BIC methods exhibit a significant bias towards selecting the Bayesian models, with a substantial (majority for BIC) selection of a Bayesian model as the winning model for data generated by the CA model.

**Appendix 5—figure 1.**
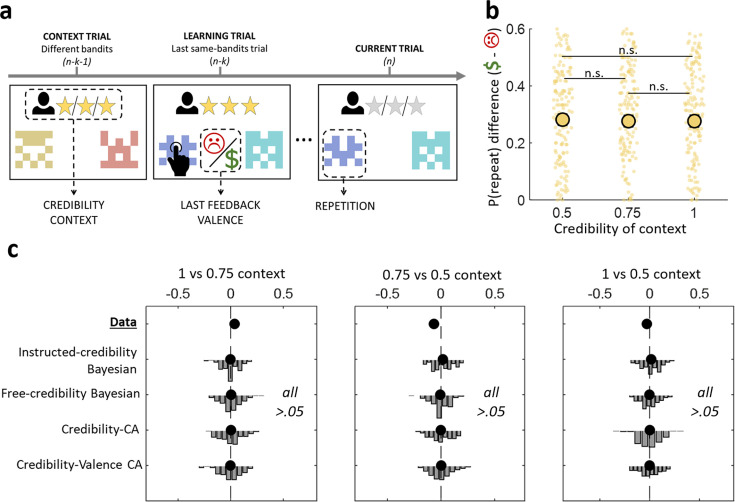
Contextual effects and learning when the context trial features a different bandit pair than the learning trial. (**a**) Trials contributing to the analysis of effects of credibility-context on learning from the fully credible agent. We included the same trials as in our main analysis, with one key difference: we only included context trials featuring a different bandit pair than the learning and current trial. We examined how choice-repetition (from *n*− *k* to *n*) was modulated by feedback valence on the learning trial, and on the feedback agent on the context trial. Note the greyed-out star-rating on the current trial indicates the identity of the current agent and was not included in the analysis. (**b**) Difference in probability of repeating a choice after receiving positive vs. negative feedback (i.e., feedback effect) from the 3-star agent, as a function of the credibility context. We found no significant effect of the credibility context on learning from the 3-star agent. (**c**) Difference in feedback valence effect on choice-repetition between contextual credibility pairs based on synthetic simulations of our alternative models. Histograms represent the distribution of regression coefficients based on 101 group-level synthetic datasets simulated based on each model. Participants’ results were within the range of effects predicted by our main models (more than 5% of group-level simulations predicted and equal or stronger effect). Big circles represent the group mean, and error bars show the standard error of the mean. *p < 0.05, **p < 0.01.

**Appendix 6—figure 1.**
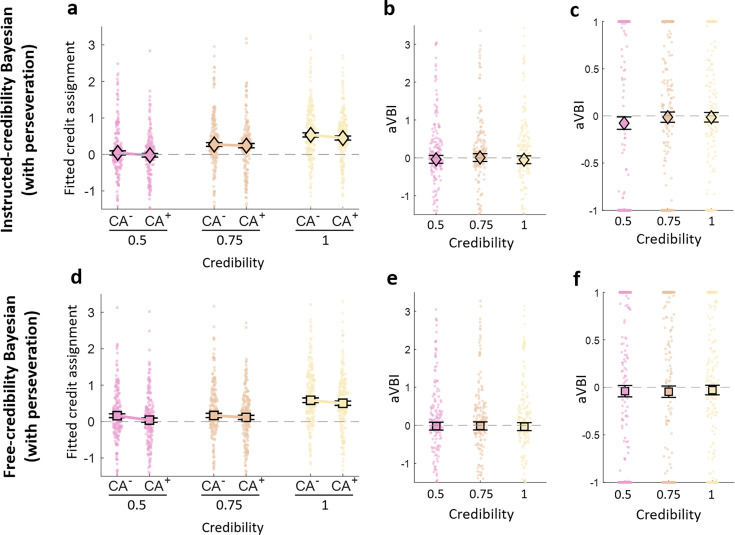
Predicted positivity bias as a function of agent-credibility based on Bayesian account including perseveration. (**a**) ML parameters from fitting simulations of the instructed-credibility Bayesian model (with perseveration) with the credibility-valence-CA model. Simulations predict a negativity bias (CA^+^ < CA^−^) for all credibility levels. (**b**) Absolute valence bias index (defined as CA^+^ − CA^−^) based on the ML parameters from the credibility-valence CA model. Positive values indicate a positivity bias, while negative values represent a negativity bias. (**c**) Relative valence bias index (defined as (CA^+^ − CA^−^)/(|CA^+^| + |CA^−^|)) based on the ML parameters from the credibility-valence CA model. Positive values indicate a positivity bias, while negative values represent a negativity bias. (**d–f**) Same as a–c, but for simulations based in the extended version of the free-credibility Bayesian model (including perseveration). Small dots represent fitted parameters for individual participants and big diamonds/squares represent the group median (**a, b, d, e**) or mean (**c, f**) for the instructed/free-credibility Bayesian model simulations. Error bars show the standard error of the mean.

**Appendix 6—figure 2.**
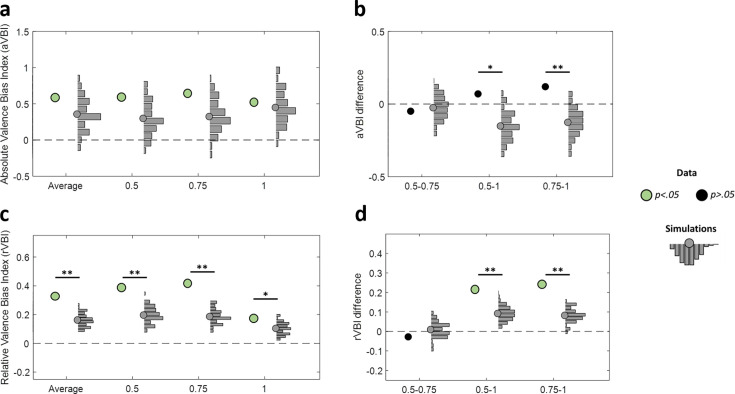
Predicted positivity bias results for participants and for simulations of the credibility-CA (including perseveration, but no valence-bias component). (**a**) Valence bias results measured in absolute terms (by regressing the ML CA parameters on their associated valence and credibility). (**b**) Difference in positivity bias (measured in absolute terms) across credibility levels. On the *x*-axis, the hyphen (-) represents subtraction, such that a label of '0.5–1' indicates the difference in the measurement for the 0.5 and 1.0 credibility conditions. Such differences are again based in the same mixed-effects model as plot a. The inflation of aVBI for lower-credibility agents is larger than the one predicted by a pure perseveration account. (**c**) Valence bias results measured in relative terms (by regressing the rVBIs on their associated credibility). Participants present a higher rVBI than what would be predicted by a perseveration account (except for the completely credible agent). (**d**) Difference in rVBI across credibility levels. Such differences are again based in the same mixed-effects model as plot c. The inflation of rVBI for lower-credibility agents is larger than the one predicted by a pure perseveration account. Histograms depict the distribution of coefficients from 101 simulated group-level datasets generated by the credibility-CA model and fitted with the credibility-valence CA model. Gray circles represent the mean coefficient from these simulations, while black/green circles show the actual regression coefficients from participant behaviour (green for significant effects in participants, black for non-significant). Significance markers (*p < 0.05, **p < 0.01) indicate that fewer than 5% or 1% of simulated datasets, respectively, predicted an effect as strong as or stronger than that observed in participants, and in the same direction as the participant effect.

**Appendix 6—figure 3.**
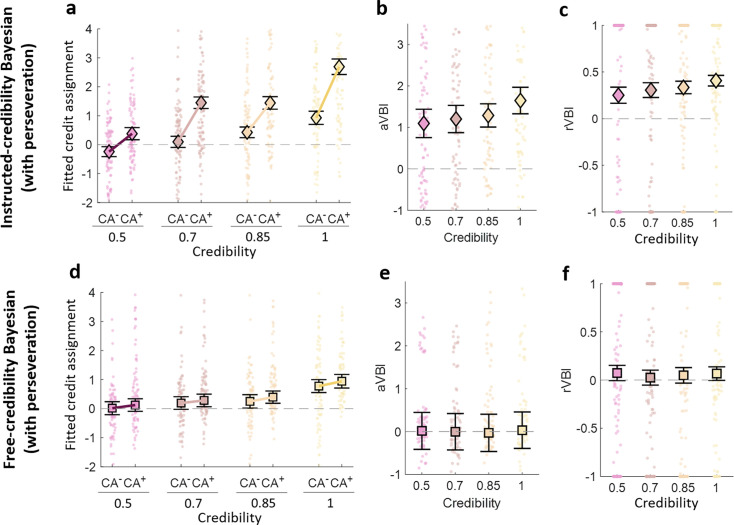
Predicted positivity bias in discovery study as a function of agent-credibility based on Bayesian account including perseveration. (**a**) ML parameters from fitting simulations of the instructed-credibility Bayesian model (with perseveration) with the credibility-valence-CA model. Simulations predict a positivity bias (CA^+^ > CA^−^) for all credibility levels. (**b**) Absolute valence bias index (defined as CA^+^ − CA^−^) based on the ML parameters from the credibility-valence CA model. Positive values indicate a positivity bias, while negative values represent a negativity bias. (**c**) Relative valence bias index (defined as (CA^+^ − CA^−^)/(|CA^+^|+|CA^−^|)) based on the ML parameters from the credibility-valence CA model. Positive values indicate a positivity bias, while negative values represent a negativity bias. (**c–f**) Same as a–c, but for simulations based in the extended version of the free-credibility Bayesian model (including perseveration). Small dots represent fitted parameters for individual participants and big diamonds/squares represent the group median (**a, b, d, e**) or mean (**c, f**) for the instructed/free-credibility Bayesian model simulations. Error bars show the standard error of the mean.

**Appendix 6—figure 4.**
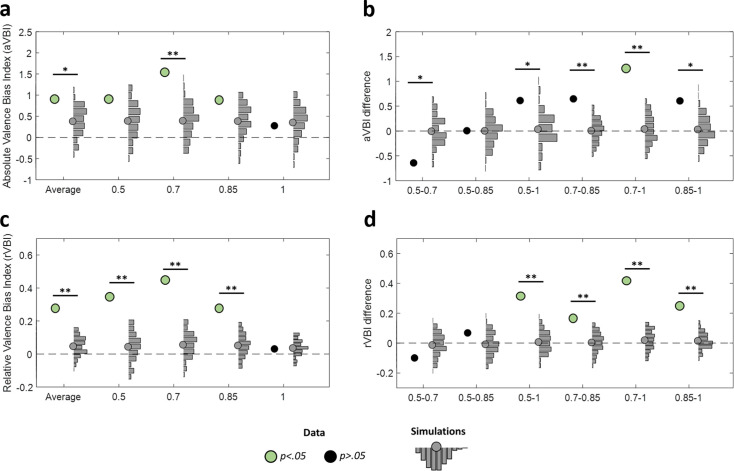
Predicted positivity bias results for participants and for simulations of the credibility-CA (including perseveration, but no valence-bias component) in discovery study. (**a**) Valence bias results measured in absolute terms (by regressing the ML CA parameters on their associated valence and credibility). (**b**) Difference in positivity bias (measured in absolute terms) across credibility levels. On the *x*-axis, the hyphen (-) represents subtraction, such that a label of '0.5–1' indicates the difference in the measurement for the 0.5 and 1.0 credibility conditions. Such differences are again based in the same mixed-effects model as plot a. The inflation of aVBI for lower-credibility agents is larger than the one predicted by a pure perseveration account. (**c**) Valence bias results measured in relative terms (by regressing the rVBIs on their associated credibility). Participants present a higher rVBI than what would be predicted by a perseveration account (except for the completely credible agent). (**d**) Difference in rVBI across credibility levels. Such differences are again based in the same mixed-effects model as plot c. The inflation of rVBI for lower-credibility agents is larger than the one predicted by a pure perseveration account. Histograms depict the distribution of coefficients from 101 simulated group-level datasets generated by the credibility-CA model and fitted with the credibility-valence-CA model. Grey circles represent the mean coefficient from these simulations, while black/green circles show the actual regression coefficients from participant behaviour (green for significant effects in participants, black for non-significant). Significance markers (*p < 0.05, **p < 0.01) indicate that fewer than 5% or 1% of simulated datasets, respectively, predicted an effect as strong as or stronger than that observed in participants, and in the same direction as the participant effect.

**Appendix 6—figure 5.**
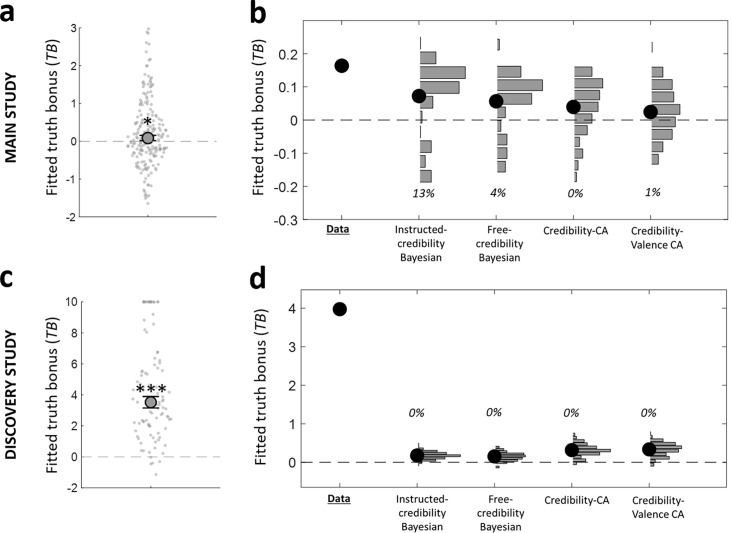
Credit assignment is enhanced for feedback inferred to be true, even when controlling for positivity bias. (**a**) Maximum likelihood (ML) estimate of the ‘truth-bonus’ parameter derived from the ‘Truth-CA’ model with valence bias in main study. (**b**) Distribution of truth-bonus parameters predicted by synthetic simulations of our alternative computational models in main study. For each alternative model, we generated 101 group-level synthetic datasets based on the maximum likelihood parameters fitted to the participants' actual behaviour. Each of these synthetic datasets was then independently fitted with the ‘Truth-CA’ model with valence bias. Each histogram represents the distribution of the mean truth bonus across the 100 simulated datasets for a specific alternative model. Notably, the truth bonus observed in our participants was significantly higher than the truth bonus predicted by all alternative models, with the exception of the instructed-credibility Bayesian model. (**c, d**) Same as a, b, but for discovery study. *p < 0.05, ***p < 0.001

## Data Availability

Code and data used to generate the results and figures in this paper is available at https://github.com/Juan-VidalPerez/DisinformationElicitsLearningBiases (copy archived at Vidal-Perez, 2026).

## Additional files

MDAR checklist

## Appendix 1

### Discovery study

#### Methods

The methods in our discovery study were similar to the ones in our main study. Here, we specify only the methodological differences between the two studies.

##### Participants, general protocol, and exclusions

We recruited 111 participants (mean age 36.23 ± 11.26, 50 female) from the Prolific participant pool (https://www.prolific.co) who performed the task in the Gorilla platform (Anwyl-Irvine et al., 2020). All participants were fluent English speakers with normal or corrected-to-normal vision and a Prolific approval rate of 95% or higher. The UCL Research Ethics Committee approved the study (Project ID 6649/004), and all participants provided informed consent before the experiment.

The participants took on average 60 min to complete the experiment. They received a fixed compensation of 6 GBP and variable compensation between 0 and 2 GBP based on their performance on the disinformation task.

Based on the same exclusion criteria from the main task, 7 participants were excluded, while 104 participants were kept for the analyses.

##### Task differences between discovery study and main study

In the discovery study participants also completed a traditional version of the two-armed bandit task. The task was like the one in the main study, but each block featured a single bandit pair. Participants completed 6 blocks in total (of 16 trials each), 3 before the disinformation task and 3 right after.

The disinformation task in the discovery study worked as the one in the main study, but with three main differences. Firstly, it included four feedback agents, with credibilities of 50%, 70%, 85%, and 100%, represented by 1, 2, 3, and 4 stars, respectively (Appendix 1—figure 1a). Each experimental block consisted of a single bandit pair, presented over 16 trials (with 4 trials for each feedback agent). Participants completed a total of 15 blocks: in 5 of them the bandits had 25% and 75% probability; while in the remaining 10 blocks, the two bandits within a pair were equally rewarding, with a reward probability randomly sampled between 60% and 80%.

At the end of the experiment, a subset of participants (*n* = 79) completed eight standard self-report questionnaires: (1) the Obsessional Compulsive Inventory—Revised (OCI-R) (Foa et al., 2002), assessing symptoms of obsessive–compulsive disorder (OCD); (2) the Revised Green et al. Paranoid Thoughts Scale (R-GPTS) (Freeman et al., 2021), measuring paranoid ideations; (3) the DOG scale (Altemeyer, 2002), evaluating dogmatism; (4) the autism-spectrum quotient (AQ) (Baron-Cohen et al., 2001), assessing symptoms of autism spectrum disorder; (5) the Adult ADHD Self-Report Scale (ASRS) (Kessler et al., 2005), for attention-deficit/hyperactivity disorder; (6) the GAD-7 scale (Spitzer et al., 2006) for generalized anxiety disorder; (7) the self-rating depression scale (SDS) (Zung, 1965); and (8) the Barratt Impulsiveness scale (BIS) for impulsivity (Stanford et al., 2009).

##### Computational models

###### RL models

The RL models in the discovery task were analogous to the ones in the main task but included extra free CA parameters to accommodate the use of four (instead of three) feedback agents.

**Appendix 1—table 1.** Summary of free parameters for each of the CA models.

Model	Free CA parameter
*Null*	*CA*
Credibility-CA	$CA_{0.5},CA_{0.7}, CA_{0.85},CA_{1}$
Credibility-valence-CA	$CA_{0.5}+,CA_{0.7}+, CA_{0.85}+,CA_{1}+$
	$CA_{0.5}-,CA_{0.7}-, CA_{0.85}-,CA_{1}-$
Constant feedback-valence bias CA	*VB*
	$CA_{0.5}-,CA_{0.7}-, CA_{0.85}-,CA_{1}-$
Truth-CA	*TB*
	$CA_{0.5},CA_{0.7}, CA_{0.85},CA_{1}$

###### Bayesian models

The Bayesian models in the discovery study worked like the one in the main study. In the *free-credibility Bayesian model*, we fixed the 4-star agent-credibility to 1 but estimated the credibility of the three lying agents as free parameters.

#### Mixed-effects models

The mixed-effects models in our discovery study used the same regressors as in our main study, replacing (AGENT_2-star_ + AGENT_3-star_) with (AGENT_2-star_ + AGENT_3-star_ + AGENT_4-star_) to account for the fact that the task featured four (instead of three agents). Moreover, when regressing the CA parameters from truth-CA model on agent-credibility and feedback truthfulness, we replaced the regressor *CREDIBILITY* with regressors indicating the presence/absence of the 2-star and 3-star agents (AGENT_2-star_ + AGENT_3-star_).

#### Results

##### Credible feedback promotes greater learning

To test whether participants modulated their learning based on feedback credibility, we regressed in a binomial mixed-effects model, choice-repetition, on feedback-valence (negative or positive) and the agent-credibility (1-, 2-, 3-, or 4-star) from the last trial (Appendix 1—figure 2a) (see Appendix 1—Section 1.3 for full model description). Consistent with findings in the main task, we found that feedback valence exerted a positive effect on choice-repetition (*b* = 1.02, *F*(1,2462) = 617.38, p < 0.001), and it interacted with agent-credibility (*F*(3,2462) = 196.61, p < 0.001), such that the feedback effect was larger for more credible agents (4-star vs. 3-star: *b* = 1.21, *F*(1,2462) = 137.71; 4-star vs. 2-star: *b* = 0.47, *F*(1,2462) = 322.88; 4-star vs. 1-star: *b*=2.55, *t*(2462) = 22.91; 3-star vs. 2-star: *b* = 2.03, *F*(1,2462) = 40.54; 3-star vs. 1-star: *b* = 1.24, *t*(2462) = 11.25; and 2-star vs. 1-star: *b* = 0.52, *t*(2462) = 4.74, all p’s<0.001). Additionally, we found a positive feedback-effect for the 4-star agent (*b* = 2.46, *F*(1,2462) = 911.81, p < 0.001), a smaller feedback-effect for the 3-star agent (*b* = 1.15, *F*(1,2462) = 206.19, p < 0.001), and an even smaller feedback-effect for the 2-star agent (*b* = 2.03, *F*(1,2462) = 40.54, p < 0.001). Such increased learning as a function of feedback credibility was predicted by simulations based on the instructed-credibility Bayesian, free-credibility Bayesian and credibility-CA models, but not by the null-CA model (Appendix 1—figure 2b).

We next examined how the maximum likelihood (ML) CA parameters from the credibility-CA model differed as a function of feedback credibility (Appendix 1—figure 2c; see Appendix 4 —Section 3.2 for detailed ML parameter results). We regressed, using a mixed-effects model (Methods and Appendix 1—Section 1.4), the CA parameters on their associated agent, showing that CA differed across the agents (*F*(3,412) = 98.77, p < 0.001), increasing as a function of agent-credibility (4-star vs. 3-star: *b* = 0.49, *F*(1,412) = 99.94; 4-star vs. 2-star: *b* = 0.89, *F*(1,412) = 330.15; 4-star vs. 1-star: *b* = 1.4, *Ft*(412) = 28.5; 3-star vs. 2-star: *b* = 0.4, *F*(1,412) = 66.8; 3-star vs. 1-star: *b* = 0.91, *t*(412) = 18.5; and 2-star vs. 1-star: *b* = 0.51, *t*(412) = 10.33, all p’s < 0.001).

##### Substantial deviations from Bayesian learning

Model comparisons between each of the Bayesian models and the credibility-CA revealed that the credibility-CA model provided a superior fit for 80.8% of participants (sign test; *z* = 6.17, p < 0.001) when compared to the instructed-credibility Bayesian model (Appendix 1—figure 1c), and for 60.6% (*z* = 2.06, p = 0.03) when compared with the free-credibility Bayesian model (Appendix 1—figure 1d). In line with the main study, this suggests most of the participants deviated from normative learning.

The Bayesian-CA parameters revealed that both the instructed-credibility and free-credibility Bayesian models predicted increased Bayesian-CA parameters as a function of agent-credibility (Appendix 1—figure 2c; see Appendix 3—Section 1.2.2). We next test whether the different deviations from normative Bayesian learning that we described in the main study are still present in the discovery study.

##### The effect of non-credible feedback and learning

In the main study, we found evidence supporting the idea that participants update their beliefs based on random feedback (a positive feedback effect for the 1-star agent on choice-repetition and a positive CA parameter). However, corresponding analyses for the discovery task revealed neither a feedback-effect on choice-repetition (mixed-effects model, *b* = −0.09, *t*(2462) = −1.21, p = 0.22; Appendix 1—figure 2a), nor positive credit assignment (*b* = −0.13, *t*(412) = −1.07, p = 0.28; Appendix 1—figure 2c) for the 1-star agent. Moreover, based on the free-credibility Bayesian model, we found no evidence ML-estimated credibility for the 1-star agent differed from 0.5 (Wilcoxon signed-rank test, median = −0.02, *z* = −0.29, p = 0.76; Appendix 1—figure 2d). Importantly, we show below that feedback from this agent was not fully ignored as it still elicited a positivity bias. Incidentally, using the free-credibility Bayesian model we found the ML-estimated credibility increased as a function of instructed agent-credibility (Wilcoxon signed-rank test; 3-star vs. 2-star, median = 0.13, *z* = 6.75; 3-star vs. 1-star, median = 0.28, *z* = 7.45; 2-star vs. 1-star, median = 0.10, *z* = 4.54; all p’s < 0.001), and was lower than the instructed-credibility for the 2-star (median = −0.06, *z* = 4.90, p < 0.001) and 3-star agents (median = −0.27, *z* = −4.49, p < 0.001). In line with the main study, this finding suggests that participants tend to underestimate the credibility of agents with intermediate levels of credibility.

##### Exaggerated learning for fully credible feedback

Consistent with the main study, both Bayesian models predicted an attenuated credit assignment for the fully credible agent (Wilcoxon signed-rank test; instructed-credibility Bayesian model: median difference = 0.67, *z* = 6.70; free-credibility Bayesian model: median difference = 0.26, *z* = 4.28, all p’s < 0.001). We did not analyse the effects of the credibility context on learning, since this task affords no instances where the credibility context featured a separate bandit pair (because our discovery task featured a single bandit pair per block).

##### Individuals show a positivity bias in learning, particularly for sources of limited credibility

Next, we turned to test whether, in the discovery study, participants showed greater learning from positive (instead of negative) feedback. We regressed in a mixed-effects model the ML parameters from the credibility-valence-CA model (Appendix 1—figure 3a; see Appendix 4—Section 3.2 for detailed ML parameter results) on their associated agent-credibility and valence (see Appendix 1—Methods). As in the main study, this revealed an overall positivity bias in credit assignment (*b* = 0.94, *F*(1,824) = 42.60, p < 0.001). Furthermore, participants assigned negative credit based on negative feedback from the 1-star agent (*b* = −0.51, *F*(1,824) = 3.89, p = 0.049), and positive credit based on positive feedback from the same agent (*b* = 0.42, *F*(1,824) = 5.74, p = 0.017). Critically, this suggests that in agreement with conclusions from the main task, participants do not ignore random feedback. Instead, at the group level, both negative and positive feedback from the 1-star agent led to a value increase of the selected bandit. Participants selectively assigned positive credit to positive feedback from the 2-star agent (*b* = 1.25, *F*(1,824) = 16.34, p < 0.001), with no evidence for CA based on negative feedback (*b* = −0.34, *F*(1,824) = 2.53, p = 0.11). For the 3-star and 4-star agents, credit assignment was positive for both positive (3-star: *b* = 2.28, *F*(1,824) = 71.28; 4-star: *b* = 2.91, *F*(1,824) = 183.22; p < 0.001) and negative (3-star: *b* = 0.86, *F*(1,824) = 37.77; 4-star: *b* = 2.60, *F*(1,824) = 146.55; p < 0.001) feedback.

In line with the main task, free-credibility Bayesian-CA parameters revealed a *negativity* bias (*b* = −0.71, *F*(1,824) = 49.8, p < 0.001; Appendix 1—figure 3a), and lower absolute valence bias indices than the ones from participants for all credibility levels (Appendix 1—figure 3b) [Wilcoxon signed-rank test, 50% credibility (median difference = 1.43, *z* = 3.99, p < 0.001), 70% credibility (median difference = 1.70, *z* = 5.44, p < 0.001), 85% credibility (median difference = 1.59, *z* = 4.78, p < 0.001) and 100% credibility (median difference = 1.06, *z* = 3.06, p = 0.002)]. Results supporting the same conclusions were observed for instructed-credibility Bayesian-CA parameters (see Appendix 3—Section 2.3 CA, feedback valence and feedback credibility and Appendix 4—Section 2.1 Comparison of empirical-aVBI and Bayesian-aVBI). These results confirm that the detected positivity bias represents a departure from normative Bayesian learning.

In the main study, we found no group-level evidence positivity-bias was modulated by agent-credibility in absolute terms. However, in the discovery study, the mixed-effects regressing the credibility-valence-CA model parameters (Appendix 1—figure 3a, b) revealed a significant interaction effect between feedback valence and credibility on CA (*F*(3,824) = 3.28, p = 0.02), such that the valence effect for the 2-star agent was larger than the one for the 4-star gent (*b* = 1.29, *F*(1,824) = 9.84, p = 0.002), with no significant valence effect difference between other agent pairs (see Appendix 3—table 16). Moreover, while the valence effect for the lying agents was significantly positive (1-star: *b* = 0.94, *t*(824) = 3.24, p < 0.001; 2-star: *b* = 1.60, *F*(1,824) = 30.21, p = 0.001; 3-star: *b* = 0.94, *F*(1,824) = 10.63, p = 0.001), we found no evidence for a positivity bias for the 4-star agent (*b* = 0.31, *F*(1,824) = 1.12, p = 0.29). In contrast, we found no evidence for an interaction between feedback valence and credibility based on free-credibility Bayesian-CA (Appendix 1—figure 3b; *F*(3,824) = 0.11, p = 0.95) and instructed-credibility Bayesian-CA (*F*(3,824) = 0.25 p = 0.85) parameters. These results suggest that participants show a heightened positivity bias (measured in absolute terms) in response to low-credibility feedback.

The positivity bias measured relative to the overall extent of learning (i.e., the rVBI) was significantly positive for low-credibility feedback [50% credibility (*b* = 0.35, *t*(412) = 5.58), 70% credibility (*b* = 0.45, *F*(1,412) = 51.44), 85% credibility (*b* = 0.28, *F*(1,412) = 20.12), all p’s < 0.001] (Appendix 1—figure 3c). However, we found no evidence for positive rVBI for the fully credible agent (*b* = 0.03, *F*(1,412) = 0.25, p = 0.62). Moreover, in line with the main study, we found that the rVBI varied depending on the credibility of feedback (*F*(3,412) = 12.33, p < 0.001), such that the rVBI for 4-star agent was lower than the one for any of the low-credibility agents [50% credibility (*b* = −0.32, *t*(412) = −4.44), 70% credibility (*b* = −0.42, *F*(1,412) = 33.88), 85% credibility (*b* = −0.25, *F*(1,412) = 12.1), all p’s < 0.001]. The rVBI for 70% credibility agent was higher than the one for the 85% credibility agent (*b* = −0.17, *F*(1,412) = 5.49, p = 0.019). Feedback with 50% credibility yielded similar rVBI values to feedback with 70% (*b* = 0.10, *t*(412) = 1.39, p = 0.17) or 85% credibility (*b* = −0.07, *t*(412) = −0.96, p = 0.34). Notably, our rVBI results were not predicted by either the free-credibility Bayesian-CA (Appendix 1—figure 3c) and instructed-credibility Bayesian-CA parameters (see Appendix 4—Section 2.2), nor by a pure choice-perseveration account (see Appendix 6—Section 2). These results support the same conclusion from the main study, suggesting that positivity bias, relative to the overall extent of CA, is higher for lying compared to fully credible agents.

##### True feedback elicits greater learning

We next assessed whether participants infer whether the feedback they received on each trial was true or false and adjust their credit assignment based on this inference. We again used the ‘Truth-CA’ model to obtain estimates for the truth bonus (*TB*), the increase in credit assignment as a function of the posterior probability of feedback being true. As in our main study, the fitted truth bias parameter was significantly positive, indicating that participants assign greater weight to feedback they believe is likely to be true (Appendix 1—figure 4a; see Appendix 1–Main study for detailed ML parameter results). Strikingly, model simulations (Methods) predicted a lower truth bonus than the one observed in participants (Appendix 1—figure 4b).

## Appendix 3

### Mixed-effects model results

#### Main study

##### Choice-repetition and feedback credibility

In this section, we provide full result tables for mixed-effects models used in the sections ‘Credible feedback promotes greater learning’ and ‘Non-credible feedback elicits learning’; and Figure 3a, b. The Wilkinson’s notation of the model is:

$$\displaystyle REPEAT{\sim }FEEDBACK*BETTER*\left (AGENT_{2-star}+AGENT_{3-star}\right)+\left (1|participant\right)$$

**Appendix 3—table 1.** Mixed-effects binomial regression model regressing choice-repetition on feedback-valence, agent-credibility and better/worse choice from previous trial featuring the same bandit pair. Based on participants’ data. Feedback effect increased as a function of agent-credibility (3-star vs. 2-star: *b* = 0.91, *F*(1,2436) = 351.17; 3-star vs. 1-star: *b* = 1.15, *t*(2436) = 24.02; and 2-star vs. 1-star: *b* = 0.24, *t*(2436) = 5.34, all p’s < 0.001). Feedback valence exerted a positive effect for the 1-star agent (*b* = 0.25, *t*(2436) = 8.05, p < 0.001).

PARTICIPANTS DATA							
Name	Estimate	SE	tStat	DF	pValue	Lower	Upper
(Intercept)	0.99	0.04	25.27	2436	<0.001	0.92	1.07
FEEDBACK	0.25	0.03	8.05	2436	<0.001	0.19	0.31
BETTER	0.58	0.03	18.41	2436	<0.001	0.51	0.64
AGENT(2-STAR)	0.01	0.02	0.48	2436	0.62	–0.03	0.05
AGENT(3-STAR)	–0.13	0.02	–5.43	2436	<0.001	–0.18	–0.08
FEEDBACK:BETTER	–0.02	0.06	–0.25	2436	0.8	–0.14	0.11
FEEDBACK:AGENT(2-STAR)	0.24	0.04	5.34	2436	<0.001	0.15	0.33
BETTER:AGENT(2-STAR)	–0.07	0.04	–1.63	2436	0.1	–0.16	0.01
FEEDBACK:AGENT(3-STAR)	1.16	0.05	24.02	2436	<0.001	1.06	1.25
BETTER:AGENT(3-STAR)	–0.13	0.05	–2.73	2436	<0.001	–0.23	–0.04
FEEDBACK:BETTER:AGENT(2-STAR)	0.07	0.09	0.82	2436	0.42	–0.1	0.25
FEEDBACK:BETTER:AGENT(3-STAR)	0.07	0.1	0.71	2436	0.48	–0.12	0.26
**Any interaction between FEEDBACK and AGENT:** *F*(2,2436) = 307.11, p < 0.001							
**FEEDBACK:AGENT(3-STAR) vs. FEEDBACK:AGENT(2-STAR**): *b* = 0.91, *F*(1,2436) = 351.17, p < 0.001							

**Appendix 3—table 2.** Mixed-effects binomial regression model regressing choice-repetition on feedback-valence, agent-credibility and better/worse choice from previous trial featuring the same bandit pair. Based on instructed-credibility Bayesian model simulations. Feedback effect increased as a function of agent-credibility (3-star vs. 2-star: *b* = 0.47, *F*(1,2436) = 581.93; 3-star vs. 1-star: *b* = 0.86, *t*(2436) = 44.52; and 2-star vs. 1-star: *b* = 0.39, *t*(2436) = 21.22, all p’s < 0.001). Feedback valence did not exert a positive effect for the 1-star agent (*b* = −0.01, *t*(2436) = −0.41, p = 0.68).

INSTRUCTED-CREDIBILITY BAYESIAN MODEL							
**Name**	**Estimate**	**SE**	**tStat**	**DF**	**pValue**	**Lower**	**Upper**
(**Intercept**)	0.33	0.02	15.06	2436	<0.001	0.28	0.37
**FEEDBACK**	–0.01	0.01	–0.41	2436	0.68	–0.03	0.02
**BETTER**	0.84	0.01	65.92	2436	<0.001	0.81	0.86
**AGENT(2-STAR**)	–0.03	0.01	–3.45	2436	<0.001	–0.05	–0.01
**AGENT(3-STAR**)	–0.07	0.01	–6.9	2436	<0.001	–0.09	–0.05
**FEEDBACK:BETTER**	0.03	0.03	1.27	2436	0.2	–0.02	0.08
**FEEDBACK:AGENT(2-STAR**)	0.39	0.02	21.22	2436	<0.001	0.35	0.42
**BETTER:AGENT(2-STAR**)	–0.02	0.02	–0.98	2436	0.33	–0.05	0.02
**FEEDBACK:AGENT(3-STAR**)	0.86	0.02	44.52	2436	<0.001	0.82	0.9
**BETTER:AGENT(3-STAR**)	–0.13	0.02	–6.75	2436	<0.001	–0.17	–0.09
**FEEDBACK:BETTER:AGENT(2-STAR**)	–0.07	0.04	–1.94	2436	0.052	–0.14	0.0007
**FEEDBACK:BETTER:AGENT(3-STAR**)	0.004	0.04	0.1	2436	0.92	–0.07	0.08
**Any interaction between FEEDBACK and AGENT:** *F*(2,2436) = 991.54, p < 0.001							
**FEEDBACK:AGENT(3-STAR) vs. FEEDBACK:AGENT(2-STAR**): *b* = 0.47, *F*(1,2436) = 581.93, p < 0.001							

**Appendix 3—table 3.** Mixed-effects binomial regression model regressing choice-repetition on feedback-valence, agent-credibility and better/worse choice from previous trial featuring the same bandit pair. Based on free-credibility Bayesian model simulations. Feedback effect increased as a function of agent-credibility (3-star vs. 2-star: *b* = 0.70, *F*(1,2436) = 1268.1; 3-star vs. 1-star: *b* = 0.85, *t*(2436) = 43.63; and 2-star vs. 1-star: *b* = 0.15, *t*(2436) = 7.99, all p’s < 0.001). Feedback valence exerted a positive effect for the 1-star agent (*b* = 0.12, *t*(2436) = 9.48, p = 0.68).

FREE-CREDIBILITY BAYESIAN MODEL							
**Name**	**Estimate**	**SE**	**tStat**	**DF**	**pValue**	**Lower**	**Upper**
(**Intercept**)	0.37	0.02	16.87	2436	<0.001	0.33	0.41
**FEEDBACK**	0.12	0.01	9.48	2436	<0.001	0.1	0.15
**BETTER**	0.83	0.01	64.91	2436	<0.001	0.8	0.85
**AGENT(2-STAR**)	0	0.01	–0.07	2436	0.95	–0.02	0.02
**AGENT(3-STAR**)	–0.03	0.01	–3.08	2436	0.002	–0.05	–0.01
**FEEDBACK:BETTER**	–0.02	0.03	–0.85	2436	0.39	–0.07	0.03
**FEEDBACK:AGENT(2-STAR**)	0.15	0.02	7.99	2436	<0.001	0.11	0.18
**BETTER:AGENT(2-STAR**)	–0.02	0.02	–0.82	2436	0.41	–0.05	0.02
**FEEDBACK:AGENT(3-STAR**)	0.85	0.02	43.63	2436	<0.001	0.81	0.89
**BETTER:AGENT(3-STAR**)	–0.15	0.02	–7.87	2436	<0.001	–0.19	–0.12
**FEEDBACK:BETTER:AGENT(2-STAR**)	0	0.04	–0.08	2436	0.93	–0.07	0.07
**FEEDBACK:BETTER:AGENT(3-STAR**)	–0.04	0.04	–1.07	2436	0.28	–0.12	0.03
**Any interaction between FEEDBACK and AGENT**: *F*(2,2436) = 1041.3, p < 0.001							
**FEEDBACK:AGENT(3-STAR) vs. FEEDBACK:AGENT(2-STAR**): *b* = 0.70, *F*(1,2436) = 1268.1, p < 0.001							

**Appendix 3—table 4.** Mixed-effects binomial regression model regressing choice-repetition on feedback-valence, agent-credibility, and better/worse choice from previous trial featuring the same bandit pair. Based on null-CA model simulations. The feedback effect did not interact with agent-credibility (*F*(2,2436) = 0.11, p = 0.89).

NULL-CA MODEL							
**Name**	**Estimate**	**SE**	**tStat**	**DF**	**pValue**	**Lower**	**Upper**
(**Intercept**)	0.86	0.03	25.46	2436	<0.001	0.79	0.92
**FEEDBACK**	0.69	0.01	51.25	2436	<0.001	0.66	0.72
**BETTER**	0.37	0.01	27.54	2436	<0.001	0.35	0.4
**AGENT(2-STAR**)	–0.01	0.01	–0.87	2436	0.38	–0.03	0.01
**AGENT(3-STAR**)	0.01	0.01	1.2	2436	0.23	–0.01	0.03
**FEEDBACK:BETTER**	–0.01	0.03	–0.49	2436	0.62	–0.07	0.04
**FEEDBACK:AGENT(2-STAR**)	0.004	0.02	–0.2	2436	0.84	–0.04	0.03
**BETTER:AGENT(2-STAR**)	–0.07	0.02	–3.86	2436	<0.001	–0.11	–0.04
**FEEDBACK:AGENT(3-STAR**)	0.006	0.02	0.29	2436	0.77	–0.03	0.05
**BETTER:AGENT(3-STAR**)	–0.08	0.02	–3.81	2436	<0.001	–0.12	–0.04
**FEEDBACK:BETTER:AGENT(2-STAR**)	–0.02	0.04	–0.53	2436	0.6	–0.1	0.06
**FEEDBACK:BETTER:AGENT(3-STAR**)	–0.03	0.04	–0.66	2436	0.51	–0.11	0.05
**Any interaction between FEEDBACK and AGENT**: *F*(2,2436) = 0.11, p = 0.89							
**FEEDBACK:AGENT(3-STAR) vs. FEEDBACK:AGENT(2-STAR**): *b* = 0.002, *F*(1,2436) = 0.22, p = 0.64							

**Appendix 3—table 5.** Mixed-effects binomial regression model regressing choice-repetition on feedback-valence, agent-credibility, and better/worse choice from previous trial featuring the same bandit pair. Based on credibility-CA model simulations. Feedback effect increased as a function of agent-credibility (3-star vs. 2-star: *b* = 0.96, *F*(1,2436) = 2009.5.1; 3-star vs. 1-star: *b* = 1.15, *t*(2436) = 54.5; and 2-star vs. 1-star: *b* = 0.19, *t*(2436) = 9.79, all p’s < 0.001). Feedback valence exerted a positive effect for the 1-star agent (*b* = 0.25, *t*(2436) = 18.31, p < 0.001).

CREDIBILITY-CA MODEL							
**Name**	**Estimate**	**SE**	**tStat**	**DF**	**pValue**	**Lower**	**Upper**
(**Intercept**)	0.9	0.03	26.07	2436	<0.001	0.84	0.97
**FEEDBACK**	0.25	0.01	18.31	2436	<0.001	0.22	0.28
**BETTER**	0.49	0.01	35.89	2436	<0.001	0.46	0.51
**AGENT(2-STAR**)	–0.01	0.01	–0.52	2436	0.6	–0.02	0.01
**AGENT(3-STAR**)	–0.1	0.01	–9.62	2436	*P*<0.001	–0.12	–0.08
**FEEDBACK:BETTER**	0.03	0.03	1.08	2436	0.28	–0.02	0.08
**FEEDBACK:AGENT(2-STAR**)	0.19	0.02	9.79	2436	<0.001	0.15	0.23
**BETTER:AGENT(2-STAR**)	–0.03	0.02	–1.79	2436	0.07	–0.07	0
**FEEDBACK:AGENT(3-STAR**)	1.15	0.02	54.5	2436	<0.001	1.11	1.19
**BETTER:AGENT(3-STAR**)	–0.1	0.02	–4.85	2436	<0.001	–0.14	–0.06
**FEEDBACK:BETTER:AGENT(2-STAR**)	–0.01	0.04	–0.2	2436	0.84	–0.08	0.07
**FEEDBACK:BETTER:AGENT(3-STAR**)	–0.07	0.04	–1.65	2436	0.1	–0.15	0.01
**Any interaction between FEEDBACK and AGENT**: *F*(2,2436) = 1618.8, p < 0.001							
**FEEDBACK:AGENT(3-STAR) vs. FEEDBACK:AGENT(2-STAR**): *b* = 0.96, *F*(1,2436) = 2009.5, p < 0.001							

#### CA and feedback credibility

In this section, we provide full result tables for mixed-effects models used in the sections ‘Credible feedback promotes greater learning’, ‘Most participants deviate from Bayesian Learning’, and ‘Non-credible feedback elicits learning’; and Figure 3c. The Wilkinson’s notation of the model is:

$$\displaystyle CA{\sim }AGENT_{2\mathrm{-}star}\mathrm{+}AGENT_{3\mathrm{-}star}\mathrm{+}\left (1\mathrm{|}participant\right)$$

**Appendix 3—table 6.** Mixed-effects linear regression model regressing CA on agent-credibility, based on credibility-CA fits of participants’ data. CA increased as a function of agent-credibility (3-star vs. 2-star: *b* = 1.02, *F*(1,609) = 253.73; 3-star vs. 1-star: *b* = 1.24, *t*(609) = 19.31; and 2-star vs. 1-star: *b* = 0.22, *t*(609) = 3.38, all p’s < 0.001). We found a positive CA for the 1-star agent (*b* = 0.23, *t*(609) = 4.54, p < 0.001).

PARTICIPANTS DATA							
**Name**	**Estimate**	**SE**	**tStat**	**DF**	**pValue**	**Lower**	**Upper**
(**Intercept**)	0.23	0.05	4.54	609	<0.001	0.13	0.33
**AGENT(2-STAR**)	0.22	0.06	3.38	609	<0.001	0.09	0.34
**AGENT(3-STAR**)	1.24	0.06	19.31	609	<0.001	1.12	1.37
**Any effect of AGENT:** *F*(2,609) = 212.65, p < 0.001							
**AGENT(3-STAR) vs. AGENT(2-STAR):** *b* = 1.02, *F*(1,609) = 253.73, p < 0.001							

**Appendix 3—table 7.** Mixed-effects linear regression model regressing CA on agent-credibility, based on credibility-CA fits of instructed-credibility Bayesian model simulations. CA increased as a function of agent-credibility (3-star vs. 2-star: *b* = 0.33, *F*(1,609) = 233.17; 3-star vs. 1-star: *b* = 0.61, *t*(609) = 28.55; and 2-star vs. 1-star: *b* = 0.28, *t*(609) = 13.28, all p’s < 0.001). There was no evidence that CA for the 1-star agent differed from 0 (*b* = −0.01, *t*(609) = −0.31, p = 0.76).

INSTRUCTED-CREDIBILITY BAYESIAN SIMULATIONS							
**Name**	**Estimate**	**SE**	**tStat**	**DF**	**pValue**	**Lower**	**Upper**
(**Intercept**)	–0.01	0.02	–0.64	609	0.54	–0.06	0.03
**AGENT(2-STAR**)	0.30	0.02	11.22	609	<0.001	0.24	0.35
**AGENT(3-STAR**)	0.60	0.02	22.93	609	<0.001	0.55	0.66
**Any effect of AGENT:** *F*(2,609) = 262.96, p < 0.001							
**AGENT(3-STAR) vs. AGENT(2-STAR):** *b* = 0.30, *F*(1,609) = 136.94, p < 0.001							

**Appendix 3—table 8.** Mixed-effects linear regression model regressing CA on agent-credibility, based on credibility-CA fits of free-credibility Bayesian model simulations. CA increased as a function of agent-credibility (3-star vs. 2-star: *b* = 0.5, *F*(1,609) = 272.63; 3-star vs. 1-star: *b* = 0.61, *t*(609) = 20.05; and 2-star vs. 1-star: *b* = 0.11, *t*(609) = 3.54, all p’s<0.001). We detected a positive CA for the 1-star agent (*b* = 0.08, *t*(609) = 3.32, p < 0.001).

FREE-CREDIBILITY BAYESIAN SIMULATIONS							
**Name**	**Estimate**	**SE**	**tStat**	**DF**	**pValue**	**Lower**	**Upper**
(**Intercept**)	0.08	0.02	3.01	609	0.002	0.03	0.13
**AGENT(2-STAR**)	0.11	0.03	3.26	609	0.001	0.05	0.18
**AGENT(3-STAR**)	0.61	0.03	20.05	609	<0.001	0.54	0.67
**Any effect of AGENT:** *F*(2,609) = 172.43, p < 0.001							
**AGENT(3-STAR) vs. AGENT(2-STAR):** *b* = 0.5, *F*(1,609) = 201.64, p < 0.001							

#### CA, feedback valence, and feedback credibility

In this section, we provide full result tables for mixed-effects models used in the sections ‘Individuals show a positivity bias in learning’ and ‘Positivity bias increases for sources of limited credibility’; and Figure 5a, b. The Wilkinson’s notation of the model is:

$$\displaystyle CA{\sim }VALENCE\mathrm{*}\left (AGENT_{2\mathrm{-}star}\mathrm{+}AGENT_{3\mathrm{-}star}\right)\mathrm{+}\left (1\mathrm{|}participant\right)$$

**Appendix 3—table 9.** Mixed-effects linear regression model regressing CA on feedback-valence and agent-credibility, based on credibility-valence-CA fits of participants’ data. We found an overall positive valence effect (*b* = 0.64, *F*(1,1218) = 37.39, p < 0.001), with no interactions with agent-credibility (*F*(2,1218) = 0.12, p = 0.88).

PARTICIPANTS DATA							
**Name**	**Estimate**	**SE**	**tStat**	**DF**	**pValue**	**Lower**	**Upper**
(**Intercept**)	0.29	0.09	3.19	1218	0.001	0.11	0.47
**VALENCE**	0.65	0.18	3.56	1218	<0.001	0.29	1
**AGENT(2-STAR**)	0.21	0.13	1.61	1218	0.11	–0.05	0.46
**AGENT(3-STAR**)	1.25	0.13	9.76	1218	<0.001	1	1.5
**VALENCE:AGENT(2-STAR**)	0.05	0.26	0.21	1218	0.83	–0.45	0.56
**VALENCE:AGENT(3-STAR**)	–0.07	0.26	–0.29	1218	0.77	–0.58	0.43
**Average valence effect**: *b* = 0.64, *F*(1,1218) = 37.39, *p* < 0.001							
**Any interaction**: *F*(2,1218)=0.12, p=0.88							
**Effect of negative feedback 1-star**: *b* = −0.03, *F*(1,1218) = 0.07, p = 0.79							
**Effect of positive feedback 1-star**: *b* = 0.61, *F*(1,1218) = 22.81, p < 0.001							
**Effect of negative feedback 2-star**: *b* = 0.14, *F*(1,1218) = 1.28, p = 0.25							
**Effect of positive feedback 2-star**: *b* = 0.85, *F*(1,1218) = 43.5, p < 0.001							
**Effect of negative feedback 3-star**: *b* = 1.25, *F*(1,1218) = 95.7, p = <0.001							
**Effect of positive feedback 3-star**: *b* = 1.83, *F*(1,1218) = 203.1, p < 0.001							

**Appendix 3—table 10.** Mixed-effects linear regression model regressing CA on feedback-valence and agent-credibility, based on credibility-valence-CA fits of instructed-credibility Bayesian model simulations. We found an overall negative valence effect (*b* = −0.54, *F*(1,1218) = 101.87, p < 0.001), with no interactions with agent-credibility (*F*(2,1218) = 0.02, *p* = 0.98).

INSTRUCTED-CREDIBILITY BAYESIAN SIMULATIONS							
**Name**	**Estimate**	**SE**	**tStat**	**DF**	**pValue**	**Lower**	**Upper**
(**Intercept**)	0	0.05	–0.06	1218	0.95	–0.09	0.09
**VALENCE**	–0.5	0.09	–5.38	1218	<0.001	–0.68	–0.32
**AGENT(2-STAR**)	0.29	0.07	4.41	1218	<0.001	0.16	0.41
**AGENT(3-STAR**)	0.62	0.07	9.54	1218	<0.001	0.49	0.75
**VALENCE:AGENT(2-STAR**)	–0.04	0.13	–0.34	1218	0.73	–0.3	0.21
**VALENCE:AGENT(3-STAR**)	–0.08	0.13	–0.6	1218	0.54	–0.33	0.18
**Average valence effect**: *b* = −0.54, *F*(1,1218) = 101.87, p < 0.001							
**Any interaction**: *F*(2,1218) = 0.02, p = 0.98							
**Effect of negative feedback 1-star**: *b* = 0.25, *F*(1,1218) = 14.17, p < 0.001							
**Effect of positive feedback 1-star**: *b* = −0.25, *F*(1,1218) = 14.78, p < 0.001							
**Effect of negative feedback 2-star**: *b* = 0.55, *F*(1,1218) = 72.47, p < 0.001							
**Effect of positive feedback 2-star**: *b* = 0.014, *F*(1,1218) = 0.04, p = 0.83							
**Effect of negative feedback 3-star**: *b* = 0.91, *F*(1,1218) = 193.45, p < 0.001							
**Effect of positive feedback 3-star**: *b* = 0.33, *F*(1,1218) = 25.91, p < 0.001							

**Appendix 3—table 11.** Mixed-effects linear regression model regressing CA on feedback-valence and agent-credibility, based on credibility-valence-CA fits of free-credibility Bayesian model simulations. We found an overall negative valence effect (*b* = −0.54, *F*(1,1218) = 98.91, p < 0.001), with no interactions with agent-credibility (*F*(2,1218)=0.06, *p*=0.94).

FREE-CREDIBILITY BAYESIAN SIMULATIONS							
**Name**	**Estimate**	**SE**	**tStat**	**DF**	**pValue**	**Lower**	**Upper**
(**Intercept**)	0.08	0.05	1.72	1218	0.09	–0.01	0.17
**VALENCE**	–0.51	0.09	–5.39	1218	<0.001	–0.7	–0.33
**AGENT(2-STAR**)	0.1	0.07	1.56	1218	0.12	–0.03	0.24
**AGENT(3-STAR**)	0.62	0.07	9.31	1218	<0.001	0.49	0.76
**VALENCE:AGENT(2-STAR**)	–0.02	0.13	–0.18	1218	0.85	–0.29	0.24
**VALENCE:AGENT(3-STAR**)	–0.07	0.13	–0.56	1218	0.58	–0.34	0.19
**Average valence effect**: b=−0.54, F(1,1218)=98.91, *P*<0.001							
**Any interaction**: *F*(2,1218)=0.06, *p*=0.94							
**Effect of negative feedback 1-star**: *b* = 0.34, *F*(1,1218) = 25.29, p < 0.001							
**Effect of positive feedback 1-star**: *b* = −0.17, *F*(1,1218) = 6.74, *p* = 0.010							
**Effect of negative feedback 2-star**: *b* = 0.45, *F*(1,1218) = 45.91, p < 0.001							
**Effect of positive feedback 2-star**: *b* = −0.08, *F*(1,1218) = 1.48, *p* = 0.22							
**Effect of negative feedback 3-star**: *b* = 1.00, *F*(1,1218) = 221.82, p < 0.001							
**Effect of positive feedback 3-star**: *b* = 0.41, *F*(1,1218) = 37.84, p < 0.001							

### Discovery study

#### Choice-repetition and feedback credibility

In this section, we provide full result tables for mixed-effects models used in Appendix 1—Credible feedback promotes greater learning and The effect of non-credible feedback and learning; and Appendix 1—figure 2a. The Wilkinson’s notation of the model is:

$$\displaystyle REPEAT{\sim }FEEDBACK*BETTER*\left (AGENT_{2-star}+AGENT_{3-star}+AGENT_{4-star}\right)+\left (1|participant\right)$$

**Appendix 3—table 12.** Mixed-effects binomial regression model regressing choice-repetition on feedback-valence, agent-credibility and better/worse choice from previous trial featuring the same bandit pair. Based on participants’ data. Feedback effect increased as a function of agent-credibility, and we found no significant feedback valence effect for the 1-star agent.

PARTICIPANTS’ DATA							
**Name**	**Estimate**	**SE**	**tStat**	**DF**	**pValue**	**Lower**	**Upper**
(**Intercept**)	1.87	0.09	19.89	2462	<0.001	1.68	2.05
**FEEDBACK**	–0.09	0.08	–1.21	2462	0.22	–0.24	0.06
**BETTER**	0.82	0.13	6.11	2462	<0.001	0.56	1.09
**AGENT(2-STAR**)	0.19	0.05	3.44	2462	<0.001	0.08	0.3
**AGENT(3-STAR**)	0.05	0.05	0.88	2462	0.37	–0.06	0.16
**AGENT(4-STAR**)	–0.39	0.06	–7.07	2462	<0.001	–0.5	–0.28
**FEEDBACK:BETTER**	–0.67	0.27	–2.51	2462	0.012	–1.2	–0.15
**FEEDBACK:AGENT(2-STAR**)	0.52	0.11	4.74	2462	<0.001	0.31	0.74
**BETTER:AGENT(2-STAR**)	–0.01	0.2	–0.04	2462	0.97	–0.39	0.38
**FEEDBACK:AGENT(3-STAR**)	1.24	0.11	11.25	2462	<0.001	1.03	1.46
**BETTER:AGENT(3-STAR**)	–0.27	0.2	–1.41	2462	0.16	–0.66	0.11
**FEEDBACK:AGENT(4-STAR**)	2.55	0.11	22.91	2462	<0.001	2.33	2.77
**BETTER:AGENT(4-STAR**)	–0.5	0.2	–2.56	2462	0.01	–0.88	–0.12
**FEEDBACK:BETTER:AGENT(2-STAR**)	0.71	0.39	1.8	2462	0.071	–0.06	1.48
**FEEDBACK:BETTER:AGENT(3-STAR**)	0.44	0.39	1.11	2462	0.27	–0.33	1.2
**FEEDBACK:BETTER:AGENT(4-STAR**)	0.24	0.39	0.62	2462	0.53	–0.52	1.01
**Overall feedback effect**: *b* = 1.02, *F*(1,2462) = 617.38, p <0.001							
**Feedback effect for AGENT(4-STAR**): *b* = 2.46, F(1,2462) = 911.81, p < 0.001							
**Feedback effect for AGENT(3-STAR**): b = 1.15, F(1,2462) = 206.19, p < 0.001							
**Feedback effect for AGENT(2-STAR**): b = 0.43, F(1,2462) = 28.84, p < 0.001							
**Any interaction**: *F*(3,2462) = 196.61, p < 0.001							
**FEEDBACK:AGENT(4-STAR) vs. FEEDBACK:AGENT(2-STAR**): *b* = 0.47, *F*(1,2462) = 322.88, p < 0.001							
**FEEDBACK:AGENT(4-STAR) vs. FEEDBACK:AGENT(3-STAR):** *b* = 1.21, *F*(1,2462) = 137.71, p < 0.001							
**FEEDBACK:AGENT(3-STAR) vs. FEEDBACK:AGENT(2-STAR):** *b* = 2.03, *F*(1,2462) = 40.54, p < 0.001							

#### CA and feedback credibility

In this section, we provide full result tables for mixed-effects models used in Appendix 1—Credible feedback promotes greater learning, Substantial deviations from Bayesian Learning, The effect of non-credible feedback and learnin; and Appendix 1—figure 2c. The Wilkinson’s notation of the model is:

$$\displaystyle CA{\sim }AGENT_{2-star}{+}AGENT_{3-star}{+}AGENT_{4-star}{+}\left (1{|}participant\right)$$

**Appendix 3—table 13.** Mixed-effects linear regression model regressing CA on agent-credibility, based on credibility-CA fits of participants’ data. CA increased as a function of agent-credibility. We found no evidence for significant CA for the 1-star agent.

PARTICIPANTS’ DATA							
**Name**	**Estimate**	**SE**	**tStat**	**DF**	**pValue**	**Lower**	**Upper**
(**Intercept**)	–0.13	0.13	–1.07	412	0.28	–0.38	0.11
**AGENT(2-STAR**)	0.5	0.17	3.03	412	0.003	0.18	0.83
**AGENT(3-STAR**)	1.27	0.17	7.68	412	<0.001	0.94	1.59
**AGENT(4-STAR**)	2.67	0.17	16.13	412	<0.002	2.34	2.99
**AGENT(3-STAR) vs. AGENT(2-STAR):** *b* = 0.77, *F*(1,412) = 21.58, p < 0.001							
**AGENT(4-STAR) vs. AGENT(3-STAR**): *b* = 1.4, F(1,412) = 71.40, p < 0.001							
**AGENT(4-STAR) vs. AGENT(2-STAR**): *b* = 2 .17, F(1,412) = 171.49, p < 0.001							

**Appendix 3—table 14.** Mixed-effects linear regression model regressing CA on agent-credibility, based on credibility-CA fits of instructed-credibility Bayesian model simulations. CA increased as a function of agent-credibility. Instructed-credibility Bayesian simulations do not predict significant CA for the 1-star agent.

INSTRUCTED-CREDIBILITY BAYESIAN SIMULATIONS							
**Name**	**Estimate**	**SE**	**tStat**	**DF**	**pValue**	**Lower**	**Upper**
(**Intercept**)	–0.02	0.05	–0.33	412	0.74	–0.11	0.08
**AGENT(2-STAR**)	0.51	0.05	10.33	412	<0.001	0.41	0.61
**AGENT(3-STAR**)	0.91	0.05	18.5	412	<0.001	0.81	1.01
**AGENT(4-STAR**)	1.4	0.05	28.5	412	<0.001	1.31	1.5
**Any difference across agents**: *F*(3,412) = 98.77, p < 0.001							
**AGENT(3-STAR) vs. AGENT(2-STAR**): *b* = 0.4, *F*(1,412) = 66.8, p < 0.001							
**AGENT(4-STAR) vs. AGENT(3-STAR**): *b* = 0.49, *F*(1,412) = 99.94, p < 0.001							
**AGENT(4-STAR) vs. AGENT(2-STAR**): *b* = 0.89, *F*(1,412) = 330.15, p < 0.001							

**Appendix 3—table 15.** Mixed-effects linear regression model regressing CA on agent-credibility, based on credibility-CA fits of free-credibility Bayesian model simulations. CA increased as a function of agent-credibility. Free-credibility Bayesian simulations do not predict significant CA for the 1-star agent.

FREE-CREDIBILITY BAYESIAN SIMULATIONS							
**Name**	**Estimate**	**SE**	**tStat**	**DF**	**pValue**	**Lower**	**Upper**
(**Intercept**)	–0.1	0.09	–1.12	412	0.26	–0.27	0.07
**AGENT(2-STAR**)	0.41	0.11	3.68	412	<0.001	0.19	0.63
**AGENT(3-STAR**)	0.95	0.11	8.48	412	<0.001	0.73	1.17
**AGENT(4-STAR**)	2.09	0.11	18.64	412	<0.002	1.87	2.31
**AGENT(3-STAR) vs. AGENT(2-STAR):** *b* = 0.54, *F*(1,412) = 22.97, p < 0.001							
**AGENT(4-STAR) vs. AGENT(3-STAR):** *b* = 1.14, *F*(1,412) = 103.24, p < 0.001							
**AGENT(4-STAR) vs. AGENT(2-STAR):** *b* = 1.68, *F*(1,412) = 223.60, p < 0.001							

#### CA, feedback valence, and feedback credibility

In this section, we provide full result tables for mixed-effects models used in Appendix 1—Sections 2.5; and Appendix 1—figure 3a, b. The Wilkinson’s notation of the model is:

$$\displaystyle CA{\sim }VALENCE*\left (AGENT_{2-star}{+}AGENT_{3-star}{+}AGENT_{4-star}\right)+\left (1{|}participant\right)$$

**Appendix 3—table 16.** Mixed-effects linear regression model regressing CA on feedback-valence and agent-credibility, based on credibility-valence-CA fits of participants’ data. We found an overall positive valence effect, which interacted with agent-credibility, such that the valence effect was two 2-star agent than for the 4-star agent. Moreover, we found a positive valence effect for the 1-star, 2-star, and 3-star agents, but not for the 4-star agent. Finally, the negative feedback from 1-star agent had a significant negative effect on CA, while positive feedback from the same agent had a significant positive effect.

PARTICIPANTS’ DATA							
**Name**	**Estimate**	**SE**	**tStat**	**DF**	**pValue**	**Lower**	**Upper**
(**Intercept**)	–0.05	0.16	–0.29	824	0.77	–0.36	0.27
**VALENCE**	0.94	0.29	3.24	824	0.001	0.37	1.51
**AGENT(2-STAR**)	0.5	0.21	2.44	824	0.015	0.1	0.9
**AGENT(3-STAR**)	1.39	0.21	6.76	824	<0.001	0.99	1.79
**AGENT(4-STAR**)	2.8	0.21	13.66	824	<0.001	2.4	3.21
**VALENCE:AGENT(2-STAR**)	0.66	0.41	1.6	824	0.11	–0.15	1.46
**VALENCE:AGENT(3-STAR**)	0.01	0.41	0.02	824	0.99	–0.8	0.81
**VALENCE:AGENT(4-STAR**)	–0.63	0.41	–1.54	824	0.12	–1.44	0.17
**Average valence effect**: *b* = 0.94, *F*(1,824) = 42.60, p < 0.001							
**Effect of negative feedback 1-star**: *b* = −0.51, *F*(1,824) = 3.89, p = 0.049							
**Effect of positive feedback 1-star**: *b* = 0.42, *F*(1,824) = 5.74, p = 0.017							
**Effect of negative feedback 2-star**: *b* = −0.34, *F*(1,824) = 2.53, p = 0.11							
**Effect of positive feedback 2-star**: *b* = 1.25, *F*(1,824) = 16.34, p < 0.001							
**Effect of negative feedback 3-star**: *b* = 0.86, *F*(1,824) = 37.77, p < 0.001							
**Effect of positive feedback 3-star**: *b* = 2.28, *F*(1,824) = 71.28, p < 0.001							
**Effect of negative feedback 4-star**: *b* = 2.60, *F*(1,824) = 146.55, p < 0.001							
**Effect of positive feedback 4-star**: *b* = 2.91, *F*(1,824) = 183.22, p < 0.001							
**Any interaction**: *F*(3,824) = 3.28, p = 0.02							
**Valence effect AGENT(2-STAR**): *b* = 1.60, *F*(1, 824) = 30.21, p < 0.001							
**Valence effect AGENT(3-STAR**): *b* = 0.94, *F*(1, 824) = 10.63, p = 0.001							
**Valence effect AGENT(4-STAR**): *b* = 0.31, *F*(1, 824) = 1.12, p = 0.29							
**Valence effect AGENT(2-STAR) vs. AGENT(3-STAR**): *b* = 0.65, *F*(1, 824) = 2.50, p = 0.11							
**Valence effect AGENT(2-STAR) vs. AGENT(4-STAR**): *b* = 1.29, *F*(1, 824) = 9.84, p = 0.002							
**Valence effect AGENT(3-STAR) vs. AGENT(4-STAR**): *b* = 0.64, *F*(1, 824) = 2.42, p = 0.12							

**Appendix 3—table 17.** Mixed-effects linear regression model regressing CA on feedback-valence and agent-credibility, based on credibility-valence-CA fits of instructed-credibility Bayesian model simulations. We found an overall negative valence effect, with no interactions with agent-credibility.

INSTRUCTED-CREDIBILITY BAYESIAN SIMULATIONS							
**Name**	**Estimate**	**SE**	**tStat**	**DF**	**pValue**	**Lower**	**Upper**
(**Intercept**)	–0.01	0.08	–0.13	824	0.89	–0.18	0.15
**VALENCE**	–0.41	0.16	–2.53	824	0.012	–0.72	–0.09
**AGENT(2-STAR**)	0.54	0.11	4.79	824	<0.001	0.32	0.77
**AGENT(3-STAR**)	0.98	0.11	8.59	824	<0.001	0.75	1.2
**AGENT(4-STAR**)	1.53	0.11	13.41	824	<0.001	1.3	1.75
**VALENCE:AGENT(2-STAR**)	–0.02	0.23	–0.11	824	0.91	–0.47	0.42
**VALENCE:AGENT(3-STAR**)	–0.11	0.23	–0.5	824	0.61	–0.56	0.33
**VALENCE:AGENT(4-STAR**)	–0.18	0.23	–0.77	824	0.44	–0.62	0.27
**Average valence effect**: *b* = −0.49, *F*(1,824) = 36.47, p < 0.001							
**Any interaction**: *F*(3,824) = 0.25 p = 0.85							

**Appendix 3—table 18.** Mixed-effects linear regression model regressing CA on feedback-valence and agent-credibility, based on credibility-valence-CA fits of free-credibility Bayesian model simulations. We found an overall negative valence effect, with no interactions with agent-credibility.

FREE-CREDIBILITY BAYESIAN SIMULATIONS							
**Name**	**Estimate**	**SE**	**tStat**	**DF**	**pValue**	**Lower**	**Upper**
(**Intercept**)	–0.1	0.1	–0.99	824	0.32	–0.31	0.1
**VALENCE**	–0.68	0.2	–3.37	824	<0.001	–1.07	–0.28
**AGENT(2-STAR**)	0.44	0.14	3.1	824	0.002	0.16	0.72
**AGENT(3-STAR**)	1.03	0.14	7.27	824	<0.001	0.75	1.31
**AGENT(4-STAR**)	2.28	0.14	16.13	824	<0.001	2.01	2.56
**VALENCE:AGENT(2-STAR**)	0.05	0.28	0.17	824	0.86	–0.51	0.6
**VALENCE:AGENT(3-STAR**)	–0.07	0.28	–0.25	824	0.8	–0.63	0.48
**VALENCE:AGENT(4-STAR**)	–0.1	0.28	–0.36	824	0.72	–0.66	0.45
**Average valence effect**: *b* = −0.71, *F*(1,824) = 49.8, p < 0.001							
**Any interaction**: *F*(3,824) = 0.11 p = 0.95							

## Appendix 4

### Additional analyses of model parameters main study

#### Main study

##### Comparison of empirical-aVBI and instructed-credibility Bayesian-aVBI

In this section, we provide full result tables for the sections ‘Individuals show a positivity bias in learning’ and ‘Positivity bias increases for sources of limited credibility’; and Figure 5a, b. For each individual and credibility level we calculated the absolute Valence Bias Index (aVBI), defined as the difference between the Credit Assignment for positive (CA^+^) and negative feedback (CA^−^).

**Appendix 4—table 1.** Statistics summarizing Wilcoxon test results comparing aVBI from participants with the one from instructed-credibility Bayesian simulations. Participants showed a greater aVBI than predicted by the instructed-credibility Bayesian model, for all levels of credibility.

Name	Median difference	*z*	pValue
**50% credibility**	1.34	5.20	<0.001
**75% credibility**	1.34	5.94	<0.001
**100% credibility**	1.10	5.87	<0.001

**Appendix 4—table 2.** Statistics summarizing Wilcoxon test results comparing aVBI from participants with the one from free-credibility Bayesian simulations. Participants showed a greater aVBI than predicted by the free-credibility Bayesian model, for all levels of credibility.

Name	Median difference	*z*	pValue
**50% credibility**	1.02	5.95	<0.001
**75% credibility**	1.21	6.13	<0.001
**100% credibility**	1.21	5.36	<0.001

#### rVBI for Bayesian models

In this section, we provide full result tables for the section ‘Positivity bias increases for sources of limited credibility’; and Figure 5c.

**Appendix 4—table 3.** Mixed-effects linear regression model regressing rVBIs on agent-credibility, based on credibility-valence-CA fits of instructed-credibility Bayesian model simulations. The instructed-credibility Bayesian model predicted a negative rVBI for all credibility levels, with an increase in rVBI for higher credibility levels.

INSTRUCTED-CREDIBILITY BAYESIAN SIMULATIONS							
Name	Estimate	SE	tStat	DF	pValue	Lower	Upper
(Intercept)	–0.20	0.06	–3.38	609	<0.001	–0.32	–0.08
AGENT(2-STAR)	0.07	0.03	2.24	609	0.026	0.01	0.14
AGENT(3-STAR)	0.10	0.03	2.91	609	0.04	0.03	0.16
**Average rVBI effect**: *b* = −0.14, *F*(1,609) = 6.49, p = 0.011							
**Any interaction**: *F*(2,609) = 4.64 p = 0.01							

**Appendix 4—table 4.** Mixed-effects linear regression model regressing rVBIs on agent-credibility, based on credibility-valence-CA fits of free-credibility Bayesian model simulations. We found a negative relative valence effect, with no interactions with agent-credibility.

FREE-CREDIBILITY BAYESIAN SIMULATIONS							
Name	Estimate	SE	tStat	DF	pValue	Lower	Upper
(Intercept)	–0.15	0.06	–2.64	609	0.008	–0.27	0.04
AGENT(2-STAR)	–0.03	0.03	–0.95	609	0.34	–0.09	0.03
AGENT(3-STAR)	–0.02	0.03	–0.67	609	0.50	–0.08	0.04
**Average rVBI effect**: *b* = −0.17, *F*(1,609) = 9.55, p = 0.002							
**Any interaction**: *F*(2,609) = 0.48 p = 0.63							

### Discovery study

#### Comparison of empirical- and Bayesian-aVBI

In this section, we provide full result tables for Appendix 1—Individuals show a positivity bias in learning, particularly for sources of limited credibility; and Appendix 1—figure 3a, b.

**Appendix 4—table 5.** Statistics summarizing Wilcoxon test results comparing aVBI from participants with the one from instructed-credibility Bayesian simulations. Participants showed a greater aVBI than predicted by the instructed-credibility Bayesian model, for all levels of credibility.

INSTRUCTED-CREDIBILITY BAYESIAN SIMULATIONS			
Name	Median difference	*z*	pValue
50% credibility	1.04	3.63	<0.001
70% credibility	1.58	5.27	<0.001
85% credibility	1.49	4.70	<0.001
100% credibility	0.93	2.82	0.005

**Appendix 4—table 6.** Statistics summarizing Wilcoxon test results comparing aVBI from participants with the one from free-credibility Bayesian simulations. Participants showed a greater aVBI than predicted by the free-credibility Bayesian model, for all levels of credibility.

FREE-CREDIBILITY BAYESIAN SIMULATIONS			
Name	Median difference	*z*	pValue
50% credibility	1.43	3.99	<0.001
70% credibility	1.70	5.44	<0.001
85% credibility	1.59	4.78	<0.001
100% credibility	1.06	3.06	0.002

#### rVBI for Bayesian models

In this section, we provide full result tables for Appendix 1—Individuals show a positivity bias in learning, particularly for sources of limited credibility and Appendix 1—figure 3c.

**Appendix 4—table 7.** Mixed-effects linear regression model regressing rVBIs on agent-credibility, based on credibility-valence CA fits of instructed-credibility Bayesian model simulations. We found no relative valence effect, with no interactions with agent-credibility.

INSTRUCTED-CREDIBILITY BAYESIAN SIMULATIONS							
Name	Estimate	SE	tStat	DF	pValue	Lower	Upper
(Intercept)	–0.06	0.08	–0.78	412	0.44	–0.21	0.092
AGENT(2-STAR)	–0.05	0.04	–1.26	412	0.21	–0.13	0.03
AGENT(3-STAR)	–0.02	0.04	–0.44	412	0.66	–0.10	0.06
AGENT(4-STAR)	–0.03	0.04	–0.78	412	0.44	–0.11	0.05
**Average rVBI effect**: *b* = −0.085, *F*(1,412) = 1.32, p = 0.25							
**Any interaction**: *F*(3,412) = 0.57 p = 0.64							

**Appendix 4—table 8.** Mixed-effects linear regression model regressing rVBIs on agent-credibility, based on credibility-valence CA fits of free-credibility Bayesian model simulations. We found no relative valence effect, with no interactions with agent-credibility.

FREE-CREDIBILITY BAYESIAN SIMULATIONS							
Name	Estimate	SE	tStat	DF	pValue	Lower	Upper
(Intercept)	–0.02	0.08	–0.21	412	0.83	–0.17	0.14
AGENT(2-STAR)	–0.02	0.04	–0.59	412	0.55	–0.10	0.05
AGENT(3-STAR)	0.007	0.04	0.19	412	0.85	–0.07	0.08
AGENT(4-STAR)	–0.02	0.04	–0.43	412	0.66	–0.10	0.06
**Average rVBI effect**: *b* = −0.024, *F*(1,412) = 0.11, p = 0.74							
**Any interaction**: *F*(3,412) = 0.26, p = 0.85							

### Distribution of fitted parameters

#### Main study

**Appendix 4—table 9.** fitted parameters from participants for our main CA models. Values represent the group mean (sd).

	Credibility-CA	Credibility-calence CA	Truth CA
$CA^{-}_{0.5}$	0.23 (0.41)	–0.01 (1.55)	0.23 (0.42)
$CA^{+}_{0.5}$		0.59 (1.87)	
$CA^{-}_{0.75}$	0.45 (0.50)	0.17 (1.48)	0.39 (0.51)
$CA^{+}_{0.75}$		0.81 (1.85)	
$CA^{-}_{1}$	1.47 (1.07)	1.28 (1.70)	1.35 (1.10)
$CA^{+}_{1}$		1.80 (2.42)	
*TB*			0.21 (0.94)
*f_Q_*	0.25 (0.26)	0.27 (0.28)	0.25 (0.26)
*PERS*	0.63 (0.36)	0.48 (1.44)	0.63 (0.38)
*f_P_*	0.16 (0.19)	0.28 (0.34)	0.16 (0.19)

**Appendix 4—table 10.** Fitted parameters from participants for our main Bayesian models. Values represent the group mean (sd).

	Instructed-credibility	Free-credibility
$\beta $	4.26 (3.02)	4.96 (3.34)
$C_{0.5}$		0.60 (0.23)
$C_{0.75}$		0.65 (0.23)

#### Discovery study

**Appendix 4—table 11.** fitted parameters from participants for our main CA models. Values represent the group mean (sd).

	Credibility-CA	Credibility-valence CA	Truth CA
$CA^{-}_{0.5}$	–0.13 (0.81)	–0.50 (1.88)	–0.24 (1.47)
$CA^{+}_{0.5}$		0.39 (2.14)	
$CA^{-}_{0.7}$	0.37 (0.70)	–0.31 (1.87)	–0.06 (1.69)
$CA^{+}_{0.7}$		1.23 (2.29)	
$CA^{-}_{0.85}$	1.14 (0.99)	0.90 (1.67)	0.02 (1.72)
$CA^{+}_{0.85}$		1.78 (2.21)	
$CA^{-}_{1}$	2.56 (2.10)	2.62 (2.51)	0.98 (2.72)
$CA^{+}_{1}$		2.90 (2.77)	
*TB*			3.18 (3.26)
*f_Q_*	0.26 (0.21)	0.23 (0.23)	0.47 (0.33)
*PERS*	1.24 (0.95)	0.87 (1.66)	2.43 (1.55)
*f*_*P*_	0.51 (0.33)	0.63 (0.41)	0.43 (0.28)

**Appendix 4—table 12.** fitted parameters from participants for our main Bayesian models. Values represent the group mean (sd).

	Instructed-credibility	Free-credibility
$\beta $	9.70 (5.25)	13.11 (7.14)
$C_{0.5}$		0.50 (0.20)
$C_{0.7}$		0.59 (0.20)
$C_{0.85}$		0.76 (0.18)

## Appendix 5

### Contrast effects for contexts featuring a different bandit

Given that we observed a contrast effect when both the learning and the immediately preceding ‘context trial’ involved the same pair of bandits, we next investigated whether this effect persisted when the context trial featured a different bandit pair—a situation where the context would be irrelevant to the current learning. Again, we used in a binomial mixed-effects model, regressing choice-repetition on feedback valence in the learning trial and the feedback agent in the context trial. This analysis included only learning trials featuring the 3-star agent, and context trials featuring a different bandit pair than the learning trial (Appendix 5—figure 1a). We found no significant evidence of an interaction between feedback valence and contextual credibility (*F*(2,2364) = 0.21, p = 0.81) (Appendix 5—figure 1b). This null result was consistent with the range of outcomes predicted by our main computational models (Appendix 5—figure 1c).

we aimed to formally compare the influence of two types of contextual trials: those featuring the same bandit pair as the learning trial versus those featuring a different pair. To achieve this, we extended our mixed-effects model by incorporating a new predictor variable, ‘*CONTEXT_TYPE*’ which coded whether the contextual trial involved the same bandit pair (coded as –0.5) or a different bandit pair (+0.5) compared to the learning trial. The Wilkinson notation for this expanded mixed-effects model is:

$$\displaystyle \begin{array}{ll}REPEAT{\sim }CONTEXT{\_ }TYPE{*}FEEDBACK{*}\left (CONTEXT_{2-star}{+}CONTEXT_{3{-}star}\right)\\ {+}BETTER{+}\left (1|participant\right)\end{array}$$

This expanded model revealed a significant three-way interaction between feedback valence, contextual credibility, and context type (F(2,4451)=7.71, *P*<0.001). Interpreting this interaction, we found a two-way interaction between context-source and feedback valence when the context was the same (F(2,4451)=12.03, *P*<0.001), but not when context was different (F(2,4451)=0.23, *P*=0.79). Further interpreting the double feedback-valence * context-source interaction (for the same context) we obtained the same conclusions as reported in the main text.

## Appendix 6

### Positivity bias results cannot be explained by a pure perseveration

#### Main study

Previous research has suggested it may be challenging to dissociate between a feedback-valence positivity bias and perseveration (i.e., a tendency to repeat previous choices regardless of outcome). While our Credit Assignment (CA) models already include a perseveration mechanism to account for this, this control may not be perfect. We thus conducted several tests to examine if our positivity-bias related results could be accounted for by perseveration.

First we examined whether our Bayesian models, augmented by a perseveration mechanism (as in our CA model) can generate predictions similar to our empirical results. We repeated our cross-fitting procedure to these extended Bayesian models. To briefly recap, this involved fitting participant behaviour with them, generating synthetic datasets based on the resulting maximum likelihood (ML) parameters, and then fitting these simulated datasets with our credibility-valence CA model (which is designed to detect positivity bias). This test revealed that adding perseveration to our Bayesian models did not predict a positivity bias in learning. In absolute terms there was a small negativity bias (instructed-credibility Bayesian: b=−0.19, F(1,1218)=17.78, *P*<0.001, Appendix 6—figure 1a, b; free-credibility Bayesian: b=−0.17, F(1,1218)=13.74, *P*<0.001, Appendix 6—figure 1d, e). In relative terms we detected no valence related bias (instructed-credibility Bayesian: b=−0.034, F(1,609)=0.45, *P*=0.50, Appendix 5—figure 1c; free-credibility Bayesian: b=−0.04, F(1,609)=0.51, *P*=0.47, Appendix 6—figure 1f). More critically, these simulations also did not predict a change in the level of positivity bias as a function of feedback credibility, neither at an absolute level (instructed-credibility Bayesian: F(2,1218)=0.024, *P*=0.98, Appendix 6—figure 1b; free-credibility Bayesian: F(2,1218)=0.008, *P*=0.99, Appendix 6—figure 1e), nor at a relative level (instructed-credibility Bayesian: F(2,609)=1.57, *P*=0.21, Appendix 6—figure 1c; free-credibility Bayesian: F(2,609)=0.13, *P*=0.88, Appendix 6—figure 1f). The upshot is that our positivity-bias findings cannot be accounted for by our Bayesian models even when these are augmented with perseveration.

However, it is still possible that empirical CA parameters from our credibility-valence model (reported in main text Figure 5) were distorted, absorbing variance from a perseveration. To address this, we took a ‘devil’s advocate’ approach testing the assumption that CA parameters are not *truly* affected by feedback valance and that there is only perseveration in our data. Towards that goal, we simulated data using our credibility-CA model (which includes perseveration but does not contain a valence bias in its learning mechanism) and then fitted these synthetic datasets using our credibility-valence CA model to see if the observed positivity bias could be explained by perseveration alone. Specifically, we generated 101 ‘group-level’ synthetic datasets (each including one simulation for each participant, based on their empirical ML parameters), and fitted each dataset with our credibility-valence CA model. We then analysed the resulting ML parameters in each dataset using the same mixed-effects models as described in the main text, examining the distribution of effects of interest across these simulated datasets. Comparing these simulation results to the data from participants revealed a nuanced picture. While the positivity bias observed in participants is within the range predicted by a pure perseveration account when measured in absolute terms (Appendix 6—figure 2a), it is much higher than predicted by pure perseveration when measured relative to the overall level of learning (Appendix 6—figure 2c). Interestingly, a pure perseveration account predicted an amplification of the relative positivity bias under low (compared to full) credibility (with the two rightmost histograms in Appendix 6—figure 2d falling in the positive range). However, the magnitude of this effect was significantly smaller than the empirical effect (as the bulk of these same histograms lies below the green points). Moreover, this account predicted a negative amplification (i.e., attenuation) of an absolute positivity bias, which was again significantly smaller than the empirical effect (see corresponding histograms in S24b). This pattern raises an intriguing possibility that perseveration may, at least partially, mask a true amplification of absolute positivity bias.

### Discovery study

We then replicated these analyses in our discovery study to confirm our findings. We again checked whether extended versions of the Bayesian models (including perseveration) predicted the positivity bias results observed. Our cross-fitting procedure showed that the instructed-credibility Bayesian model with perseveration did predict a positivity bias for all credibility levels in this discovery study, both when measured in absolute terms [50% credibility (b=1.74,t(824)=6.15), 70% credibility (b=2.00,F(1,824)=49.98), 85% credibility (b=1.81,F(1,824)=40.78), 100% credibility (b=2.42,F(1,824)=72.50), all p’s<0.001], and in relative terms [50% credibility (b=0.25,t(412)=3.44), 70% credibility (b=0.31,F(1,412)=17.72), 85% credibility (b=0.34,F(1,412)=21.06), 100% credibility (b=0.42,F(1,412)=31.24), all p’s<0.001]. However, importantly, these simulations did not reveal a significant change in the level of positivity bias as a function of feedback credibility, neither at an absolute level (F(3,412)=1.43,*P*=0.24), nor at a relative level (F(3,412)=2.06,*P*=0.13) (Appendix 6—figure 3a–c). Numerically, the trend was towards an increasing (rather than decreasing) positivity bias as a function of credibility. In contrast, simulations of the free-credibility Bayesian model (with perseveration) predicted a slight negativity bias when measured in absolute terms (b=−0.35,F(1,824)=5.14,*P*=0.024), and no valence bias when measured relative to the overall degree of learning (b=0.05,F(1,412)=0.55,*P*=0.46). Crucially, this model also did not predict a change in the level of positivity bias as a function of feedback credibility, neither at an absolute level (F(3,824)=0.27,*P*=0.77), nor at a relative level (F(3,412)=0.76,*P*=0.47) (Appendix 6—figure 3d–f).

As in our main study, we next assessed whether our credibility-CA model (which includes perseveration but no valence bias) predicted the positivity bias results observed in participants in the discovery study. This analysis revealed that the average positivity bias in participants is higher than predicted by a pure perseveration account, both when measured in absolute terms (Appendix 6—figure 4a) and in relative terms (Appendix 6—figure 4c). Specifically, only the aVBI for the 70% credibility agent was above what a perseveration account would predict, while the rVBI for all agents except the completely credible one exceeded that threshold. Furthermore, the inflation in positivity bias for lower credibility feedback (compared to the 100% credibility agent) is significantly higher in participants than would be predicted by a pure perseveration account, in both absolute (Appendix 6—figure 4b) and relative (Appendix 6—figure 4d) terms.

Together, these results show that the general positivity bias observed in participants could be predicted by an instructed-credibility Bayesian model with perseveration, or by a CA model with perseveration. Moreover, we find that these two models can predict a positivity bias for the 50% credibility agent, raising a concern that our positivity bias findings for this source may be an artefact of not-fully controlled for perseveration. However, the credibility modulation of this positivity bias, where the bias is amplified for lower credibility feedback, is consistently not predicted by perseveration alone, regardless of whether perseveration is incorporated into a Bayesian or a CA model. This finding suggests that participants are genuinely modulating their learning based on feedback credibility, and that this modulation is not merely an artifact of choice perseveration.

### Truth inference is still detected when controlling for valence bias

Given that participants frequently select bandits that are, on average, mostly rewarding, it is reasonable to assume that positive feedback is more likely to be objectively true than negative feedback. This raises a question if the ‘truth inference’ effect we observed in participants might simply be an alternative description of a positivity bias in learning. To directly test this idea, we extended our Truth-CA model to explicitly account for a valence bias in credit assignment. This extended model features separate CA parameters for positive and negative feedback for each agent. When we fitted this new model to participant behaviour, it still revealed a significant truth bonus in both the main study (Wilkoxon’s signrank test: median = 0.09, *z*(202) = 2.12, p = 0.034; Appendix 6—figure 5a) and the discovery study (median = 3.52, *z*(102) = 7.86, p < 0.001; Appendix 6—figure 5c). Moreover, in the main study, this truth bonus remained significantly higher than what was predicted by all the alternative models, with the exception of the instructed-credibility bayesian model (Appendix 6—figure 5b). In the discovery study, the truth bonus was significantly higher than what was predicted by all the alternative models (Appendix 6—figure 5d).

## Appendix 7

### Derivation of posterior update for Bayesian model

In the Bayesian models, the beliefs about each bandit were represented by density distribution *g*(*p*) over the probability *p* a bandit will provide a true reward. In a given trial *n*, after reward-feedback was provided, the distribution *for* the chosen bandit was updated based on Bayes' rule considering the agent’s feedback in the current trial (*f_n_*), its associated credibility (*C_n_*), and the history of the bandit prior to trial *n* (*H*_1,2,…_*_n_*_-1_). This update was calculated as (note proportionality omits terms that are independent of *p* and $r_{n}$ denotes the true, latent, choice outcome on trial *n*):

$$\displaystyle \begin{array}{ll} g\left (p|H_{1,2,\ldots n}\right)& =g\left (p|f_{n},C_{n},H_{1,2,\ldots n-1}\right)\propto Prob\left (f_{n}|p,C_{n},H_{1,2,\ldots n-1}\right)g\left (p|C_{n},H_{1,2,\ldots n-1}\right) \\ &{=}g\left (p|H_{1,2,\ldots n-1}\right)\sum _{r_{n}=0}^{1}Prob\left (r_{n},f_{n}|p,C_{n},H_{1,2,\ldots n{-}1}\right) \\ &{=}g\left (p|H_{1,2,\ldots n-1}\right)\sum _{r_{n}{=}0}^{1}Prob\left (f_{n}|r_{n},p,C_{n},H_{1,2,\ldots n-1}\right)Prob\left (r_{n}|p,C_{n},H_{1,2,\ldots n{-}1}\right) \\ &{=}g\left (p|H_{1,2,\ldots n-1}\right)\sum _{r_{n}{=}0}^{1}Prob\left (f_{n}|r_{n},C_{n}\right)Prob\left (r_{n}|p\right){=}g\left (p|H_{1,2,\ldots n-1}\right)\\ &\left (C_{n}{*}1_{f{=}1} +\left (1-C_{n}\right){*}1_{f=0}\right)p{+}\left (C_{n}{*}1_{f{=}0}{+}\left (1{-}C_{n}\right){*}1_{f=1}\right)\left (1{-}p\right) \\ &{=}g\left (p|H_{1,2,\ldots n-1}\right)\left [\left (C_{n}p+\left (1-C_{n}\right)\left (1-p\right)\right)1_{f=1}{+}\left (C_{n}\left (1-p\right){+}\left (1-C_{n}\right)p\right)1_{f=0}\right ]\end{array}$$

This was followed with normalisation to compensate for the proportionality in the derivation above

$$\displaystyle g\left (p| H_{1,2,\ldots n}\right)\leftarrow \frac{g\left (p| H_{1,2,\ldots n}\right)}{\int _{0}^{1}g\left (p| H_{1,2,\ldots n}\right)dp}$$

For the forgone trial *n* bandit we have $g\left (p|H_{1,2,\ldots n}\right){=}g\left (p|H_{1,2,\ldots n-1}\right)$.
