# Supplementary material for: Disinformation elicits learning biases
Source: eLife. 2026 Jun 26;14:RP106073. doi: 10.7554/eLife.106073 (PMC13309127; doi:10.7554/eLife.106073)
Supplement: Peer review history

# Editor report: eLife Assessment

**DOI:** 10.7554/eLife.106073.4.sa0

**Authors:** Andreea Oliviana Diaconescu

**Affiliations:** University of Toronto Canada

**Keywords:** Compelling, Important

This study provides an **important** advance in credibility-based learning research by demonstrating how feedback reliability can shape reward learning biases within a carefully controlled bandit task. The strength of the main findings, namely greater learning from credible feedback and robust computational modeling supported by strong parameter recovery and cross-fitting analyses, is **compelling**. The integration of reinforcement learning and Bayesian benchmark models is methodologically rigorous and well-executed, yielding reliable and interpretable results. The revised manuscript shows clear improvements in theoretical framing and transparency regarding limitations. While additional work is warranted to confirm the role of disinformation in amplifying positivity bias and to explore symptom-related variability or richer Bayesian comparators, this paper represents a high-quality and impactful contribution to the study of learning under uncertainty and misinformation.

# Referee report: Reviewer #1 (Public review)

**DOI:** 10.7554/eLife.106073.4.sa1

This is a well-designed and very interesting study examining the impact of imprecise feedback on outcomes on decision-making. I think this is an important addition to the literature and the results here, which provide a computational account of several decision-making biases, are insightful and interesting.

I do not believe I have substantive concerns related to the actual results presented; my concerns are more related to the framing of some of the work. My main concern is regarding the assertion that the results prove that non-normative and non-Bayesian learning is taking place. I agree with the authors that their results demonstrate that people will make decisions in ways that demonstrate deviations from what would be optimal for maximizing reward in their task under a strict application of Bayes rule. I also agree that they have built reinforcement learning models which do a good job of accounting for the observed behavior. However, the Bayesian models included are rather simple- per the author descriptions, applications of Bayes' rule with either fixed or learned credibility for the feedback agents. In contrast, several versions of the RL models are used, each modified to account for different possible biases. However more complex Bayes-based models exist, notably active inference but even the hierarchical gaussian filter. These formalisms are able to accommodate more complex behavior, such as affect and habits, which might make them more competitive with RL models. I think it is entirely fair to say that these results demonstrate deviations from an idealized and strict Bayesian context; however, the equivalence here of Bayesian and normative is I think misleading or at least requires better justification/explanation. This is because a great deal of work has been done to show that Bayes optimal models can generate behavior or other outcomes that are clearly not optimal to an observer within a given context (consider hallucinations for example) but which make sense in the context of how the model is constructed as well as the priors and desired states the model is given.

As such, I would recommend that the language be adjusted to carefully define what is meant by normative and Bayesian and to recognize that work that is clearly Bayesian could potentially still be competitive with RL models if implemented to model this task. An even better approach would be to directly use one of these more complex modelling approaches, such as active inference, as the comparator to the RL models, though I would understand if the authors would want this to be a subject for future work.

Abstract:

The abstract is lacking in some detail about the experiments done, but this may be a limitation of the required word count? If word count is not an issue, I would recommend adding details of the experiments done and the results.One comment is that there is an appeal to normative learning patterns, but this suggests that learning patterns have a fixed optimal nature, which may not be true in cases where the purpose of the learning (e.g. to confirm the feeling of safety of being in an in-group) may not be about learning accurately to maximize reward. This can be accommodated in a Bayesian framework by modelling priors and desired outcomes. As such the central premise that biased learning is inherently non-normative or non-Bayesian I think would require more justification. This is true in the introduction as well.

Introduction:

As noted above the conceptualization of Bayesian learning being equivalent to normative learning I think requires either further justification. Bayesian belief updating can be biased an non-optimal from an observer perspective, while being optimal within the agent doing the updating if the priors/desired outcomes are set up to advantage these "non-optimal" modes of decision making.

Results:

I wonder why the agent was presented before the choice - since the agent is only relevant to the feedback after the choice is made. I wonder if that might have induced any false association between the agent identity and the choice itself. This is by no means a critical point but would be interesting to get the authors' thoughts.

The finding that positive feedback increases learning is one that has been shown before and depends on valence, as the authors note. They expanded their reinforcement learning model to include valence; but they did not modify the Bayesian model in a similar manner. This lack of a valence or recency effect might also explain the failure of the Bayesian models in the preceding section where the contrast effect is discussed. It is not unreasonable to imagine that if humans do employ Bayesian reasoning that this reasoning system has had parameters tuned based on the real world, where recency of information does matter; affect has also been shown to be incorporable into Bayesian information processing (see the work by Hesp on affective charge and the large body of work by Ryan Smith). It may be that the Bayesian models chosen here require further complexity to capture the situation, just like some of the biases required updates to the RL models. This complexity, rather than being arbitrary, may be well justified by decision-making in the real world.

The methods mention several symptom scales- it would be interesting to have the results of these and any interesting correlations noted. It is possible that some of individual variability here could be related to these symptoms, which could introduce precision parameter changes in a Bayesian context and things like reward sensitivity changes in an RL context.

Discussion:

(For discussion, not a specific comment on this paper): One wonders also about participant beliefs about the experiment or the intent of the experimenters. I have often had participants tell me they were trying to "figure out" a task or find patterns even when this was not part of the experiment. This is not specific to this paper, but it may be relevant in the future to try and model participant beliefs about the experiment especially in the context of disinformation, when they might be primed to try and "figure things out".

As a general comment, in the active inference literature, there has been discussion of state-dependent actions, or "habits", which are learned in order to help agents more rapidly make decisions, based on previous learning. It is also possible that what is being observed is that these habits are at play, and that they represent the cognitive biases. This is likely especially true given, as the authors note, the high cognitive load of the task. It is true that this would mean that full-force Bayesian inference is not being used in each trial, or in each experience an agent might have in the world, but this is likely adaptive on the longer timescale of things, considering resource requirements. I think in this case you could argue that we have a departure from "normative" learning, but that is not necessarily a departure from any possible Bayesian framework, since these biases could potentially be modified by the agent or eschewed in favor of more expensive full-on Bayesian learning when warranted.Indeed in their discussion on the strategy of amplifying credible news sources to drown out low-credibility sources, the authors hint to the possibility of longer term strategies that may produce optimal outcomes in some contexts, but which were not necessarily appropriate to this task. As such, the performance on this task- and the consideration of true departure from Bayesian processing- should be considered in this wider context.Another thing to consider is that Bayesian inference is occurring, but that priors present going in produce the biases, or these biases arise from another source, for example factoring in epistemic value over rewards when the actual reward is not large. This again would be covered under an active inference approach, depending on how the priors are tuned. Indeed, given the benefit of social cohesion in an evolutionary perspective, some of these "biases" may be the result of adaptation. For example, it might be better to amplify people's good qualities and minimize their bad qualities in order to make it easier to interact with them; this entails a cost (in this case, not adequately learning from feedback and potentially losing out sometimes), but may fulfill a greater imperative (improved cooperation on things that matter). Given the right priors/desired states, this could still be a Bayes-optimal inference at a social level and as such may be ingrained as a habit which requires effort to break at the individual level during a task such as this.

The authors note that this task does not relate to "emotional engagement" or "deep, identity-related, issues". While I agree that this is likely mostly true, it is also possible that just being told one is being lied to might elicit an emotional response that could bias responses, even if this is a weak response.

Comments on first revisions:

In their updated version the authors have made some edits to address my concerns regarding the framing of the 'normative' Bayesian model, clarifying that they utilized a simple Bayesian model which is intended to adhere in an idealized manner to the intended task structure, though further simulations would have been ideal.

The authors, however, did not take my recommendation to explore the symptoms in the symptom scales they collected as being a potential source of variability. They note that these were for hypothesis generation and were exploratory, fair enough, but this study is not small and there should have been sufficient sample size for a very reasonable analysis looking at symptom scores.

However, overall the toned-down claims and clarifications of intent are adequate responses to my previous review.

Comments on second revisions:

While I believe an exploration of symptom scores would have been a valuable addition, this is not required for the purpose of the paper, and as such, I have no further comments.

# Referee report: Reviewer #2 (Public review)

**DOI:** 10.7554/eLife.106073.4.sa2

This important paper studies the problem of learning from feedback given by sources of varying credibility. The convincing combination of experiment and computational modeling helps to pin down properties of learning, while opening unresolved questions for future research.

Summary:

This paper studies the problem of learning from feedback given by sources of varying credibility. Two bandit-style experiments are conducted in which feedback is provided with uncertainty, but from known sources. Bayesian benchmarks are provided to assess normative facets of learning, and alternative credit assignment models are fit for comparison. Some aspects of normativity appear, in addition to possible deviations such as asymmetric updating from positive and negative outcomes.

Strengths:

The paper tackles an important topic, with a relatively clean cognitive perspective. The construction of the experiment enables the use of computational modeling. This helps to pinpoint quantitatively the properties of learning and formally evaluate their impact and importance. The analyses are generally sensible, and advanced parameter recovery analyses (including cross-fitting procedure) provide confidence in the model estimation and comparison. The authors have very thoroughly revised the paper in response to previous comments.

Weaknesses:

The authors acknowledge the potential for cognitive load and the interleaved task structure to play a meaningful role in the results, though leave this for future work. This is entirely reasonable, but remains a limitation in our ability to generalize the results. Broadly, some of the results obtained in cases where the extent of generalization is not always addressed and remains uncertain.

# Referee report: Reviewer #3 (Public review)

**DOI:** 10.7554/eLife.106073.4.sa3

Summary

This paper investigates how disinformation affects reward learning processes in the context of a two-armed bandit task, where feedback is provided by agents with varying reliability (with lying probability explicitly instructed). They find that people learn more from credible sources, but also deviate systematically from optimal Bayesian learning: They learned from uninformative random feedback and updated too quickly from fully credible feedback (especially following low-credibility feedback). People also appeared to learn more from positive feedback and there is tentative evidence that this bias is exacerbated for less credible feedback.

Overall, this study highlights how misinformation could distort basic reward learning processes, without appeal to higher order social constructs like identity.

Strengths- The experimental design is simple and well-controlled; in particular, it isolates basic learning processes by abstracting away from social context- Modeling and statistics meet or exceed standards of rigor- Limitations are acknowledged where appropriate, especially those regarding external validity and challenges in dissociating positivity bias from perseveration- The comparison model, Bayes with biased credibility estimates, is strong; deviations are much more compelling than e.g. a purely optimal model- The conclusions are of substantial interest from both a theoretical and applied perspective

Weaknesses

The authors have done a great job addressing my concerns with the two previous submission. The one issue that they were not able to truly address is the challenge of dissociating positivity bias from perseveration; this challenge weakens evidence for the conclusion that less credible feedback yields a stronger positivity bias. However, the authors have clearly acknowledged this limitation and tempered their conclusions accordingly. Furthermore, the supplementary analyses on this point are suggestive (if not fully conclusive) and do a better job of at least trying to address the confound than most work on positivity/confirmation bias.

I include my previous review describing the challenge in more detail for reference. I encourage interested readers to see the author response as well. It has convinced me that this weakness is not a reflection of the work, but is instead a fundamental challenge for research on positivity bias.

Absolute or relative positivity bias?

The conclusion of greater positivity bias for lower credible feedback (Fig 5) hinges on the specific way in which positivity bias is defined. Specifically, we only see the effect when normalizing the difference in sensitivity to positive vs. negative feedback by the sum. I appreciate that the authors present both and add the caveat whenever they mention the conclusion. However, without an argument that the relative definition is more appropriate, the fact of the matter is that the evidence is equivocal.

There is also a good reason to think that the *absolute* definition is more appropriate. As expected, participants learn more from credible feedback. Thus, normalizing by average learning (as in the relative definition) amounts to dividing the absolute difference by increasingly large numbers for more credible feedback. If there is a fixed absolute positivity bias (or something that looks like it), the relative bias will necessarily be lower for more credible feedback. In fact, the authors own results demonstrate this phenomenon (see below). A reduction in relative bias thus provides weak evidence for the claim.

It is interesting that the discovery study shows evidence of a drop in absolute bias. However, for me, this just raises questions. Why is there a difference? Was one just a fluke? If so, which one?

Positivity bias or perseveration?

Positivity bias and perseveration will both predict a stronger relationship between positive (vs. negative) feedback and future choice. They can thus be confused for each other when inferred from choice data. This potentially calls into question all the results on positivity bias.

The authors clearly identify this concern in the text and go to considerable lengths to rule it out. However, the new results (in revision 1) show that a perseveration-only model can in fact account for the qualitative pattern in the human data (the CA parameters). This contradicts the current conclusion:

Critically, however, these analyses also confirmed that perseveration cannot account for our main finding of increased positivity bias, relative to the overall extent of CA, for low-credibility feedback.

Figure 24c shows that the credibility-CA model does in fact show stronger positivity bias for less credible feedback. The model distribution for credibility 1 is visibly lower than for credibilities 0.5 and 0.75.

The authors need to be clear that it is the *magnitude* of the effect that the perseveration-only model cannot account for. Furthermore, they should additionally clarify that this is true only for models fit to data; it is possible that the credibility-CA model could capture the full size of the effect with different parameters (which could fit best if the model was implemented slightly differently).

The authors could make the new analyses somewhat stronger by using parameters optimized to capture just the pattern in CA parameters (for example by MSE). This would show that the models are in principle incapable of capturing the effect. However, this would be a marginal improvement because the conclusion would still rest on a quantitative difference that depends on specific modeling assumptions.

New simulations clearly demonstrate the confound in relative bias

Figure 24 also speaks to the relative vs. absolute question. The model without positivity bias shows a slightly *stronger* absolute "positivity bias" for the most credible feedback, but a *weaker* relative bias. This is exactly in line with the logic laid out above. In standard bandit tasks, perseveration can be quite well-captured by a fixed absolute positivity bias, which is roughly what we see in the simulations (I'm not sure what to make of the slight increase; perhaps a useful lead for the authors). However, when we divide by average credit assignment, we now see a reduction. This clearly demonstrates that a reduction in relative bias can emerge without any true differences in positivity bias.

Given everything above, I think it is unlikely that the present data can provide even "solid" evidence for the claim that positivity bias is greater with less credible feedback. This confound could be quickly ruled out, however, by a study in which feedback is sometimes provided in the absence of a choice. This would empirically isolate positivity bias from choice-related effects, including perseveration.

Comments on revisions:

Great work on this. The new paper is very interesting as well. I'm delighted to see that the excessive amount of time I spent on this review has had a concrete impact.

# Author comment: Author response

**DOI:** 10.7554/eLife.106073.4.sa4

**Authors:** Juan Vidal-Perez, Raymond J Dolan, Rani Moran

**Affiliations:** Max Planck Institute of Psychiatry London United Kingdom; University College London London United Kingdom; Queen Mary University of London London United Kingdom

The following is the authors’ response to the previous reviews

**eLife Assessment**

This study provides an important extension of credibility-based learning research with a well-controlled paradigm by showing how feedback reliability can distort reward-learning biases in a disinformation-like bandit task. The strength of evidence is convincing for the core effects reported (greater learning from credible feedback; robust computational accounts, parameter recovery) but incomplete for the specific claims about heightened positivity bias at low credibility, which depend on a single dataset, metric choices (absolute vs relative), and potential perseveration or cueing confounds. Limitations concerning external validity and task-induced cognitive load, and the use of relatively simple Bayesian comparators, suggest that incorporating richer active-inference/HGF benchmarks and designs that dissociate positivity bias from choice history would further strengthen this paper.

We thank the editors and reviewers for a careful assessment.

In response, we have toned down our claims regarding heightened positivity biases, explicitly stating that the findings are equivocal and depend on the scale (i.e., metric) and study (whereas previously we stated our hypothesis was supported). We have also clarified which aspects of the findings extend beyond perseveration. We believe the evidence now presented provides convincing support for this more nuanced claim.

We wish to emphasize that dissociating positivity bias from perseveration is a challenge not just for our work, but for the entire field of behavioral reinforcement learning. In fact, in a recent preprint (Learning asymmetry or perseveration? A critical re-evaluation and solution to a pervasive confound, Vidal-Perez et al., 2025; https://osf.io/preprints/psyarxiv/xdse5_v1) we argue that, to date, all studies claiming evidence for positivity bias beyond perseveration suffered flaws, and that there are currently no robust, behavioral, model-agnostic signatures that dissociate effects of positivity bias from perseveration. While this remains a limitation, we would stress that, relative to the state of the art in the field, our work goes beyond what has previously been reported. We believe this should also be reflected in the assessment of our work.

We elaborate more on these issues in our responses to R3 below.

**Public Reviews:**

**Reviewer #1 (Public review):**

Comments on revisions:

In their updated version the authors have made some edits to address my concerns regarding the framing of the 'normative' bayesian model, clarifying that they utilized a simple bayesian model which is intended to adhere in an idealized manner to the intended task structure, though further simulations would have been ideal.

The authors, however, did not take my recommendation to explore the symptoms in the symptom scales they collected as being a potential source of variability. They note that these were for hypothesis generation and were exploratory, fair enough, but this study is not small and there should have been sufficient sample size for a very reasonable analysis looking at symptom scores.

However, overall the toned down claims and clarifications of intent are adequate responses to my previous review.

We thank the reviewer. We remain convinced that targeted hypotheses tested using betterpowered designs is the most effective way to examine how our findings relate to symptom scales, something we hope to pursue in future studies.

**Reviewer #2 (Public review):**

This important paper studies the problem of learning from feedback given by sources of varying credibility. The convincing combination of experiment and computational modeling helps to pin down properties of learning, while opening unresolved questions for future research.

Summary:

This paper studies the problem of learning from feedback given by sources of varying credibility. Two bandit-style experiments are conducted in which feedback is provided with uncertainty, but from known sources. Bayesian benchmarks are provided to assess normative facets of learning, and alternative credit assignment models are fit for comparison. Some aspects of normativity appear, in addition to possible deviations such as asymmetric updating from positive and negative outcomes.

Strengths:

The paper tackles an important topic, with a relatively clean cognitive perspective. The construction of the experiment enables the use of computational modeling. This helps to pinpoint quantitatively the properties of learning and formally evaluate their impact and importance. The analyses are generally sensible, and advanced parameter recovery analyses (including cross-fitting procedure) provide confidence in the model estimation and comparison. The authors have very thoroughly revised the paper in response to previous comments.

Weaknesses:

The authors acknowledge the potential for cognitive load and the interleaved task structure to play a meaningful role in the results, though leave this for future work. This is entirely reasonable, but remains a limitation in our ability to generalize the results. Broadly, some of the results obtain in cases where the extent of generalization is not always addressed and remains uncertain.

We thank the reviewer once more for a thoughtful assessment of our work.

**Reviewer #3 (Public review):**

Summary

This paper investigates how disinformation affects reward learning processes in the context of a twoarmed bandit task, where feedback is provided by agents with varying reliability (with lying probability explicitly instructed). They find that people learn more from credible sources, but also deviate systematically from optimal Bayesian learning: They learned from uninformative random feedback, learned more from positive feedback, and updated too quickly from fully credible feedback (especially following low-credibility feedback). Overall, this study highlights how misinformation could distort basic reward learning processes, without appeal to higher order social constructs like identity.

Strengths

- The experimental design is simple and well-controlled; in particular, it isolates basic learning processes by abstracting away from social context
- Modeling and statistics meet or exceed standards of rigor
- Limitations are acknowledged where appropriate, especially those regarding external validity - The comparison model, Bayes with biased credibility estimates, is strong; deviations are much more compelling than e.g. a purely optimal model
- The conclusions are of substantial interest from both a theoretical and applied perspective

Weaknesses

The authors have addressed most of my concerns with the initial submission. However, in my view, evidence for the conclusion that less credible feedback yields a stronger positivity bias remains weak. This is due to two issues.

Absolute or relative positivity bias?

The conclusion of greater positivity bias for lower credible feedback (Fig 5) hinges on the specific way in which positivity bias is defined. Specifically, we only see the effect when normalizing the difference in sensitivity to positive vs. negative feedback by the sum. I appreciate that the authors present both and add the caveat whenever they mention the conclusion. However, without an argument that the relative definition is more appropriate, the fact of the matter is that the evidence is equivocal.

We thank the reviewer for an insightful engagement with our manuscript. The reviewer’s comments on the subtle interplay between perseveration and learning asymmetries were so thought-provoking that they have inspired a new article that delves deeply into how gradual choice-perseveration can lead to spurious conclusions about learning asymmetries in Reinforcement Learning (Learning asymmetry or perseveration? A critical re-evaluation and solution to a pervasive confound, Vidal-Perez et al., 2025; https://osf.io/preprints/psyarxiv/xdse5_v1).

To the point- we agree with the reviewer the evidence for this hypothesis is equivocal, and we took on board the suggestion to tone down our interpretation of the findings. We now state explicitly, both in the results section (“Positivity bias in learning and credibility”) and in the Discussion, that the results provide equivocal support for our hypothesis:

RESULTS

“However, we found evidence for agent-based modulation of positivity bias when this bias was measured in relative terms. Here we calculated, for each participant and agent, a relative Valence Bias Index (rVBI) as the difference between the Credit Assignment for positive feedback (CA+) and negative feedback (CA-), relative to the overall magnitude of CA (i.e., |CA+| + |CA-|) (Fig. 5c). Using a mixed effects model, we regressed rVBIs on their associated credibility (see Methods), revealing a relative positivity bias for all credibility levels [overall rVBI (b=0.32, F(1,609)=68.16), 50% credibility (b=0.39, t(609)=8.00), 75% credibility (b=0.41, F(1,609)=73.48) and 100% credibility (b=0.17, F(1,609)=12.62), all p’s<0.001]. Critically, the rVBI varied depending on the credibility of feedback (F(2,609)=14.83, p<0.001), such that the rVBI for the 3-star agent was lower than that for both the 1-star (b=-0.22, t(609)=-4.41, p<0.001) and 2-start agent (b=-0.24, F(1,609)=24.74, p<0.001). Feedback with 50% and 75% credibility yielded similar rVBI values (b=0.028, t(609)=0.56,p=0.57). Finally, a positivity bias could not stem from a Bayesian strategy as both Bayesian models predicted a negativity bias (Fig. 5b-c; Fig. S8; and SI 3.1.1.3 Table S11-S12, 3.2.1.1, and 3.2.1.2). Taken together, this provides equivocal support for our initial hypothesis, depending on the measurement scale used to assess the effect (absolute or relative).”

“Previous research has suggested that positivity bias may spuriously arise from pure choice-perseveration (i.e., a tendency to repeat previous choices regardless of outcome) (49–51). While our models included a perseveration-component, this control may not be perfect. Therefore, in additional control analyses, we generated (using ex-post simulations based on best fitting parameters) synthetic datasets using models including choice-perseveration but devoid of feedback-valence bias, and fitted them with our credibilityvalence model (see SI 3.6.1). These analyses confirmed that a pure perseveration account can masquerade as an apparent positivity bias and even predict the qualitative pattern of results related to credibility (i.e., a higher relative positivity bias for low-credibility feedback). Critically, however, this account consistently predicted a reduced magnitude of credibility-effect on relative positivity bias as compared to the one we observed in participants, suggesting some of the relative amplification of positivity bias goes above and beyond a contribution from perseveration.”

DISCUSSION

“Previous reinforcement learning studies, report greater credit-assignment based on positive compared to negative feedback, albeit only in the context of veridical feedback (43,44,63). Here, we investigated whether a positivity bias is amplified for information of low credibility, but our findings are equivocal and vary as a function of scaling (absolute or relative) and study. We observe selective absolute amplification of a positivity bias for information of low and intermediate credibility in the discovery study alone. In contrast, we find a relative (to the overall extent of CA) amplification of confirmation bias in both studies. Importantly, the magnitude of these amplification effects cannot be reproduced in ex-post simulations of a model incorporating simple choice perseveration without an explicit positivity bias, suggesting that at least part of the amplification reflects a genuine increase in positivity bias.”

There is also a good reason to think that the *absolute* definition is more appropriate. As expected, participants learn more from credible feedback. Thus, normalizing by average learning (as in the relative definition) amounts to dividing the absolute difference by increasingly large numbers for more credible feedback. If there is a fixed absolute positivity bias (or something that looks like it), the relative bias will necessarily be lower for more credible feedback. In fact, the authors own results demonstrate this phenomenon (see below). A reduction in relative bias thus provides weak evidence for the claim.

We agree with the reviewer that absolute and relative measures can yield conflicting impressions. To some extent, this is precisely why we report both (i.e., if the two would necessarily agree, reporting both would be redundant). However, we are unconvinced that one measure is inherently more appropriate than the other. In our view, both are valid as long as they are interpreted carefully and in the right context. To illustrate, consider salary changes, which can be expressed on either an absolute or a relative scale. If Bob’s £100 salary increases to £120 and Alice’s £1000 salary increases to £1050, then Bob’s raise is absolutely smaller but relatively larger. Is one measure more appropriate than the other? Economists would argue not; rather, the choice of scale depends on the question at hand.

In the same spirit, we have aimed to be as clear and transparent as possible in stating that (1) in the main study, there is no effect in the absolute sense, and (2) framing positivity bias in relative terms is akin to expressing it as a percentage change.

It is interesting that the discovery study shows evidence of a drop in absolute bias. However, for me, this just raises questions. Why is there a difference? Was one a just a fluke? If so, which one?

We are unsure why we didn’t find absolute amplification effect within the main studies. However, we don’t think the results from the preliminary study were just a ‘fluke’. We have recently conducted two new studies (in preparation for publication), where we have been able to replicate the finding of increased positivity bias for lower-credibility sources in both absolute and relative terms. We agree current results leave unresolved questions and we hope to follow up on these in the near future.

Positivity bias or perseveration?

Positivity bias and perseveration will both predict a stronger relationship between positive (vs. negative) feedback and future choice. They can thus be confused for each other when inferred from choice data. This potentially calls into question all the results on positivity bias.

The authors clearly identify this concern in the text and go to considerable lengths to rule it out. However, the new results (in revision 1) show that a perseveration-only model can in fact account for the qualitative pattern in the human data (the CA parameters). This contradicts the current conclusion:

Critically, however, these analyses also confirmed that perseveration cannot account for our main finding of increased positivity bias, relative to the overall extent of CA, for low-credibility feedback.

Figure 24c shows that the credibility-CA model does in fact show stronger positivity bias for less credible feedback. The model distribution for credibility 1 is visibly lower than for credibilities 0.5 and 0.75.

The authors need to be clear that it is the *magnitude* of the effect that the perseveration-only model cannot account for. Furthermore, they should additionally clarify that this is true only for models fit to data; it is possible that the credibility-CA model could capture the full size of the effect with different parameters (which could fit best if the model was implemented slightly differently).

The authors could make the new analyses somewhat stronger by using parameters optimized to capture just the pattern in CA parameters (for example by MSE). This would show that the models are in principle incapable of capturing the effect. However, this would be a marginal improvement because the conclusion would still rest on a quantitative difference that depends on specific modeling assumptions.

We thank the reviewer for raising this important point. We agree our original wording could have been more carefully formulated and are grateful for this opportunity to refine this. The reviewer is correct that a model with only perseveration can qualitatively reproduce the pattern of increased relative positivity bias for less credible feedback in the main study (but not in the discovery study), and our previous text did not acknowledge this. As stated in the previous section, we have revised the manuscript (in the Results, Discussion, and SI) to ensure we address this in full. Our revised text now makes it explicit that while a pure perseveration account predicts the qualitative pattern, it does not predict the magnitude of the effects we observe in our data.

RESULTS

“Previous research has suggested that positivity bias may spuriously arise from pure choice-perseveration (i.e., a tendency to repeat previous choices regardless of outcome) (49–51). While our models included a perseveration-component, we acknowledge this control is not perfect. Therefore, in additional control analyses, we generated (using ex-post simulations based on best fitting parameters) synthetic datasets using models including choice-perseveration, but devoid of feedback-valence bias, and fitted these with our credibility-valence model (see SI 3.6.1). These analyses confirmed that a pure perseveration account can masquerade as an apparent positivity bias, and even predict the qualitative pattern of results related to credibility (i.e., a higher relative positivity bias for low-credibility feedback). Critically, however, this account consistently predicted a reduced magnitude of credibility-effect on relative positivity bias as compared to the one we observed in participants, suggesting at least some of the relative amplification of positivity bias goes above and beyond contributions from perseveration.”

DISCUSSION

“Previous reinforcement learning studies, report greater credit-assignment based on positive compared to negative feedback, albeit only in the context of veridical feedback (43,44,63). Here, we investigated whether a positivity bias is amplified for information of low credibility, but our findings on this matter were equivocal and varied as a function of scaling (absolute or relative) and study. We observe selective absolute amplification of the positivity bias for information of low and intermediate credibility in the discovery study only. In contrast, we find a relative (to the overall extent of CA) amplification of confirmation bias in both studies. Importantly, the magnitude of these amplification effects cannot be reproduced in ex-post simulations of a model incorporating simple choice perseveration without an explicit positivity bias, suggesting that at least part of the amplification reflects a genuine increase in positivity bias.”

SI (3.6.1)

“Interestingly, a pure perseveration account predicted an amplification of the relative positivity bias under low (compared to full) credibility (with the two rightmost histograms in Fig. S24d falling in the positive range). However, the magnitude of this effect was significantly smaller than the empirical effect (as the bulk of these same histograms lies below the green points). Moreover, this account predicted a negative amplification (i.e., attenuation) of an absolute positivity bias, which was again significantly smaller than the empirical effect (see corresponding histograms in S24b). This pattern raises an intriguing possibility that perseveration may, at least partially, mask a true amplification of absolute positivity bias.”

Furthermore, our revisions make it now explicit that these analyses are based on ex-post simulations using the model best-fitting parameters. We do not argue that this pattern can’t be captured by other parameters crafted specifically to capture this pattern. However, we believe that the ex-post fitting is the best practice to check whether a model can produce an effect of interest (see for example The Importance of Falsification in Computational Cognitive Modeling, Palminteri et al., 2017; here). Based on this we agree with the reviewer the benefit from the suggested additional analyses is minimal.

New simulations clearly demonstrate the confound in relative bias

Figure 24 also speaks to the relative vs. absolute question. The model without positivity bias shows a slightly *stronger* absolute "positivity bias" for the most credible feedback, but a *weaker* relative bias. This is exactly in line with the logic laid out above. In standard bandit tasks, perseveration can be quite well-captured by a fixed absolute positivity bias, which is roughly what we see in the simulations (I'm not sure what to make of the slight increase; perhaps a useful lead for the authors). However, when we divide by average credit assignment, we now see a reduction. This clearly demonstrates that a reduction in relative bias can emerge without any true differences in positivity bias.

This relates back to the earlier point about scaling. However, we wish to clarify that this is not a confound in the usual sense i.e., an external variable that varies systematically with the independent variable (credibility) and influences the dependent variable (positivity bias), thereby undermining causal inference. Rather, we consider it is a scaling issue: measuring absolute versus relative changes in the same variable can yield conflicting impressions.

Given everything above, I think it is unlikely that the present data can provide even "solid" evidence for the claim that positivity bias is greater with less credible feedback. This confound could be quickly ruled out, however, by a study in which feedback is sometimes provided in the absence of a choice. This would empirically isolate positivity bias from choice-related effects, including perseveration.

We trust our responses make clear we have tempered our claims and stated explicitly where a conclusion is equivocal. We believe we have convincing evidence for a nuanced claim regarding how credibility affects positivity bias.

We are grateful for the reviewer’s suggestion of a study design to empirically isolate positivity bias from choice-related effects. We have considered this carefully, but do not believe the issue is as straightforward as suggested. As we understand it, the suggestion assumes that positivity bias should persist when people process feedback in the absence of choice (where perseverative tendencies would not be elicited). While this is possible, there is existing work that indicates otherwise. In particular, Chambon et al. (2020, Nature Human Behavior) compared learning following free versus forced choices and found that learning asymmetries, including a positivity bias, were selectively evident in free-choice trials but not in forced-choice trials. This implies that a positivity bias is intricately tied to the act of choosing, rather than a general learning artifact that emerges independently of choice context. This is further supported by arguments that the positivity bias in reinforcement learning is better understood as a form of confirmation bias, whereby feedback confirming a choice is weighted more heavily (Palminteri et al., 2017, Plos Comp. Bio.). In other words, it is unclear whether one should expect positivity/confirmation bias to emerge when feedback is provided in the absence of choice.

That said, we agree fully with a need to have task designs that better dissociate positivity bias from perseveration. We now acknowledge in our Discussion that such designs can benefit future studies on this topic:

Future studies could also benefit from using designs that are better suited for dissociating learning asymmetries from gradual perseveration (51).

We hope to be able to pursue this direction in the future.

**Recommendations for the Authors:**

I greatly appreciate the care with which you responded to my comments. I'm sorry that I can't improve my overall evaluation, given the seriousness of the concerns in the public review (which the new results have unfortunately bolstered more than assuaged). If it were me, I would definitely collect more data because both issues could very likely be strongly addressed with slight modifications of the current task.

Alternatively, you could just dramatically de-emphasize the claim that positivity bias is higher for less credible feedback. I will be sad because it was my favorite result, but you have many other strong results, and I would still label the paper "important" without this one.

We thank the reviewer for an exceptionally thorough and insightful engagement with our manuscript. Your meticulous attention to detail, and sharp conceptual critiques, have been invaluable, and our paper is immeasurably stronger and more rigorous as a direct result of this input. Indeed, the referee’s comments inspired us to prepare a new article that delves deeply into the confound of dissociating between gradual choice-perseveration and learning asymmetries in RL (Learning asymmetry or perseveration? A critical re-evaluation and solution to a pervasive confound, Vidal-Perez et al., 2025; https://osf.io/preprints/psyarxiv/xdse5_v1).

Specifically, in this new paper we address the point that dissociating positivity bias from perseveration is a challenge not just for our work, but for the entire field of behavioral reinforcement learning. In fact, we argue that all studies claiming evidence for positivity bias, over and above an effect of perseveration, are subject to flaws, including being biased to find evidence for positivity/confirmation bias. Furthermore, we agree with the reviewer’s wish to see modelagnostic support and note there are currently no robust, behavioral, model-agnostic signatures implicating positivity bias over and above an effect of perseveration. While this remains an acknowledged limitation within our current work, we trust the reviewer will agree that relative to other efforts in the field, our current work pushes the boundary and takes several important steps beyond what has previously been done in this area.

Below are some minor notes, mostly on the new content-hopefully easy; please don't put much time into addressing these!

Main text

where individuals preferably learn from . Perhaps "preferentially"?

The text has been modified to accommodate the reviewer’s comment:

“Additionally, in both experiments, participants exhibited increased learning from trustworthy information when it was preceded by non-credible information and an amplified normalized positivity bias for noncredible sources, where individuals preferentially learn from positive compared to negative feedback (relative to the overall extent of learning).”

One interpretation of this model is as a "sophisticated" logistic ... the CA parameters take the role of "regression coefficients"

Consider removing "sophisticated" and also the quotations around "regression coefficients". This came across as unprofessional to me.

The text has been modified to accommodate the reviewer’s comment:

“The probability to choose a bandit (say A over B) in this family of models is a logistic function of the contrast choice-propensities between these two bandits. One interpretation of this model is as a logistic regression, where the CA parameters take the role of regression coefficients corresponding to the change in log odds of repeating the just-taken action in future trials based on the feedback (+/- CA for positive or negative feedback, respectively; the model also includes gradual perseveration which allows for constant log-odd changes that are not affected by choice feedback).”

These models operate as our instructed-credibility and free-credibility Bayesian models, but also incorporate a perseveration values, updated in each trial as in our CA models (Eqs. 3 and 5).

Is Eq 3 supposed to be Eq 4 here? I don't see how Eq 3 is relevant. Relatedly, please use a variable other than P for perseveration because P(chosen) reads as "probability chosen" - and you actually use P in latter sense in e.g. Eq 11

The text has been modified to accommodate the reviewer’s comment. P values have been changed to Pers and P(bandit) has been replaced by Prob(bandit). “All models also included gradual perseveration for each bandit. In each trial the perseveration values (Pers) were updated according to

$\operatorname{Pers}(\mathrm{chosen}) \leftarrow\left(1-f_{P}\right) * \operatorname{Pers}(\mathrm{chosen})+\operatorname{PERS}$

Where PERS is a free parameter representing the P-value change for the chosen bandit, and fP (Î[0,1]) is the free parameter denoting the forgetting rate applied to the Pers value. Additionally, the Pers-values of all the non-chosen bandits (i.e., again, the unchosen bandit of the current pair, and all the bandits from the not-shown pairs) were forgotten as follows:

$\operatorname{Pers}(n o n-\mathrm{chosen}) \leftarrow\left(1-f_{p}\right) * \operatorname{Pers}(n o n-\mathrm{chosen})$

We modelled choices using a softmax decision rule, representing the probability of the participant to choose a given bandit over the alternative:

$\operatorname{Prob}(\mathrm{bandit})=\frac{1}{1+e^{[Q(\text{other bandit})-Q(\mathrm{bandit})]+[\mathrm{Pers}(\text{other bandit})-\mathrm{Pers}(\mathrm{bandit})]}}$

SI

Figure 24 and Figure 26: in the x tick labels, consider using e.g. "0.5 vs 1" rather than "0.5-1". I initially read this as a bin range.

We thank the reviewer for pointing this out. Our intention was to denote a direct subtraction (i.e., the effect for 0.5 credibility minus the effect for 1.0 credibility). We were concerned that not noting the subtraction might confuse readers about the direction of the plotted effect. We have clarified this in the figure legends:

“Figure 24: Predicted positivity bias results for participants and for simulations of the Credibility-CA (including perseveration, but no valence-bias component). a, Valence bias results measured in absolute terms (by regressing the ML CA parameters, on their associated valence and credibility). b, Difference in positivity bias (measured in absolute terms) across credibility levels. On the x-axis, the hyphen (-) represents subtraction, such that a label of '0.5-1' indicates the difference in the measurement for the 0.5 and 1.0 credibility conditions. Such differences are again based in the same mixed effects model as plot a. The inflation of aVBI for lower-credibility agents is larger than the one predicted by a pure perseveration account. c, Valence bias results measured in relative terms (by regressing the rVBIs on their associated credibility). Participants present a higher rVBI than what would be predicted by a perseveration account (except for the completely credible agent). d, Difference in rVBI across credibility levels. Such differences are again based in the same mixed effects model as plot c. The inflation of rVBI for lower-credibility agents is larger than the one predicted by a pure perseveration account. Histograms depict the distribution of coefficients from 101 simulated group-level datasets generated by the Credibility-CA model and fitted with the Credibility-Valence CA model. Gray circles represent the mean coefficient from these simulations, while black/green circles show the actual regression coefficients from participant behaviour (green for significant effects in participants, black for non-significant). Significance markers (* p<.05, ** p<.01) indicate that fewer than 5% or 1% of simulated datasets, respectively, predicted an effect as strong as or stronger than that observed in participants, and in the same direction as the participant effect.”

However, importantly, these simulations did not predict a change in the level of positivity bias as a function of feedback credibility

You're confirming the null hypothesis here; running more simulations would likely yield a significant effect. The simulation shows a pretty clear pattern of increasing positivity bias with higher credibility. Crucially, this is the opposite of what people show. Please adjust the language accordingly.

The text has been modified to accommodate the reviewer’s comment.

“However, importantly, these simulations did not reveal a significant change in the level of positivity bias as a function of feedback credibility, neither at an absolute level (F(3,412)=1.43,p=0.24), nor at a relative level (F(3,412)=2.06,p=0.13) (Fig. S25a-c). Numerically, the trend was towards an increasing (rather than decreasing) positivity bias as a function of credibility.”

More importantly, the inflation in positivity bias for lower credibility feedback is substantially higher in participants than what would be predicted by a pure perseveration account, a finding that holds true for both absolute (Fig. S24b) and relative (Fig. S24d) measures.

A statistical test would be nice here, e.g. a regression like

rVBI ~ credibility_1 * is_model

. Alternatively, clearly state what to look for in the figure, where it is pretty clear when you know exactly what you're looking for.

The text has been modified to make sure that the figure is easier to interpret (we pointed out to readers what they should look at):

“Interestingly, a pure perseveration account predicted an amplification of the relative positivity bias under low (compared to full) credibility (with the two rightmost histograms in Fig. S24c falling in the positive range). However, the magnitude of this effect was significantly smaller than the empirical effect (as the bulk of these same histograms lies below the green points). Moreover, this account predicted a negative amplification (i.e., attenuation) of an absolute positivity bias, which was again significantly smaller than the empirical effect (see corresponding histograms in S24b). This pattern raises an intriguing possibility that perseveration may partially mask a true amplification of absolute positivity bias.”
